# Multi-omics Mendelian randomization identifies mitochondrial genes associated with immune microenvironment signatures in endometriosis

**DOI:** 10.3389/frph.2026.1747031

**Published:** 2026-05-04

**Authors:** Sha Wang, Xiaoyu Ji, Maako Moriyama, Yukihiro Azuma, Ikumi Wada, Tasuku Harada, Fuminori Taniguchi

**Affiliations:** 1 Department of Minimally Invasive Gynecology, Beijing Obstetrics and Gynecology Hospital, Capital Medical University, Beijing Maternal and Child Health Care Hospital, Beijing, China; 2 Division of Obstetrics and Gynecology, Tottori University Faculty of Medicine, Yonago, Japan; 3 Department of Obstetrics and Gynecology, Women and Children’s Hospital, Qingdao University, Qingdao, China

**Keywords:** endometriosis, eQTL, GWAS, Mendelian randomization, mitochondrial, mQTL, multiomics

## Abstract

**Background:**

Endometriosis is a complex gynaecological disorder that affects 10%–15% of reproductive-age women and is characterized by chronic inflammation, pelvic pain, and infertility. Although mitochondrial dysfunction is implicated in endometriosis pathogenesis, the causal relationships between mitochondrial genes and endometriosis remain unclear. This study aims to elucidate these relationships through multiomics bioinformatics analysis and experimental validation.

**Methods:**

We analysed 1,133 mitochondrial-related genes from the MitoCarta3.0 database, incorporating eQTL, pQTL, and mQTL data from the eQTLGen, DECODE, and Brisbane databases. Two-sample Mendelian randomization and summary data Mendelian randomization were performed using FinnGen endometriosis GWAS data to establish causal relationships. Protein–protein interaction networks were constructed, followed by GO/KEGG functional enrichment analysis. Machine learning algorithms, including LASSO, random forest, and Boruta, were applied to the GSE51981 and GSE7305 datasets for feature gene selection. Single-sample gene set enrichment analysis was used to assess correlations with immune cells. Additionally, immunohistochemical validation was performed on endometrial samples from endometriosis patients and controls.

**Results:**

Mendelian randomization revealed 128 mitochondrial genes with significant causal relationships to endometriosis that were significantly enriched in the steroid biosynthesis, fatty acid elongation, and mitochondrial gene expression pathways. Five key feature genes (PHYH, GPD2, C12orf65, MRPS6, and RPL21) were selected, with GPD2 and MRPS6 demonstrating preliminary predictive value (AUC > 0.6) in both the training and validation cohorts. These genes were significantly associated with macrophages and NK cells, suggesting that mitochondrial dysfunction may contribute to endometriosis through immune modulation. Immunohistochemical validation confirmed high expression of the marker genes GPD2 and MRPS6, which was consistent with the transcriptomic findings.

**Conclusions:**

This study systematically elucidated the causal roles of mitochondrial genes in endometriosis, identifying GPD2 and MRPS6 as potential exploratory biomarkers and therapeutic targets. These findings provide a foundation for precision diagnostics and targeted therapeutic strategies for endometriosis management.

## Introduction

Endometriosis (EMs) is a complex gynaecological disorder characterized by the abnormal growth of endometrial-like tissue outside the uterine, leading to many symptoms, including chronic inflammation, pain, and infertility (1). Although the pathogenesis of EMs remains incompletely understood, current research indicates that the interplay among hormonal, neurological and immunological factors plays a crucial role in disease development (2). Epidemiological studies have shown that EMs affects approximately 10%–15% of women of reproductive age worldwide, significantly impacting the quality of life of patients while imposing substantial burdens on healthcare systems. However, owing to its complex pathological mechanisms and symptom heterogeneity, early diagnosis and precision treatment of EMs continue to face significant challenges (3).

In recent years, the association between mitochondrial dysfunction and various gynecological and reproductive diseases such as polycystic ovary syndrome, ovarian cancer, and poor ovarian response has gradually drawn the attention of researchers (4–6). Mitochondria are essential organelles that determine intracellular metabolic pathways and participate in critical functions, including cellular respiration, adenosine triphosphate (ATP) production, reactive oxygen species (ROS) generation, activation of the caspase family of proteases, and regulation of apoptosis, necrosis, and autophagy (7). Under pathological conditions, disruption of mitochondrial homeostasis leads to ROS generation and energy deficiency. It has been reported that EMs evolves a unique mitochondrial energy production and metabolism to counteract hypoxia and oxidative phosphorylation (8). Disruption of mitochondrial activity affects numerous physiological processes, including cell death, inflammatory responses, and cell cycle regulation, thereby exacerbating the symptoms of EMs (9). For instance, increased mitochondrial stress and reactive oxygen species production in ectopic cells upregulate the expression of pro-inflammatory factors, enhancing their malignant characteristics of proliferation and invasion, playing a crucial role in the occurrence and development of EMs (10). However, research on the specific mechanisms involved remains limited.

Although traditional observational studies have identified multiple genetic loci associated with EMs, they have largely been unable to establish true causal relationships because of the presence of confounding factors and reverse causality. Mendelian randomization (MR), as a causal inference method that uses genetic variants as instrumental variables, can effectively reduce the influence of confounding factors and provide reliable evidence for establishing causal relationships between exposures and outcomes (11). Methods such as two-sample Mendelian randomization (TSMR) and summary data-based Mendelian randomization (SMR) have made exploring disease mechanisms at the molecular level possible.

With the rapid development of multiomics technologies, the integration of multilevel molecular data, including gene expression, protein expression, and DNA methylation data, can more comprehensively reveal disease molecular mechanisms (12–14). The application of machine learning algorithms in biomedical research provides powerful tools for identifying key feature genes and constructing predictive models from massive amounts of biological data. Moreover, the emergence of single-cell analysis technology has made exploring gene function and intercellular interactions at the cell type-specific level possible, providing new perspectives for understanding the cellular and molecular mechanisms of diseases (15).

The role of the immune microenvironment in the pathogenesis of EMs has received increasing attention (16). Immune cell infiltration patterns around ectopic endometrial tissues are closely related to disease occurrence and development (17). As the centre of cellular energy metabolism, mitochondrial functional status may influence local tumour microenvironments by regulating immune cell activation, differentiation, and function (18, 19). However, the interplay between mitochondrial genes and the immune microenvironment in the context of EMs remains underexplored.

Building on this background, we applied a multiomics bioinformatics framework that integrated 1,133 mitochondria-related genes from the MitoCarta3.0 database with large-scale genome-wide association study (GWAS) summary statistics. Using TSMR and SMR, we inferred causal links between mitochondrial genes and EMs. We then characterized the causally implicated genes through protein–protein interaction network construction and functional enrichment analyses. To identify robust diagnostic candidates, we employed machine learning algorithms for feature selection and validated their performance in independent cohorts. Finally, single-cell transcriptomic analyses revealed the cell type-specific expression patterns and functional relevance of key genes. Collectively, the results of this study provide mechanistic insights into the mitochondrial underpinnings of EMs and lay a foundation for novel biomarker development and targeted therapeutic strategies.

## Materials and methods

### Study design

This study employed a two-stage design to systematically investigate the role of mitochondrial genes in EMs. In the discovery phase, we utilized large-scale multi-omics data (GWAS, eQTL, pQTL, and mQTL) and machine learning algorithms to screen for candidate mitochondrial genes with causal relationships to EMs. In the validation phase, we collected clinical tissue samples to verify the protein expression levels of the identified exploratory biomarkers using immunohistochemistry.

### Data sources and quality control

**Figure 1:**
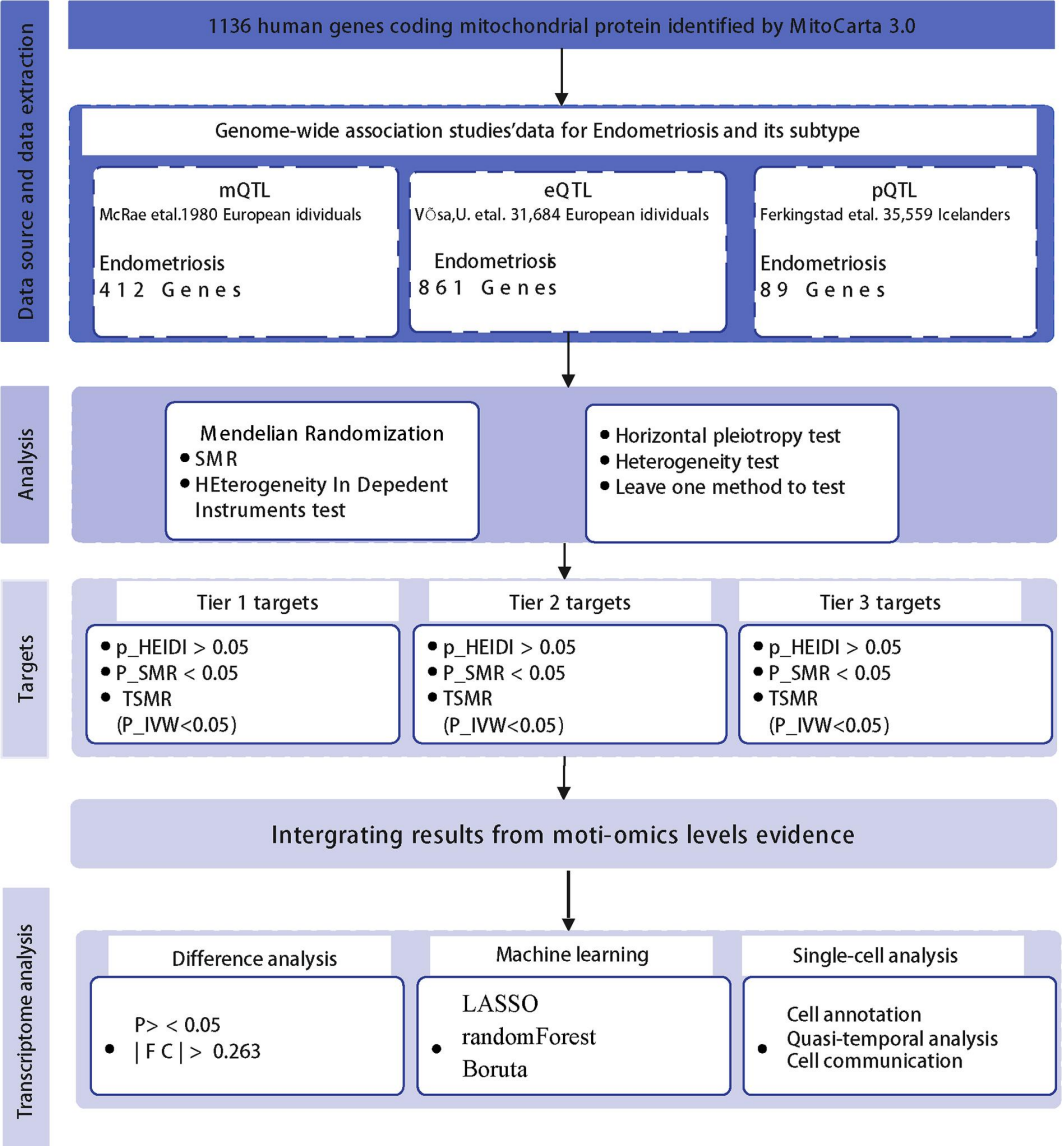
Flow diagram of the study.

A total of 1,133 mitochondria-related genes were obtained from the MitoCarta3.0 database (20). The study workflow is illustrated in Figure 1.

Expression quantitative trait locus (eQTL) data were obtained from the eQTLGen Consortium, which focuses on investigating the genetic architecture of blood gene expression and understanding the genetic basis of complex traits. Methylation quantitative trait locus (mQTL) data were derived through meta-analysis integrating the Brisbane Systems Genetics Study (*n* = 614) and the Lothian Birth Cohorts of 1,921 and 1,936 (*n* = 1,366), utilizing summary statistics from two meta-analysis cohorts (*n* = 1,980) to extract cis-mQTLs of genetic variants closely associated with target genes (association *P* value <5 × 10^−8^) (21). Protein quantitative trait locus (pQTL) data were sourced from the DECODE database, which identified 28,191 genetic associations for 4,907 aptamers in 35,559 Icelanders (*P* value <1.8 × 10^−9^) (22). EMs GWAS summary statistics were obtained from the FinnGen database R10 release, comprising 16,588 cases and 111,583 controls (23).

### Instrumental variable quality control criteria

The following quality control standards were applied: (1) association analysis *P* value <5 × 10^−8^; (2) linkage disequilibrium (LD) clumping based on 1,000 Genomes Project European population data (*r*^2^ < 0.001, distance > 10,000 kb); (3) retention of instrumental variables with *F* statistic >10; and (4) harmonization of exposure and outcome instrumental variables using the harmonize data function.

### Summary data-based Mendelian randomization (SMR) analysis

SMR analysis was conducted using SMR software (version 1.3.1) (24). Genetic variants were employed as instrumental variables, with gene expression levels, DNA methylation levels, or protein abundance serving as exposure factors and disease risk as the outcome variable. The SMR test threshold was set at P_SMR < 0.05, with a HEIDI test P_HEIDI > 0.05 to exclude linkage-induced associations (25).

### Two-sample Mendelian randomization (TSMR) analysis

Analysis was conducted using the R package TwoSampleMR (version 0.5.10) (26). When the SNP count was ≥2, five methods were employed—MR–Egger, weighted median, inverse variance weighted (IVW), simple mode, and weighted mode—with the IVW results being conclusive. When only one SNP was available, the Wald ratio method was used. Cochran's *Q* test was used to assess heterogeneity (27), and MR–Egger regression was used to test for horizontal pleiotropy (28). For the significant genetic associations identified through MR, we performed the MR Steiger directionality test to verify the causal direction and rule out potential reverse causation. A result indicating “causal direction = Steiger test failed” implies the absence of SNPs driving reverse causality, thus allowing all instrumental variables to be retained for further analysis. Furthermore, to evaluate whether any single IV disproportionately drove the overall causal effect on the outcome, we conducted a leave-one-out sensitivity analysis. This process involved sequentially omitting each SNP and recalculating the meta-effect of the remaining SNPs using the inverse variance weighted (IVW) method to determine the stability of the results. Finally, the results of this sensitivity analysis were visualized using forest plots.

### Expression profile data acquisition and processing

The GSE51981 dataset was downloaded from the NCBI Gene Expression Omnibus (GEO) database as the training set (77 cases, 71 controls), and GSE7305 was used as the validation set (10 cases, 10 controls), both of which utilized the GPL570 platform. The single-cell dataset GSE213216 (4 normal samples and 9 disease samples) was obtained using the GPL24676 platform (29). Probes were converted to gene symbols, and for genes with multiple probes, the mean expression value was calculated.

### Construction of protein–protein interaction networks and functional enrichment

Protein–protein interaction (PPI) networks were constructed using the STRING database (version 10.0) with a PPI confidence score threshold of 0.15 (30). Gene Ontology (GO) and Kyoto Encyclopedia of Genes and Genomes (KEGG) pathway enrichment analyses were performed using the R package clusterProfiler (version 4.10.0), with an adjusted *P* < 0.05 as the threshold for significant enrichment (31–33).

### Differential gene expression analysis

Differential expression analysis of causally related genes was conducted using the limma package, with screening criteria of a *P* value <0.05 and a |log fold change (logFC)| > 0.263. Box plots and heatmaps were generated to visualize the results.

### Machine learning-based feature selection

Three machine learning algorithms were employed for feature gene selection: (1) least absolute shrinkage and selection operator (LASSO) regression (glmnet package, version 4.1.3) (34); (2) the random forest algorithm (randomForest package, version 4.7–1.1); and (3) the Boruta algorithm (Boruta package, version 8.0) (35). The intersection of the results from all three methods was designated as candidate feature genes.

### Feature gene validation and performance assessment

The limma package was used to validate the differential expression of feature genes in both the training and validation sets. Receiver operating characteristic (ROC) curves were plotted, and area under the curve (AUC) values were calculated using the pROC package (version 1.18.4) (36). Genes demonstrating consistent expression patterns across all the datasets, significant differences (*P* < 0.05, |logFC| > 0.263), and AUC > 0.6 were selected as the final feature genes, indicates potential diagnostic value.

### Immune microenvironment analysis

The single-sample gene set enrichment analysis (ssGSEA) method (GSVA package, version 1.46.0) was employed to calculate infiltration scores for 28 immune cell types (37). It is important to clarify that the reference gene sets utilized for this evaluation are standard, general-purpose immune cell signatures. These are fundamental, pan-tissue immune markers designed to capture broad immune cell populations, and they are neither tumor-oriented nor specifically tailored to the endometrial microenvironment. Student's *t* test was used to compare immune cell infiltration differences between the case and control groups, and Pearson correlation analysis was performed to assess correlations between feature genes and immune cell populations.

### Small-molecule drug prediction

The DrugBank database was used to predict small-molecule drugs related to feature genes (38). Three-dimensional drug structures were obtained from the PubChem database, protein structures were predicted using AlphaFold (version 2.0) (39), molecular docking was performed using CB-Dock (version 1.0) (40), and the results were visualized using PyMOL software (version 3.0) (41).

### Molecular dynamics simulation

Based on the molecular docking results, the complex exhibiting the highest binding affinity was selected for a 100-ns all-atom molecular dynamics (MD) simulation using GROMACS (https://www.gromacs.org/) on a Linux server. The AMBER99SB force field was applied to the protein, while the ligand topology parameters were generated using Sobtop (Tian Lu, Sobtop, Version 1.0 dev5, http://sobereva.com/soft/Sobtop). The system was solvated with the TIP3P water model in a cubic simulation box with a 10 Å distance from the box edges, and sodium (Na⁺) and chloride (Cl⁻) ions were added to neutralize the system charge. Following energy minimization utilizing the steepest descent and conjugate gradient algorithms, the system was equilibrated under the NVT ensemble at 300 K for 100 ps, followed by the NPT ensemble at 1 bar for 100 ps. Subsequently, a 100-ns production MD simulation was conducted. The resulting trajectory data were visualized using R software (version 4.5.2). To verify the binding stability of the complex, key metrics were systematically analyzed, including the root mean square deviation (RMSD), root mean square fluctuation (RMSF), radius of gyration (Rg), hydrogen bond number, solvent accessible surface area (SASA), and the free energy landscape (FEL) based on RMSD and Rg.

### Single-cell RNA sequencing analysis

The single-cell RNA sequencing (scRNA-seq) dataset GSE213216 was analysed using the Seurat package (version 5.1.0). The cell quality control criteria included a gene count >200, total gene count <7,000, and mitochondrial gene percentage <20%. The Harmony package (version 1.2.0) was used to remove batch effects, the top 2,000 highly variable genes were identified, and uniform manifold approximation and projection (UMAP) dimensionality reduction clustering was performed. Cell type annotation was conducted using the SingleR package.

Pseudotime analysis was performed using the Monocle package (version 2.32.0) to infer cell differentiation trajectories. The CellChat package (version 1.6.1) was used to analyse intercellular communication and generate communication networks and ligand–receptor interaction heatmaps. The feature gene scores of individual cells were quantified using the ssGSEA algorithm to compare intergroup differences.

### Sample collection and immunohistochemical analysis

To strictly validate the protein expression patterns of the key feature genes identified in the discovery phase, we recruited an independent clinical cohort for immunohistochemical analysis. Eutopic and ectopic endometrial samples were obtained from 8 patients with Stage IV EMs who underwent surgical treatment at Beijing Obstetrics and Gynecology Hospital, Capital Medical University, from March to September 2024. Control endometrial samples were collected from 8 age-matched non-EMs patients who underwent surgical procedures during the same period. To minimize potential confounding factors, all endometrial tissue samples were collected from patients during the proliferative phase. This study was approved by the local research and ethics committee of Beijing Obstetrics and Gynaecology Hospital (No. 2022-KY-016-01). Written informed consent was obtained from each patient before sampling.

All the samples were fixed in 10% neutral buffered formalin and embedded in paraffin. For immunohistochemical staining, paraffin-embedded sections (4 μm thick) were deparaffinized in xylene and rehydrated through graded ethanol solutions. Antigen retrieval was performed using 3% citric acid buffer (pH: 6.0) under high pressure for 10 min, followed by natural cooling to room temperature. Endogenous peroxidase activity was blocked by incubating the sections with 3% methanol–hydrogen peroxide solution for 20 min at room temperature. After the cells were washed with phosphate-buffered saline (PBS), nonspecific binding was blocked with 5% bovine serum albumin (BSA; Boster Biological Technology, Wuhan, China; AR0004) for 30 min at room temperature. The sections were then incubated overnight at 4 °C with primary antibodies against MRPS6 (1:120 dilution; Proteintech, Wuhan, China; 16273-1-AP) and GPD2 (1:130 dilution; Proteintech, Wuhan, China; 17219-1-AP). Following three washes with PBS, the sections were incubated with a horseradish peroxidase-conjugated secondary antibody (EnVision Detection System; ZSGB-BIO, Beijing, China; PV-6000) for 20 min at 37 °C. Immunoreactivity was visualized using 3,3′-diaminobenzidine (DAB) chromogen, and the sections were counterstained with haematoxylin, dehydrated, and mounted.

Digital images were captured using an OLYMPUS UC90 imaging system (Olympus Corporation, Tokyo, Japan) and quantitatively analysed using Image-Pro Plus software (Media Cybernetics Inc., Rockville, MD, USA). Positive staining intensity and area were measured in a semiautomated manner by selecting regions of interest on the basis of immunoreactive signals. The mean optical density was calculated as the ratio of the integrated optical density to the total image area. For each sample, five randomly selected high-power fields (400× magnification; scale 50 μm) were analysed, and the mean value was used for statistical analysis. Positive immunostaining appeared brown–yellow, while the cell nuclei were counterstained blue. To ensure objective evaluation, the semi-quantitative IHC scoring was independently performed by two experienced pathologists who were completely blinded to the patients' clinical information and sample group allocations.

### Statistical analysis

Statistical analysis was performed using Statistical Product and Service Solutions (SPSS) 22.0. The quantified data are expressed as the mean ± standard deviation. Comparisons between groups were performed with independent-sample *t* tests. Analysis of variance (ANOVA) was used to compare the means of more than two groups. *P* values of <0.05 were considered significant.

## Results

### Mendelian randomization analysis of mitochondria-related gene eQTLs

TSMR analysis was performed on 861 mitochondria-related genes, revealing causal relationships between 105 mitochondrial gene eQTLs and EMs. Subsequent heterogeneity and horizontal pleiotropy tests were conducted for positive exposures. The results demonstrated that BLOC1S1 (IVW *P* = 0.0056; OR = 1.39), DNA2 (IVW *P* = 0.015; OR = 1.16), and NARS2 (IVW *P* = 3.77 × 10^−5^; OR = 1.10) promote EMs development, whereas FIS1 (IVW *P* = 0.00055; OR = 0.92), IMMT (IVW *P* = 6.45 × 10^−7^; OR = 0.91), and MRPL55 (IVW *P* = 0.00033; OR = 0.79) confer protective effects against EMs.

Heterogeneity testing indicated no significant heterogeneity among the SNPs of FIS1, IMMT, MRPL55, BLOC1S1, DNA2, MRPL21, and NARS2 for EMs (*P* > 0.05). Assessment of pleiotropy (*P* value >0.05) demonstrated that these SNPs influence the occurrence of EMs through the aforementioned genes (Supplementary Table 1).

**Figure 2:**
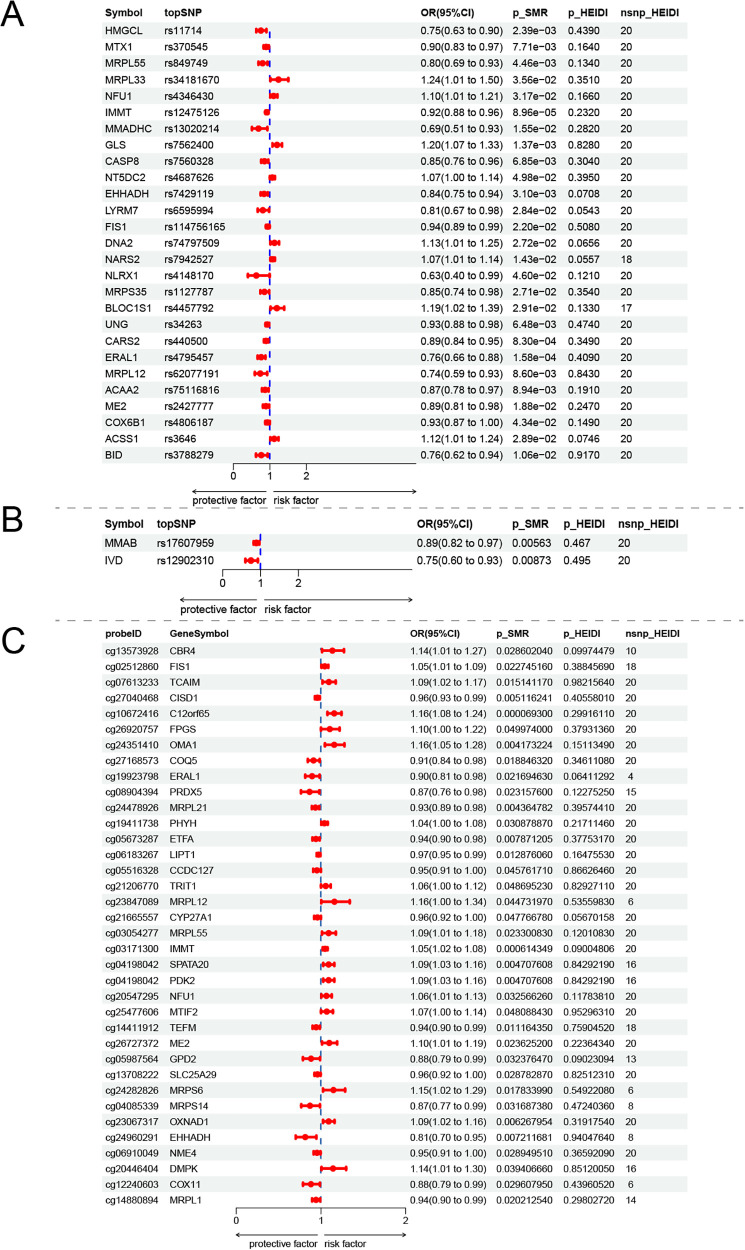
**(A)** Forest plot of the effects of cis-eQTLs on EMs according to Mendelian randomization analyses. **(B)** Forest plot of the effects of cis-pQTLs on EMs according to Mendelian randomization analyses. **(C)** Forest plot of the effects of cis-mQTLs on EMs according to Mendelian randomization analyses.

SMR analysis revealed 866 mitochondrial gene eQTLs associated with EMs, of which 59 demonstrated causal relationships (P_SMR < 0.05, P_HEIDI > 0.05) (Supplementary Table 2). Taking the intersection of 105 positive exposures from the TSMR and 59 from the SMR analysis yielded 27 mitochondrial gene eQTLs, which were visualized in forest plots (Figure 2A). The results further demonstrated that BLOC1S1, DNA2, and NARS2 promote EMs, whereas FIS1, IMMT, and MRPL55 protect against the development of EMs. The MR Steiger directionality test confirmed the absence of reverse causality at the eQTL level. Furthermore, the leave-one-out analysis demonstrated that our results were not driven by any idiosyncratic eQTL SNP, confirming the high stability of the causal estimates (Supplementary documents1).

### Mendelian randomization analysis of mitochondria-related gene pQTLs

TSMR analysis of 89 mitochondria-related genes revealed causal relationships between 4 mitochondrial gene pQTLs and EMs, P6 (IVW *P* value = 0.045, OR = 0.96), CRYZ (IVW *P* value = 0.0017, OR = 0.90), IVD (IVW *P* value = 0.0082, OR = 0.79), and MMAB (IVW *P* value = 0.011, OR = 0.89), which demonstrated protective effects against EMs. Neither heterogeneity nor pleiotropy testing revealed significant effects (*P* > 0.05) (Supplementary Table 3).

SMR analysis revealed 113 mitochondrial gene pQTLs associated with EMs, of which 9 demonstrated causal relationships (P_SMR < 0.05, P_HEIDI > 0.05) (Supplementary Table 4). The intersection of the results of both methods yielded 2 mitochondrial gene pQTLs (MMAB and IVD), with forest plots confirming that their protective effects were consistent with the TSMR results (Figure 2B). Similar to the transcriptomic analysis, we rigorously assessed the robustness of the proteomic-level causal associations. The Steiger filtering applied to the pQTL data verified that there was no reverse causation from EMs to the identified protein targets. Additionally, the leave-one-out sensitivity analysis indicated that the sequential exclusion of any single pQTL SNP did not significantly alter the magnitude or direction of the overall causal effect, ensuring the reliability of our findings at the protein level (Supplementary Documents 2).

### Mendelian randomization analysis of mitochondria-related gene mQTLs

TSMR analysis of 412 mitochondria-related gene methylation site (CpG) mQTLs revealed causal relationships between 113 methylation sites and EMs (Supplementary Table 5). Among these genes, MRPS6 (IVW *P* value = 0.019; OR = 1.20), COQ5 (IVW *P* value = 0.046; OR = 1.05), and PDK2 (IVW *P* value = 0.043; OR = 1.05) promote EMs development, whereas GPD2 (IVW *P* value = 0.0026; OR = 0.89), OMA1 (IVW *P* value = 0.010; OR = 0.92), and MRPL55 (IVW *P* value = 0.0013; OR = 0.89) confer protective effects. Neither heterogeneity nor pleiotropy testing revealed statistical significance (*P* > 0.05) (Supplementary Table 6).

SMR analysis yielded 819 methylation sites, 319 of which demonstrated causal relationships. Taking the intersection yielded 113 methylation sites, whose results were consistent with those of the TSMR analysis (Figure 2C). For the epigenetic MR analysis, the MR Steiger directionality test successfully ruled out potential reverse causality at the mQTL level. The leave-one-out analysis further corroborated these results, showing that the causal estimates between DNA methylation targets and EMs remained robust and were completely independent of any specific mQTL instrumental variable (Supplementary documents 3).

### Integration of multiomics-level evidence

Integration of mQTL, eQTL, and pQTL analysis results with EMs revealed a total of 128 mitochondria-related genes with causal relationships to EMs (Supplementary Table 7).

### PPI network construction and functional enrichment analysis

**Figure 3:**
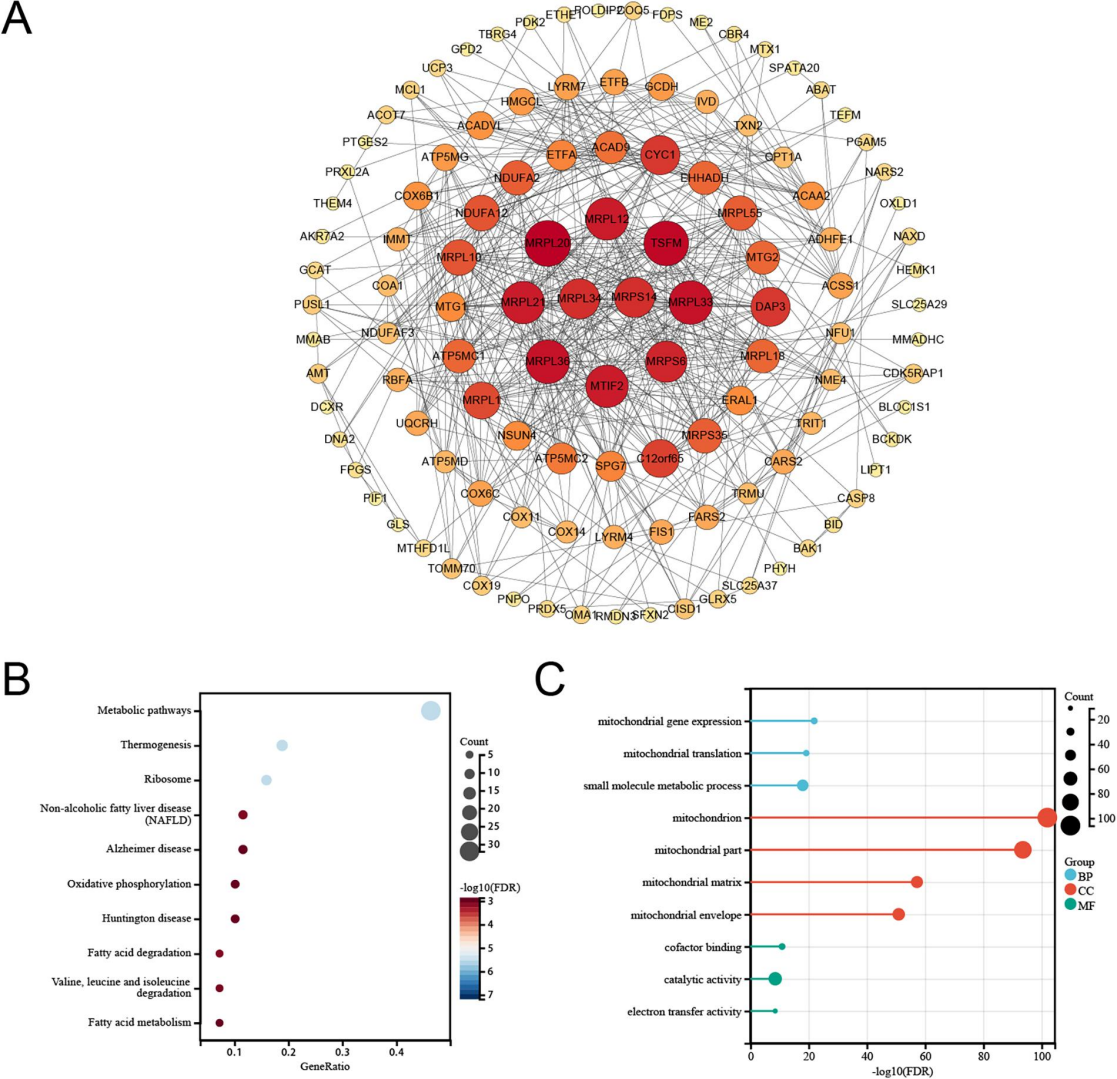
Interaction network and functional enrichment of causal genes. **(A)** Protein–protein interaction network; redder nodes indicate greater degrees (more interactions). **(B)** KEGG pathway enrichment bubble plot. **(C)** GO term enrichment bar plot.

A protein–protein interaction network was constructed on the basis of causally related genes, with MRPL12, MRPL20, MRPL21, and MRPL33 demonstrating extensive interaction relationships within the network (Figure 3A). KEGG pathway enrichment analysis revealed that the 128 genes were enriched primarily in the steroid biosynthesis and fatty acid elongation pathways (Figure 3B). GO analysis demonstrated enrichment in mitochondrial gene expression (biological process), mitochondria (cellular component), and oxidoreductase activity (molecular function) (Figure 3C).

### Differential gene expression screening

**Figure 4:**
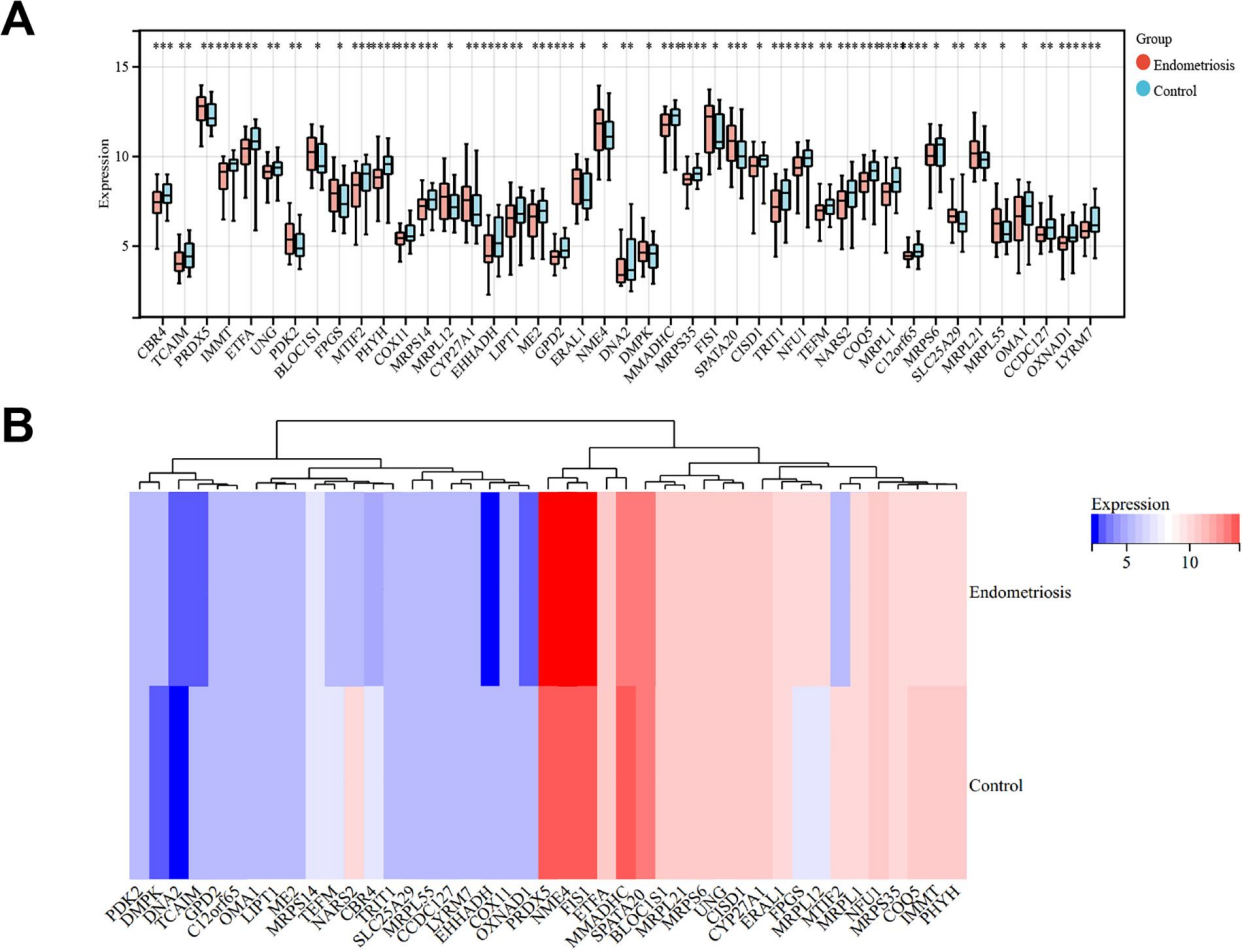
Selection of differentially expressed genes (DEGs). **(A)** Boxplot of DEGs between cases and controls. **(B)** Heatmap of DEGs between cases and controls. *P < 0.05, **P < 0.01, ***P < 0.001, ****P < 0.0001.

A rank-sum differential analysis of 128 key genes between the case and control samples yielded 42 differentially expressed genes, with 14 upregulated and 28 downregulated genes (Figure 4).

### Machine learning-based feature gene selection

**Figure 5:**
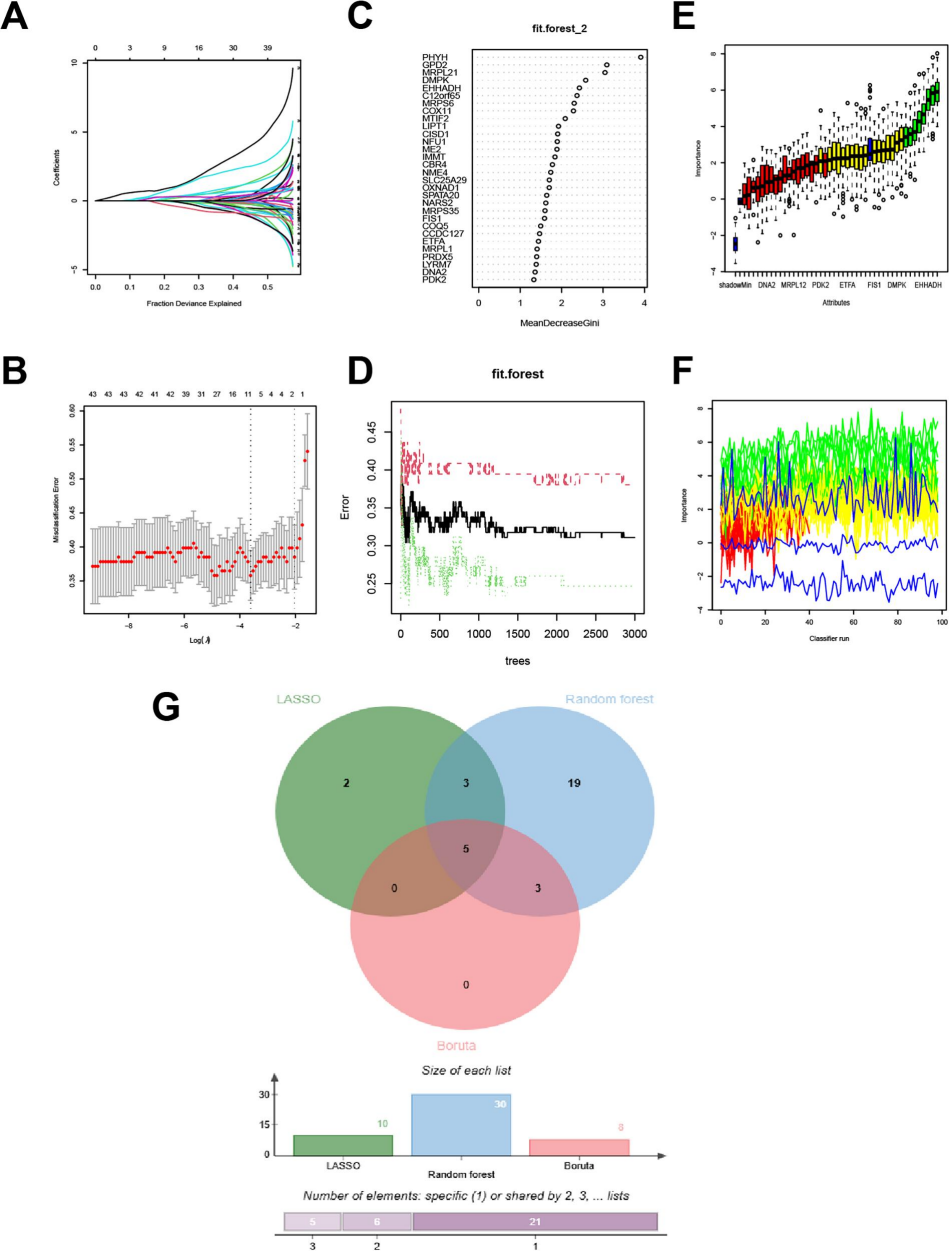
Selection of signature genes. **(A)** Profiles of LASSO coefficients. **(B)** Cross-validated deviance across the LASSO penalty path; the two vertical dashed lines indicate lambda.min (left) and lambda.1se (right). **(C)** Relative importance of genes from the random forest. **(D)** Error rate as a function of the number of trees in the random forest. **(E)** Boruta importance boxplots for each gene. **(F)** Line plot of the Boruta importance distribution. **(G)** Venn diagram showing the intersection of genes selected by the three machine learning algorithms.

Three machine learning algorithms were employed for feature selection: LASSO regression retained 10 genes (Figures 5A,B), random forest identified 30 genes with importance values greater than 4 (Figures 5C,D), and the Boruta algorithm confirmed 7 genes (Figures 5E,F). The intersection of all three methods yielded 5 feature genes: PHYH, GPD2, C12orf65, MRPS6, and RPL21 (Figure 5G).

### Feature gene validation and performance assessment

**Figure 6:**
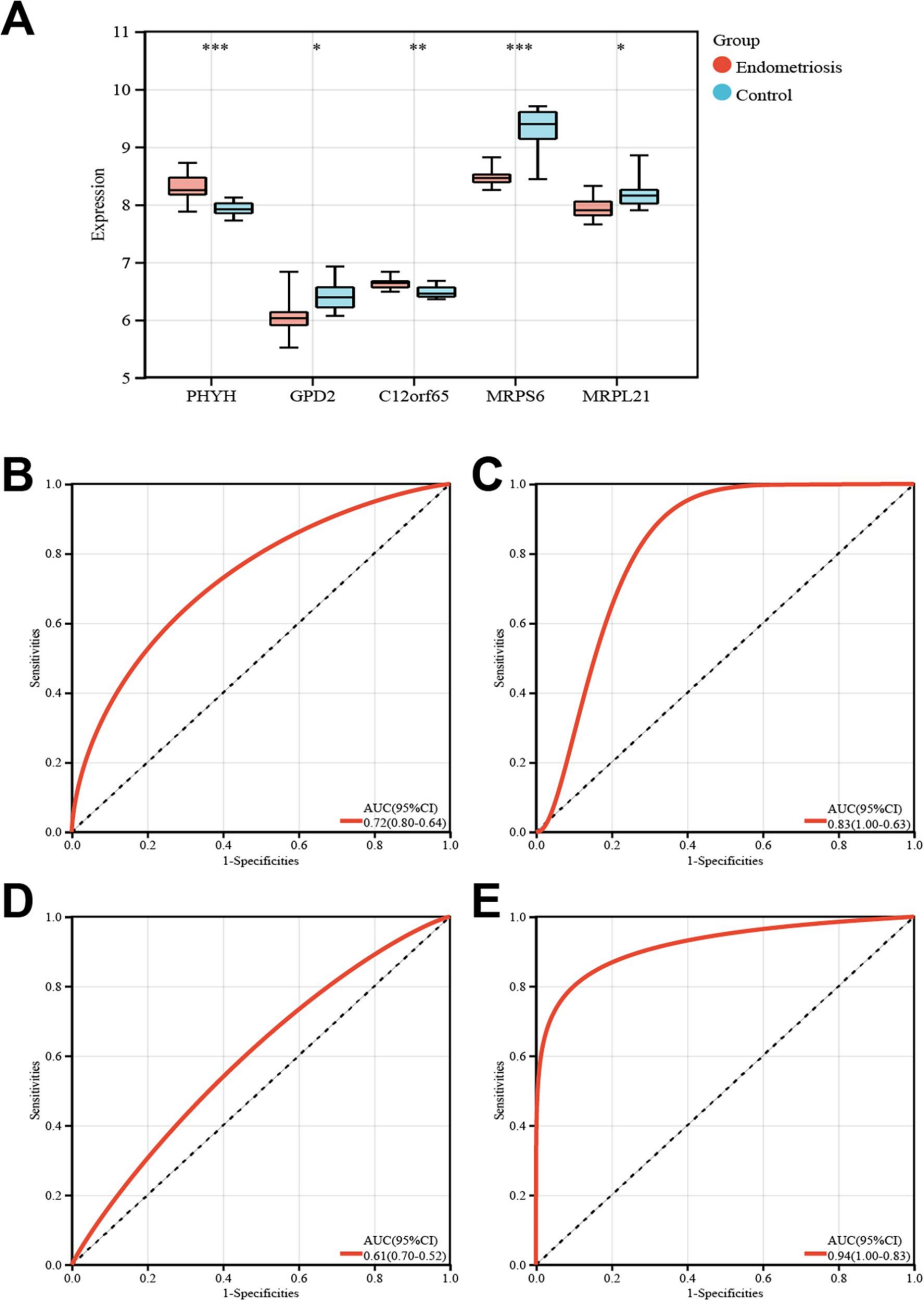
Validation of signature genes. **(A)** Boxplot of DEGs between cases and controls in the validation set. **(B)** ROC curve for GPD2 in the training set. **(C)** ROC curve for GPD2 in the validation set. **(D)** ROC curve for MRPS6 in the training set. **(E)** ROC curve for MRPS6 in the validation set.

Validation of the differential expression of feature genes in the training and validation sets revealed that GPD2 and MRPS6 were significantly differentially expressed, with consistent trends across both datasets (Figure 6A). ROC curve analysis indicated that the diagnostic models constructed with these two biomarkers achieved moderate diagnostic accuracy in both the training and validation sets. Specifically, GPD2 yielded an Area Under the Curve (AUC) of 0.72 (95% CI: 0.64–0.80) in the training set (Figure 6B) and 0.83 (95% CI: 0.63–1.00) in the validation set (Figure 6C). Similarly, MRPS6 demonstrated an AUC of 0.61 (95% CI: 0.52–0.70) in the training set (Figure 6D) and 0.94 (95% CI: 0.83–1.00) in the validation set (Figure 6E). These findings identify GPD2 and MRPS6 as promising exploratory candidates for further diagnostic development. Therefore, GPD2 and MRPS6 promising exploratory candidates for EMs and may be associated with disease-related metabolic abnormalities.

### Analysis of the associations between feature mitochondrial genes and immune status

**Figure 7:**
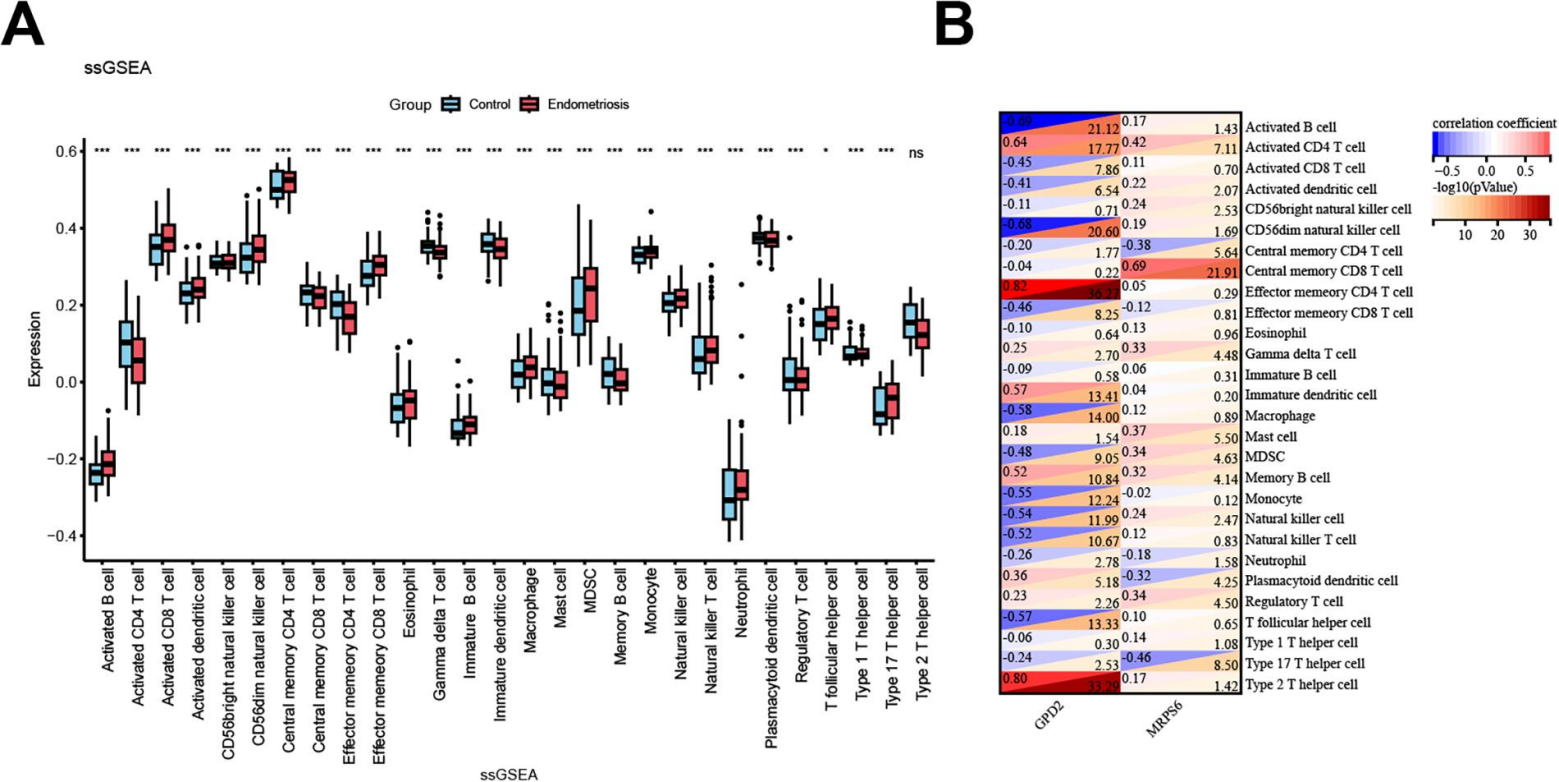
Associations between signature genes and immune cells. **(A)** Boxplots of immune cell infiltration. **(B)** Heatmap of correlations between GPD2/MRPS6 and immune cells. *P < 0.05, **P < 0.01, ***P < 0.001, ****P < 0.0001.

Single-sample gene set enrichment analysis (ssGSEA) was employed to evaluate immune cell type enrichment scores, revealing significant differences in immune infiltration between endometriotic and normal endometrium (Figure 7A). The expression of GPD2 and MRPS6 was significantly correlated with the infiltration of various immune cells, including macrophages and NK cells (Figure 7B), suggesting that mitochondrial genes may influence EMs progression through immune microenvironment regulation, with mitochondrial–immune interactions representing potential therapeutic targets.

### Small-molecule drug prediction analysis

**Figure 8:**
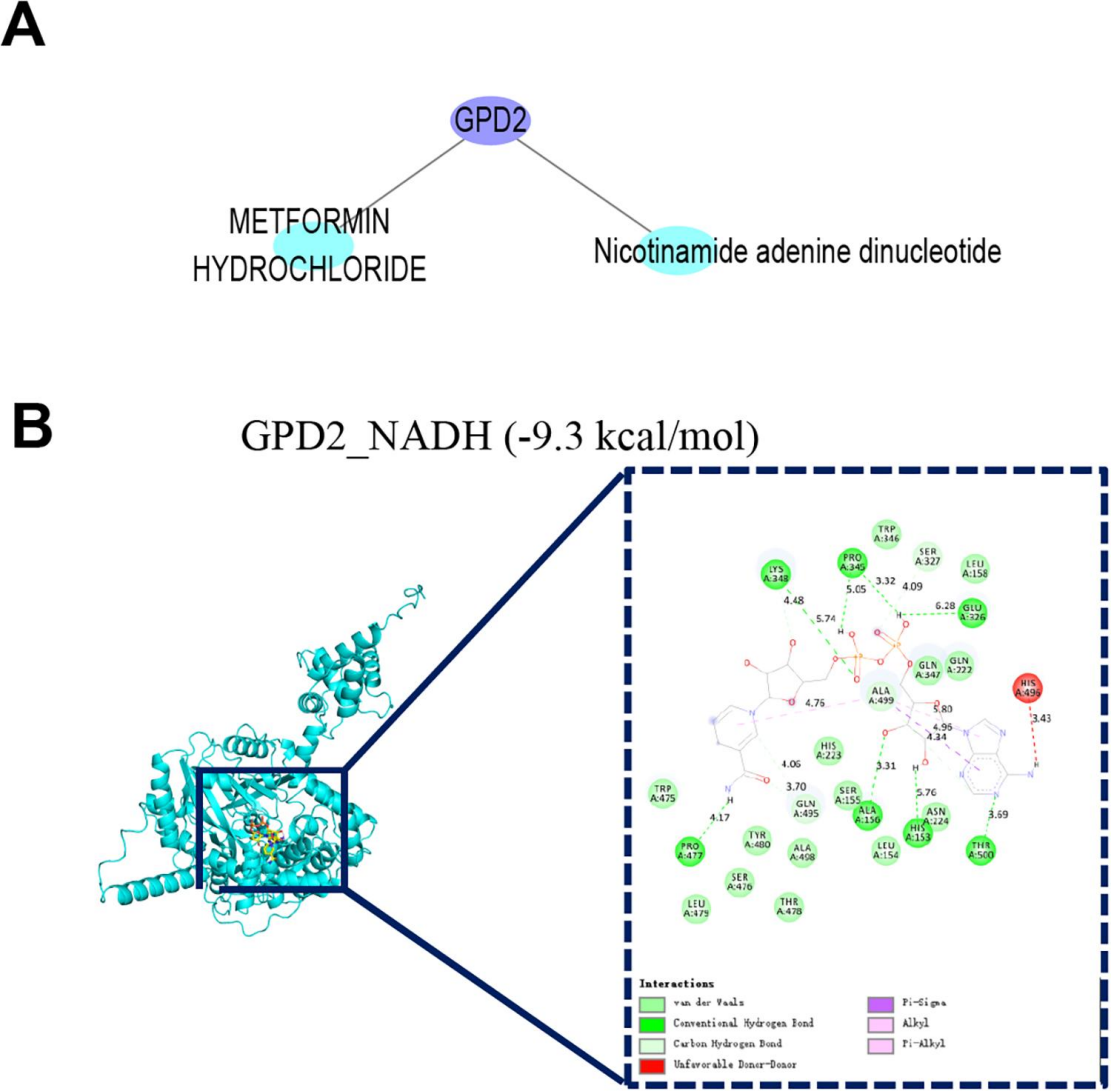
Drug prediction. **(A)** Network of predicted small-molecule–gene interactions (purple represents genes and blue represents drugs). **(B)** Molecular docking.

A network was constructed on the basis of gene–drug interactions (Figure 8A). Only GPD2 demonstrated predicted drug regulatory results, including two compounds: metformin hydrochloride and nicotinamide adenine dinucleotide.

### Molecular docking analysis

Molecular docking analysis was performed between GPD2 and NADH. The results revealed a binding energy of −9.3 kcal/mol between the protein and small molecule, indicating a spontaneous binding capability between the target and NADH (Figure 8B).

### Molecular dynamics simulation confirms the stability of the GPD2-NADH complex

**Figure 9:**
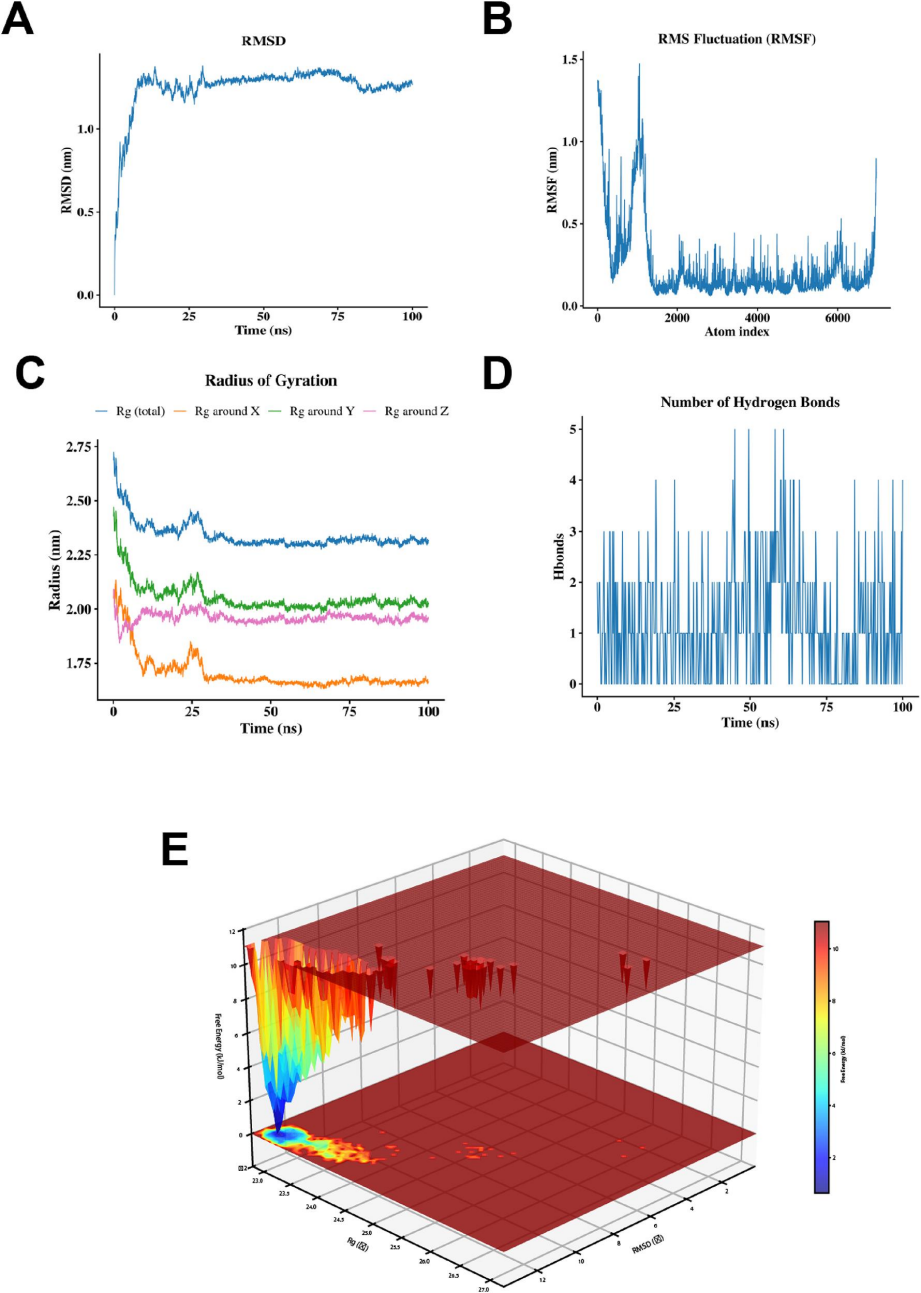
Molecular dynamics simulation of the GPD2-NADH complex. **(A)** Root mean square deviation (RMSD). **(B)** Root mean square fluctuation (RMSF) of GPD2. **(C)** Radius of gyration (Rg) of GPD2. **(D)** Dynamic changes in the number of hydrogen bonds for GPD2. **(E)** Free energy landscape (FEL) of GPD2.

The 100-ns MD simulation of the GPD2-NADH complex revealed that the system's RMSD stabilized rapidly after an initial relaxation phase. The overall fluctuation range remained narrow with no drastic changes, exhibiting only a mild oscillation around 75 ns. This indicates that the complex structure rapidly reached thermodynamic equilibrium and maintained stable binding (Figure 9A). The RMSF analysis demonstrated that the core secondary structures of the protein exhibited low flexibility and stable conformations, whereas regions of high fluctuation were primarily concentrated in loop regions and disordered terminal segments, reflecting a dynamic characteristic of overall stability coexisting with local flexibility (Figure 9B).

Furthermore, the Rg results showed that after initial conformational compaction, the system consistently maintained an Rg value of approximately 2.3 nm throughout the simulation. The components along each axis displayed consistent trends without structural expansion or dissociation, confirming that the complex remained highly compact and spatially stable in the solvated environment (Figure 9C). Hydrogen bond analysis revealed dynamic fluctuations in the number of hydrogen bonds between the protein and the ligand (predominantly ranging from 1 to 3). Despite their transient nature, these interactions formed a continuously effective non-covalent interaction network, providing robust support for binding specificity (Figure 9D).

Finally, the FEL presented a single, deep global minimum energy basin, with conformations highly enriched in the low-energy region. This suggests that NADH induces GPD2 to converge into a stable, compact dominant conformation, indicating excellent conformational matching and thermodynamic binding stability (Figure 9E). Collectively, these metrics confirm that the GPD2-NADH complex exhibits robust structural stability and firm binding during dynamic simulation, establishing a reliable basis for their interaction.

### Single-cell analysis

**Figure 10:**
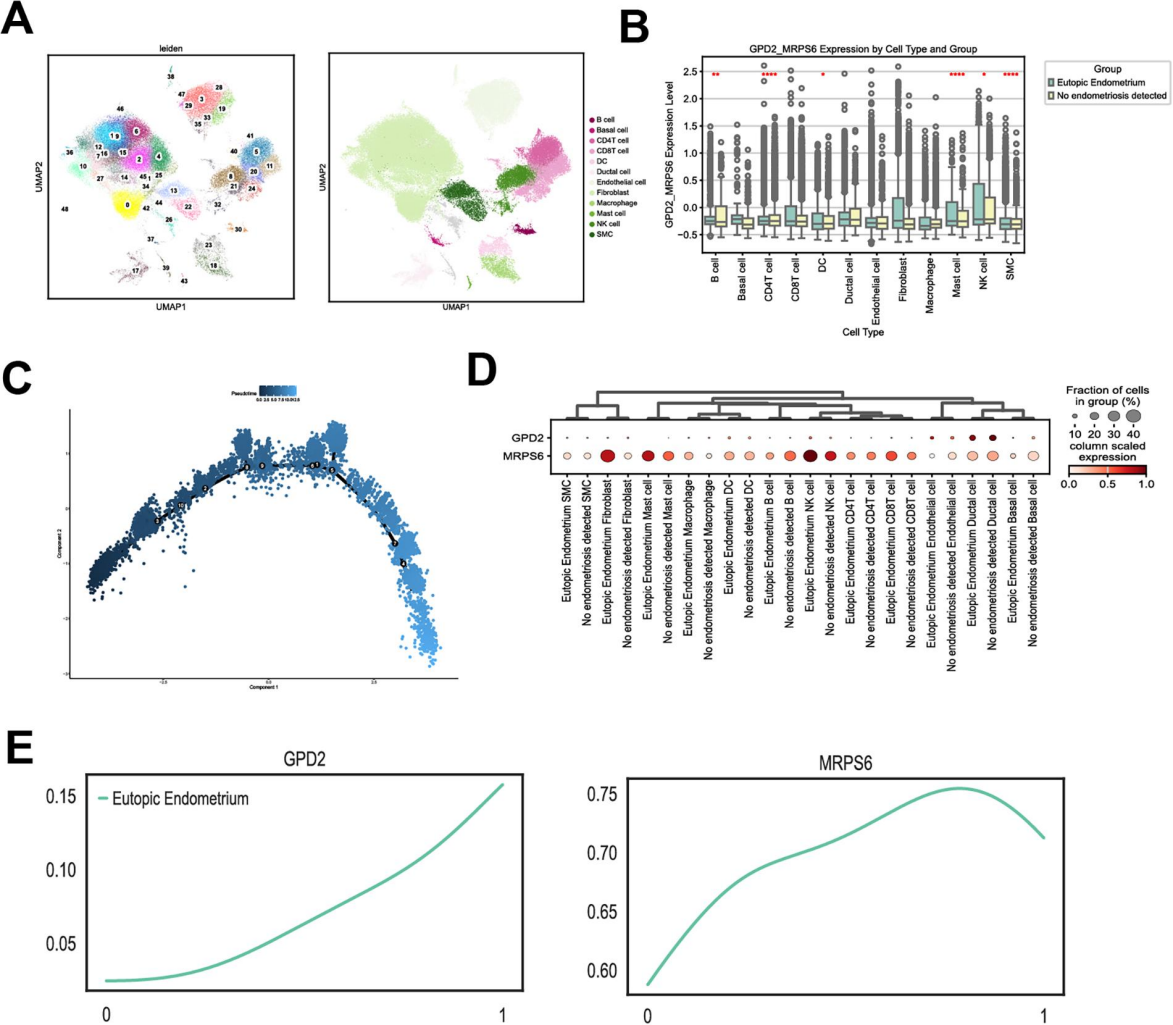
Single-cell analysis. **(A)** UMAP-based cell clustering and cell-type annotations. **(B)** Distribution of cell scores across 12 cell populations. **(C)** Developmental trajectory coloured according to pseudotime. **(D)** Bubble plot of GPD2 and MRPS6 expression across cell types in cases and controls. **(E)** Pseudotime expression curves of GPD2 and MRPS6.

Cell clustering and annotation: The uniform manifold approximation and projection (UMAP) dimensionality reduction algorithm was used to cluster cells, with SingleR annotation identifying 12 cell types: B cells, basal cells, CD4+ T cells, CD8+ T cells, dendritic cells, ductal cells, endothelial cells, fibroepithelial cells, macrophages, mast cells, NK cells, and smooth muscle cells (Figure 10A).

Cell score calculation: Cell scores were calculated for 12 cell populations on the basis of GPD2 and MRPS6 gene expression, with NK cells demonstrating the highest scores (Figure 10B).

Pseudotime analysis: NK cell data were extracted for Monocle analysis to construct cell developmental trajectory maps (Figure 10C).

Expression patterns of feature genes in single-cell data: Analysis of GPD2 and MRPS6 expression across different cell types revealed high expression in NK cells (Figure 10D). Pseudotime analysis demonstrated correlations between NK cell developmental states and gene expression dynamics (Figure 8E).

### Immunohistochemical validation of exploratory biomarkers

**Figure 11:**
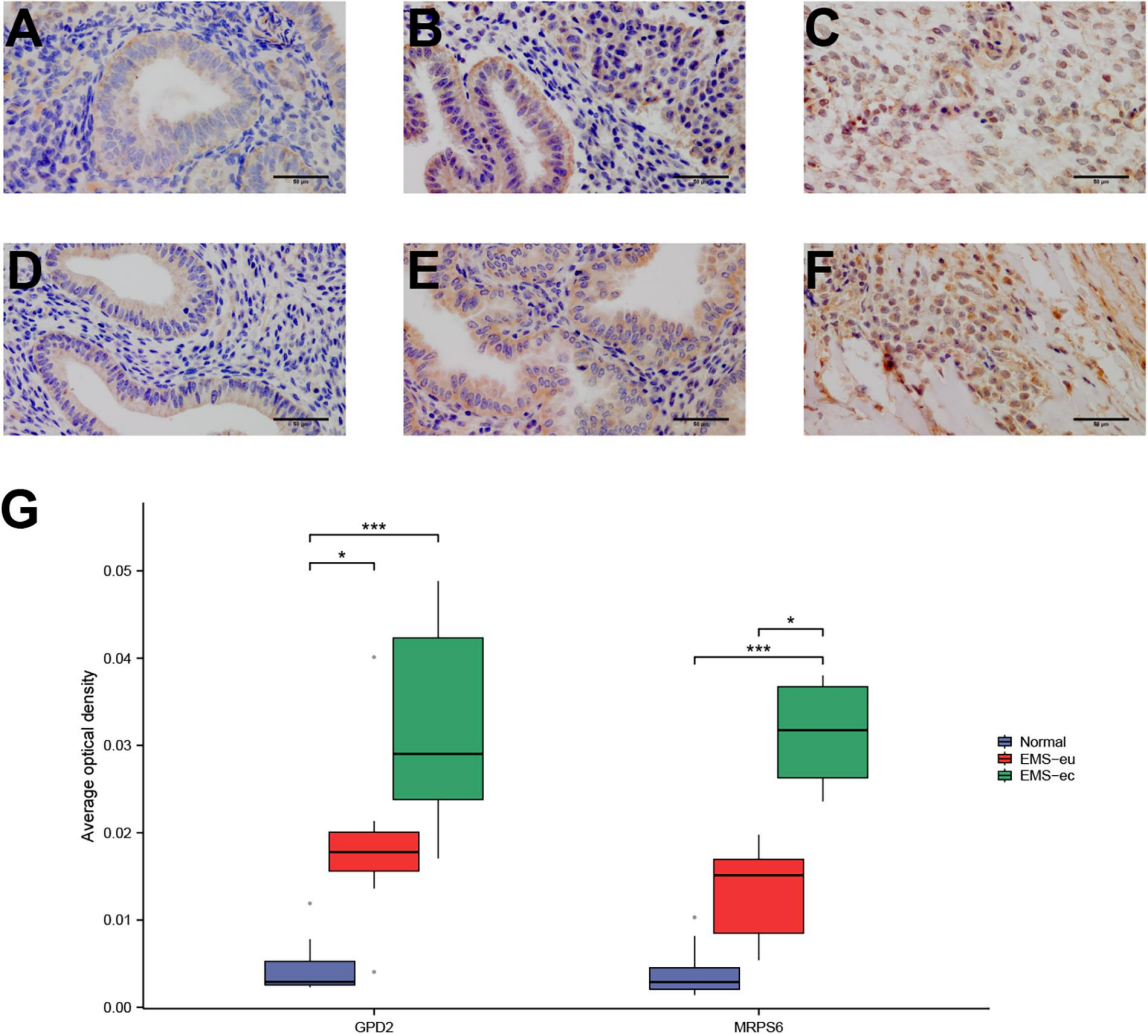
Expression of biomarkers in endometrial tissue sections. **(A–C)** GPD2 protein expression: **(A)** normal endometrium, **(B)** eutopic endometrium from EMs (EMs-eu), **(C)** ectopic endometrium from EMs (EMs-ec). **(D–F)** MRPS6 protein expression: **(D)** normal endometrium, **(E)** eutopic endometrium from Ems (EMs-eu), **(F)** ectopic endometrium from Ems (EMs-ec). **(G)** Statistical analysis of the mean optical density for biomarker protein expression in tissue sections. Magnification: 400×. *P < 0.05, **P < 0.01, ***P < 0.001.

Immunohistochemical analysis revealed differential staining patterns for GPD2 and MRPS6 across tissue types, with the most intense staining observed in ectopic endometria from EMs patients and the weakest staining in normal endometria (Figures 11A–F). Semiquantitative analysis based on the mean optical density demonstrated that compared with those in normal endometria, GPD2 expression levels in ectopic endometria from EMs patients were significantly elevated. Similarly, MRPS6 was strongly expressed in ectopic endometria from EMs patients, with expression levels significantly greater than those observed in both eutopic endometria from EMs patients and normal endometria (Figure 11G). These findings were consistent with the transcriptomic data.

## Discussion

Our comprehensive Mendelian randomization analysis, incorporating genomic (eQTL), epigenomic (mQTL), and proteomic (pQTL) evidence, revealed 128 mitochondrial genes with significant causal relationships to EMs. This multilevel molecular evidence strengthens the reliability of causal inference by reducing the potential confounding factors inherent in traditional observational studies. These genes were significantly enriched in critical metabolic pathways, including those involved in steroid biosynthesis, fatty acid elongation, oxidative phosphorylation, and fatty acid degradation. The enrichment in steroid biosynthesis pathways is particularly noteworthy, as abnormal steroid hormone metabolism, especially oestrogen and progesterone regulation, represents a fundamental mechanism in EMs pathogenesis (42). Dysregulated steroid synthesis may contribute to the hormonal imbalance that promotes ectopic endometrial tissue growth and maintenance.

The enrichment in fatty acid metabolism pathways highlights the metabolic reprogramming that occurs in EMs. Fatty acid degradation and oxidative phosphorylation are essential for cellular energy homeostasis, and their disruption may impair cellular function and promote inflammatory responses (43). Importantly, AMPK and PI3K/AKT/mTOR signalling pathways may play pivotal roles in this process. Studies have demonstrated that aberrant fatty acid oxidation can activate the NLRP3 inflammasome through the AMPK/ACC/CPT1A pathway, leading to proinflammatory cytokine release and the initiation of inflammatory cascades (44). Similarly, research in rheumatoid arthritis has revealed that CD36-mediated fatty acid metabolic reprogramming regulates cellular proliferation, migration, and invasive capacity through the AMPK and PI3K/AKT/mTOR signalling pathways while also modulating proinflammatory cytokine secretion (45). Additionally, the involvement of valine, leucine, and isoleucine degradation pathways indicates altered branched-chain amino acid metabolism, which has been linked to metabolic dysfunction and inflammatory conditions. These metabolic alterations may create a microenvironment conducive to endometriotic lesion establishment and progression, providing energy substrates for abnormal cellular proliferation through dual regulation of the AMPK and PI3K/AKT/mTOR pathways while simultaneously triggering the inflammatory cascades that characterize this disease.

Through rigorous machine learning-based feature selection and experimental validation, we identified GPD2 and MRPS6 as robust exploratory biomarkers with consistent performance across multiple datasets. To the best of our knowledge, this is the first study to identify the causal role of GPD2 and MRPS6 in EMs. While direct literature linking these specific genes to EMs is currently absent, their biological functions align closely with the established metabolic features of the disease. Glycerol-3-phosphate dehydrogenase 2 (GPD2), a key enzyme in mitochondrial respiration and lipid metabolism, plays crucial roles in cellular energy homeostasis by facilitating the glycerol phosphate shuttle between the cytoplasm and mitochondria. In EMs, elevated GPD2 expression may reflect compensatory metabolic adaptations to meet the increased energy demands of rapidly proliferating ectopic endometrial cells. Since GPD2 can serve as a key link between metabolism, genetic instability and immunity, it holds significant importance in cancer research (46). The metabolic reprogramming of EMs may be similar to that of cancer cells. In this case, enhanced glycerol-3-phosphate metabolism can support the synthesis of substances and survival of cells under stress conditions (47, 48). The invasive characteristics of endometriotic lesions, including tissue penetration and angiogenesis, may similarly depend on GPD2-mediated metabolic flexibility.

Mitochondrial ribosomal protein S6 (MRPS6), an essential component of the mitochondrial ribosome, directly controls mitochondrial protein synthesis and respiratory complex assembly. Related studies on breast cancer have indicated that elevated expression levels of MRPS6 suggest enhanced mitochondrial biogenesis and protein synthesis capacity (49). In ectopic endometrium, increased MRPS6 expression may contribute to maintaining the high metabolic activity required for endometriotic lesions, reflecting cellular adaptation to oxidative stress and the inflammatory microenvironment characteristic. Both GPD2 and MRPS6 demonstrated moderate diagnostic performance (AUC > 0.6) and consistent expression patterns across independent cohorts, suggesting their potential as promising candidate biomarkers for the diagnosis of EMs. Notably, our clinical validation specifically targeted samples from patients with rASRM Stage IV EMs. Our results objectively demonstrate that GPD2 and MRPS6 are genetically associated with EMs and show significantly increased protein expression in advanced-stage disease. While we hypothesize that such metabolic reprogramming might be linked to the aggressive phenotype of severe EMs, we acknowledge that our current data primarily establish a genetic and observational association. Whether the robust upregulation of these mitochondrial genes actively drives tissue invasion, or merely reflects a secondary metabolic adaptation to the severe disease state, remains a hypothesis generated from these associations rather than a proven mechanism.

Regarding lesion heterogeneity, EMs encompasses distinct anatomical and pathological subtypes—primarily superficial peritoneal EMs (SUP), ovarian endometriomas (OMA), and deep infiltrating EMs (DIE)—each driven by potentially unique pathophysiological mechanisms. It is important to note that our findings are based on the FinnGen GWAS dataset, which aggregates these diverse subtypes into a broad EMs phenotype. While we acknowledge the inherent heterogeneity among these distinct subtypes, previous literature has demonstrated that key pathological genes often exhibit a consistently elevated expression trend across all EMs subtypes when compared to normal endometrium (50). Furthermore, despite anatomical differences, all endometriotic lesions share fundamental mechanistic drivers, including profound chronic inflammation and high metabolic demands (51). Consequently, the identified targets, GPD2 and MRPS6, likely represent a shared, constitutive metabolic vulnerability across the entire disease spectrum rather than subtype-specific drivers. While our clinical validation in severe Stage IV cases confirmed their robust upregulation in highly invasive phenotypes, the specific metabolic-immune coupling mechanisms might differ among SUP, OMA, and DIE. Therefore, future studies leveraging subtype-stratified GWAS datasets and well-defined clinical cohorts are essential to comprehensively map the spatiotemporal expression dynamics of these mitochondrial genes across specific lesion subtypes and menstrual cycle phases.

Our immune infiltration analysis revealed significant associations between mitochondrial genes and various immune cell populations, particularly macrophages and NK cells. It is important to note that these findings are based on transcriptomic correlations. Therefore, we propose a working hypothesis that the upregulation of GPD2 and MRPS6 might reflect a metabolic reprogramming event that subsequently modulates the immune microenvironment. Macrophages play dual roles in EMs pathogenesis: M1 macrophages promote proinflammatory responses and tissue damage, whereas M2 macrophages support anti-inflammatory, immunosuppressive, and tissue repair character (52). The functional status of mitochondria directly influences macrophage polarization, as M1 macrophages rely on glycolysis, whereas M2 macrophages depend on oxidative phosphorylation (53, 54). The association between mitochondrial genes and macrophage infiltration suggests that metabolic reprogramming may modulate the immune microenvironment in EMs, potentially shifting the balance towards a proinflammatory, M1-dominated state that perpetuates tissue damage and chronic inflammation. Furthermore, regarding the interpretation of the immune infiltration results, we acknowledge that the ssGSEA utilized standard, general-purpose immune signatures rather than endometrium-specific panels. Therefore, while these signatures provide valuable exploratory insights into the broad immune landscape of EMs, the specific functional states and exact proportions of these cells within the unique endometriotic microenvironment require further investigation using more highly specific, localized markers.

The expression of NK cells, critical components of innate immunity responsible for eliminating abnormal cells, was strongly correlated with GPD2 and MRPS6 expression in our single-cell analysis. Impaired NK cell function has been implicated in EMs pathogenesis, as reduced cytotoxic activity may allow ectopic endometrial cells to escape immune surveillance and establish persistent lesions (55). The significant correlation between these two mitochondrial genes and NK cells in our study highlights a tight connection between local metabolic alterations and the immune microenvironment in EMs. The precise biological roles of GPD2 and MRPS6 within NK cells, and how their expression profiles influence the local immune landscape, require further experimental investigation.

Our molecular docking and supplementary molecular dynamics (MD) simulations were positioned strictly as preliminary tools for target exploration. The strong and stable binding (binding energy: −9.3 kcal/mol) observed between GPD2 and its ubiquitous natural cofactor, NADH, provides structural evidence that the GPD2 catalytic pocket is accessible and potentially “druggable.” Rather than suggesting NADH as a novel therapeutic, these computational insights merely highlight the structural feasibility of targeting GPD2 to modulate mitochondrial metabolism. Furthermore, while our bioinformatic analysis hinted at a potential pathway overlap between *GPD2* and metformin, we emphasize the lack of direct binding evidence. Consequently, any clinical translational claims regarding specific metabolic modulators are premature and strictly necessitate future *in vitro* assays and *in vivo* EMs models for rigorous validation.

This study has several notable strengths that distinguish it from previous investigations. First and foremost, to the best of our knowledge, this is the pioneering study to systematically decode the causal role of mitochondrial genes in EMs using a multi-omics Mendelian randomization framework. Unlike traditional observational studies susceptible to confounding factors, our approach leverages genetic instruments to infer causality, providing robust targets for future intervention. Second, we identified GPD2 and MRPS6 as promising biomarkers that have not been previously reported in the context of EMs. This discovery fills a critical gap in our understanding of the metabolic-immune interface of the disease. Finally, a key innovation of our work lies in the integration of preliminary clinical validation. Despite the significant challenges in acquiring paired clinical tissues, we successfully performed initial immunohistochemical verification to ensure that our bioinformatics findings possess biological relevance.

Currently, the medical management of EMs predominantly relies on hormonal therapies, such as dienogest either alone or in combination with oral contraceptives. While these clinical interventions have proven highly effective in mitigating EMs-associated pain and improving patients’ quality of life (56, 57), they primarily offer symptomatic relief rather than targeting the fundamental disease mechanisms. Consequently, there is an increasing research focus on novel therapeutic strategies that directly modulate the inflammatory and oxidative stress pathways inherent to ectopic lesions. For instance, recent experimental studies have demonstrated the promising anti-inflammatory, antioxidant, and antiangiogenic efficacy of alternative agents, such as neroli oil, in endometriotic models (58). These evolving therapeutic trends strongly resonate with our findings. The identification of mitochondrial genes (*GPD2* and *MRPS6*) and their close correlation with the immune microenvironment highlights that targeting the mitochondrial-immune axis and alleviating metabolic/oxidative stress could be a crucial direction for developing the next generation of EMs treatments.

Despite these discoveries, our study has several limitations. First, due to the lack of large-scale, tissue-specific datasets, we utilized blood-derived eQTLs as proxies. Because EMs is a highly tissue-specific disorder shaped by a localized pelvic microenvironment, systemic blood eQTLs may not fully recapitulate local regulatory networks. Although our tissue-level IHC validation partially mitigates this gap, future studies leveraging endometrium-specific eQTL cohorts are essential. Second, while our analytical framework establishes robust genetic and observational associations, it does not confirm direct causal mechanisms. Based on current data, we cannot definitively determine whether the robust upregulation of *GPD2* and *MRPS6* actively drives disease progression or merely reflects a secondary metabolic adaptation to the severe disease state. Rigorous *in vitro* and *in vivo* functional assays are strictly required to elucidate their precise roles. Finally, our clinical validation was constrained by a small, single-center cohort comprising exclusively advanced (Stage IV) patients. This limits the generalizability of our findings to early-stage or mild EMs. Therefore, future multi-center, large-scale studies stratifying diverse disease stages and lesion subtypes are necessary to comprehensively evaluate the spatiotemporal expression dynamics of these mitochondrial targets.

In conclusion, by integrating multi-omics Mendelian randomization with transcriptomic and single-cell analyses, this study identifies *GPD2* and *MRPS6* as mitochondrial genes genetically associated with EMs. Our findings demonstrate that the expression patterns of these genes are significantly altered in endometriotic lesions and are closely correlated with specific immune microenvironment signatures, particularly NK cells, at the transcriptomic level. Rather than establishing definitive functional mechanisms, our study provides robust genetic and observational evidence highlighting a potential interplay between mitochondrial metabolic alterations and local immune responses. Future *in vitro* and *in vivo* functional studies are imperative to elucidate the precise biological roles of *GPD2* and *MRPS6* and to determine their exact mechanistic contributions to the pathogenesis of EMs.

## Data Availability

The original contributions presented in the study are included in the article/Supplementary Material, further inquiries can be directed to the corresponding author.
