# Supplementary figures and images for: Multi-omics Mendelian randomization identifies mitochondrial genes associated with immune microenvironment signatures in endometriosis
Source: Front Reprod Health. 2026 May 4;8:1747031. doi: 10.3389/frph.2026.1747031 (PMC13180925; doi:10.3389/frph.2026.1747031)

### ACP6 – Leave-One-Out MR

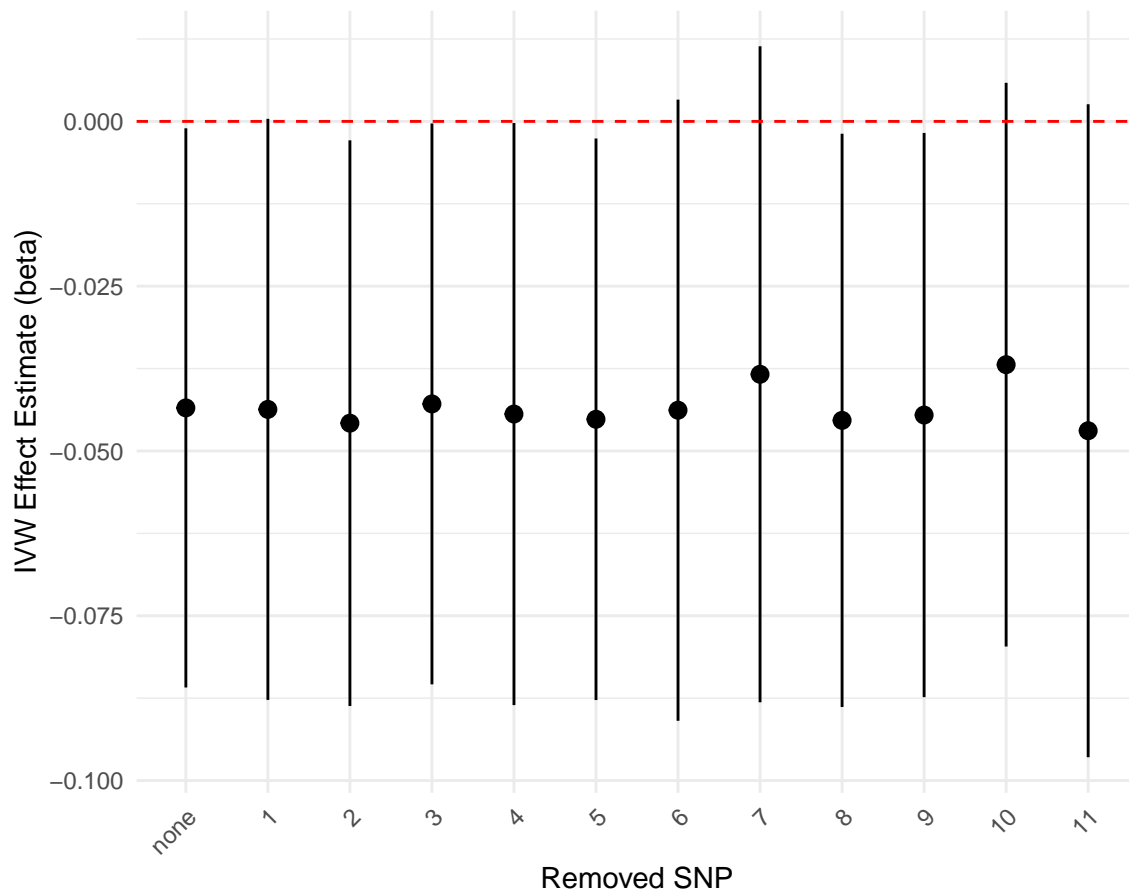

### ACP6 – Forest Plot

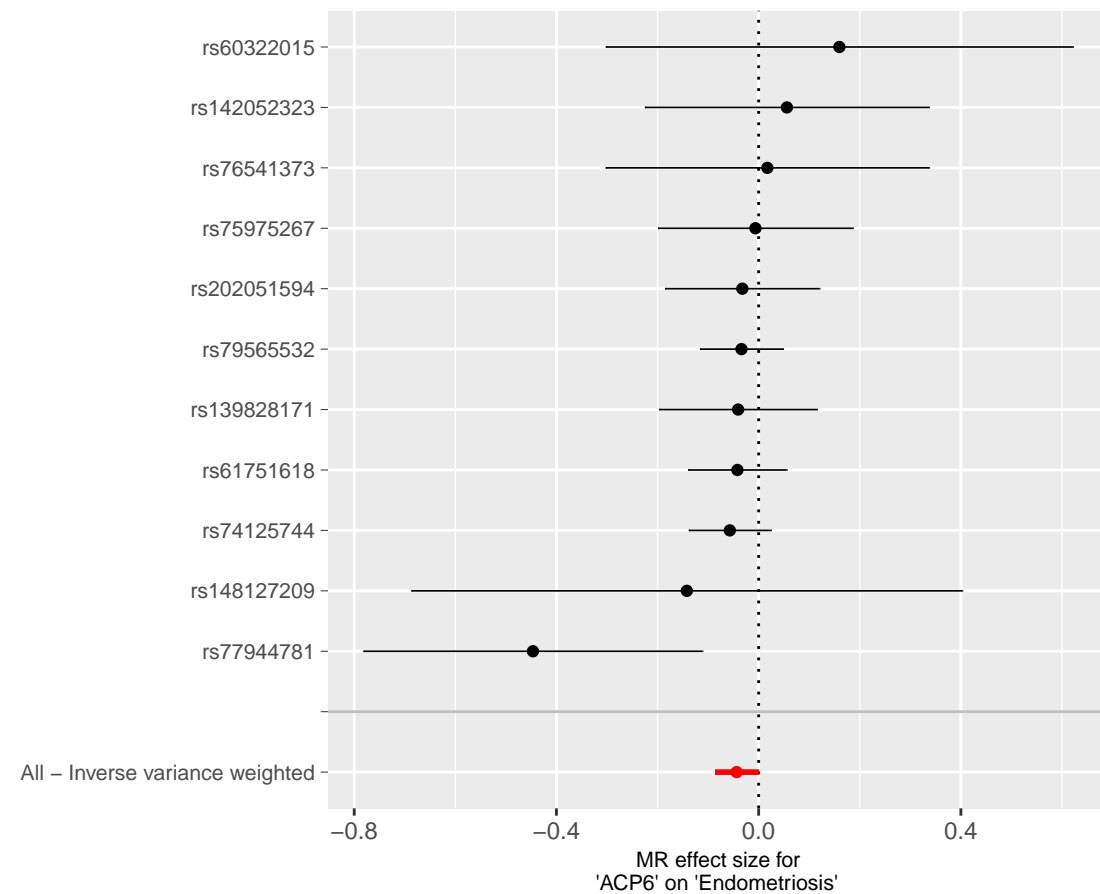

### ACP6 – Funnel Plot

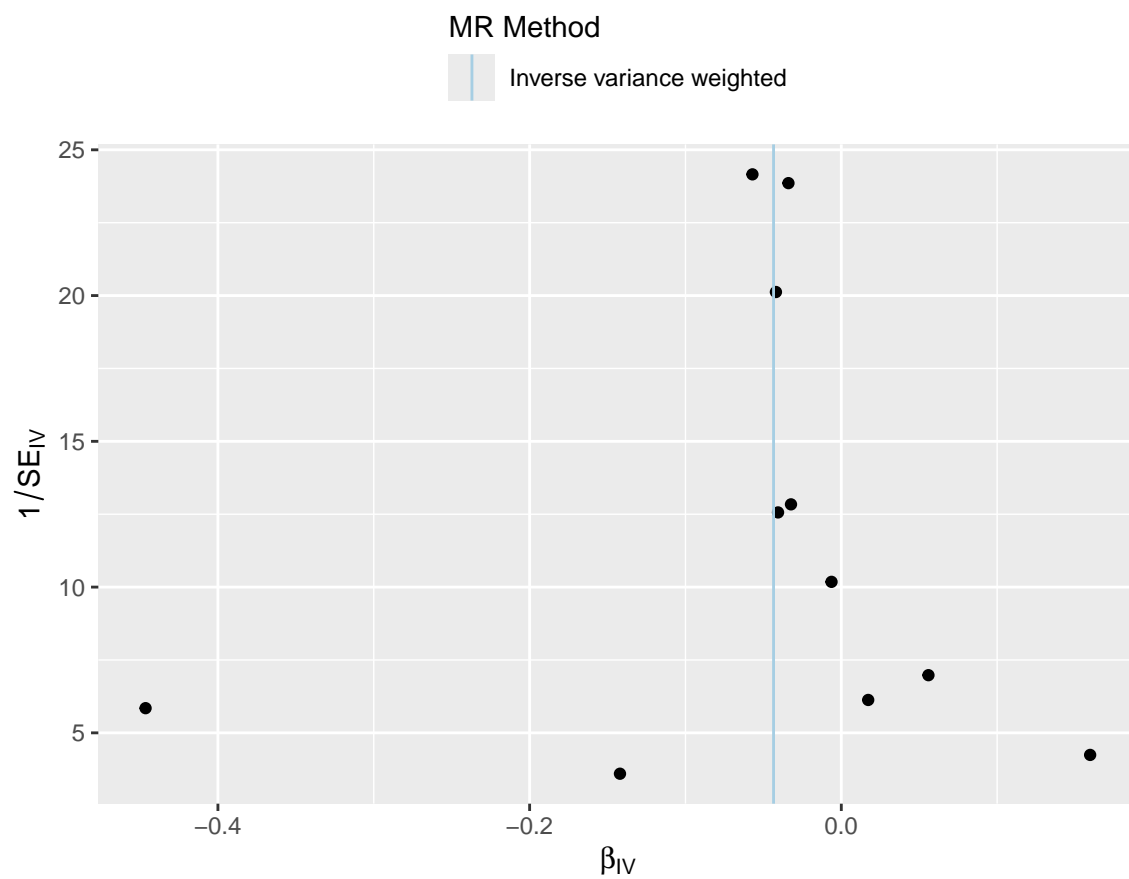

Supplement: Supplementary file 3 [file Datasheet3.zip › Supplementary documents2/pQTL_LOO/ACP6_LOO_MR.pdf]

CRYZ – Leave-One-Out MR

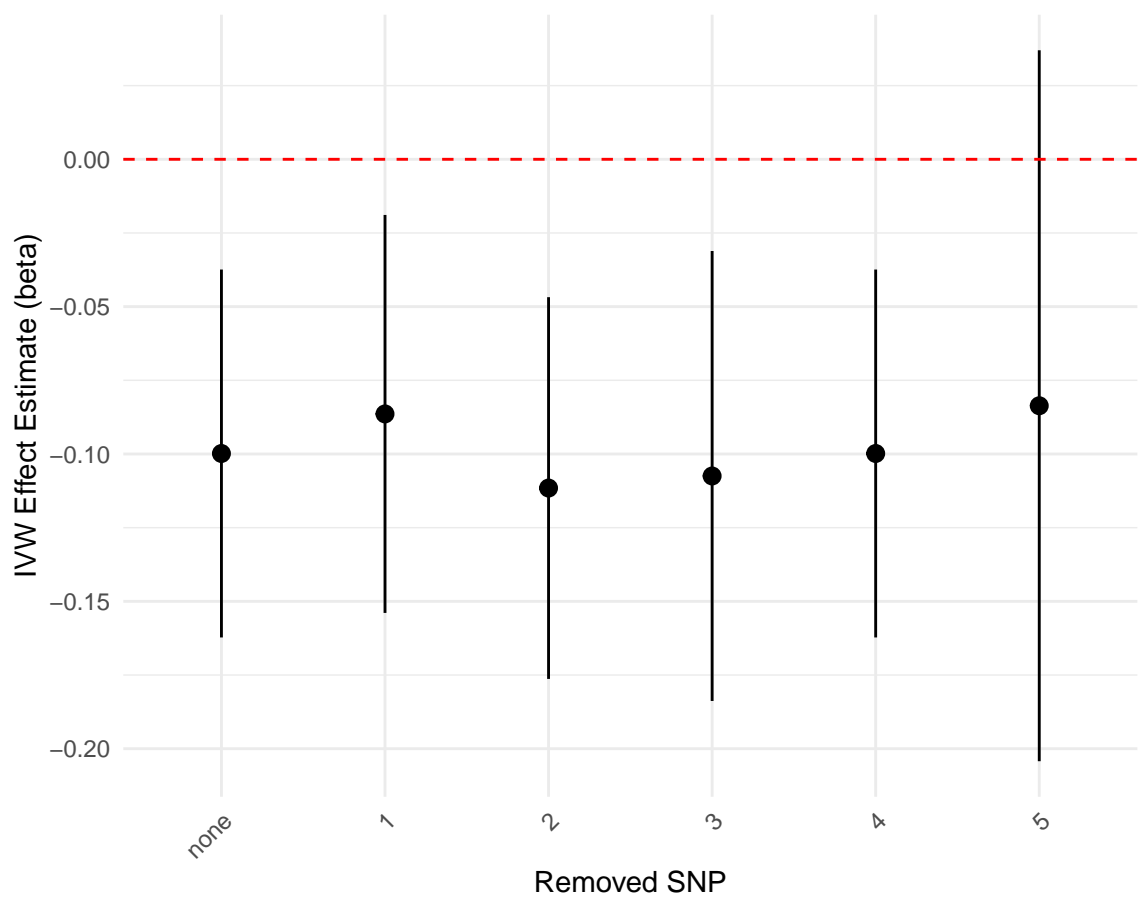

CRYZ – Forest Plot

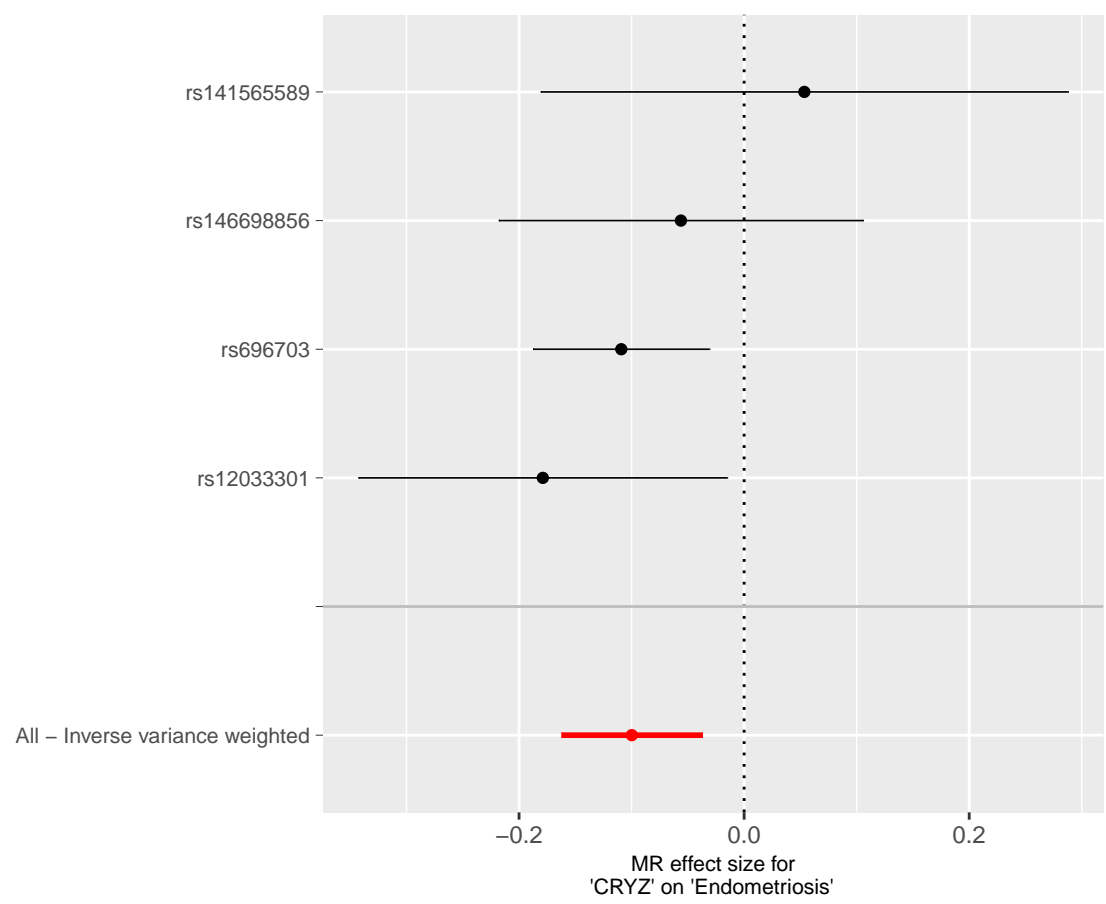

CRYZ – Funnel Plot

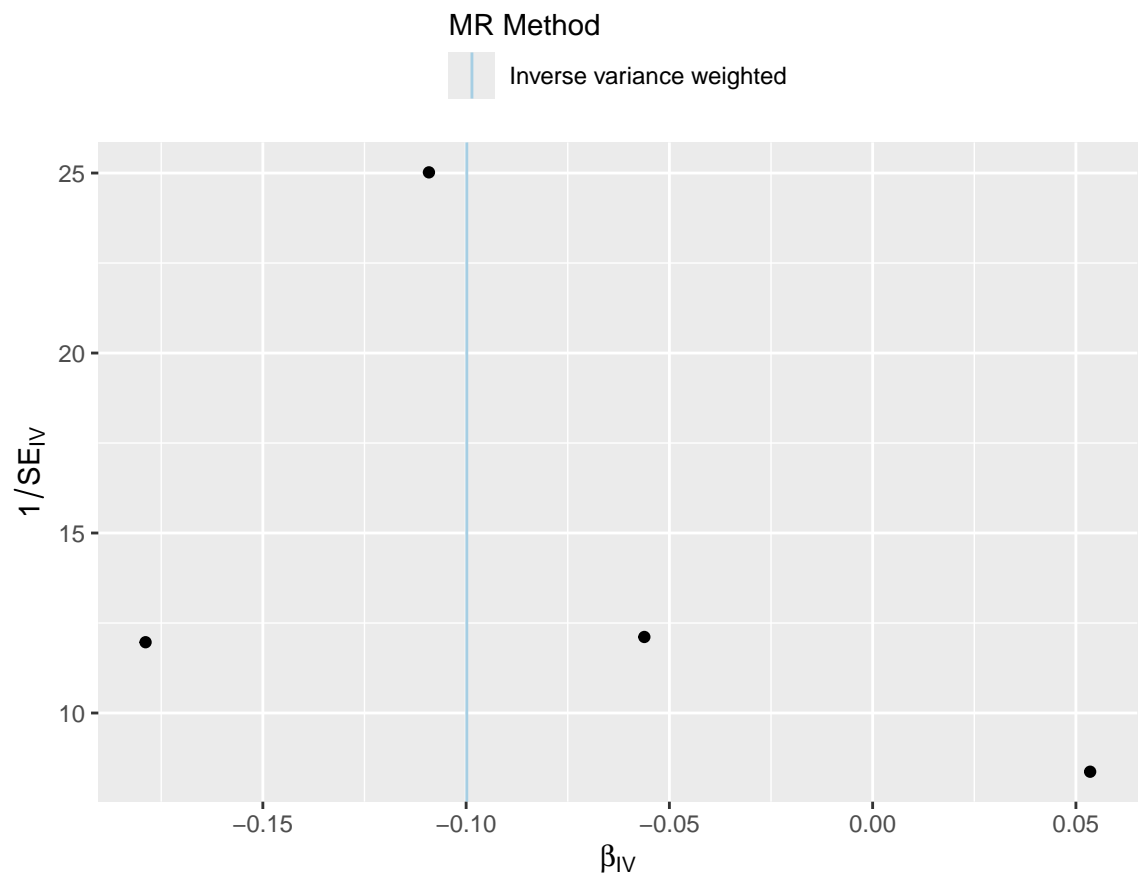

Supplement: Supplementary file 3 [file Datasheet3.zip › Supplementary documents2/pQTL_LOO/CRYZ_LOO_MR.pdf]

CBR4 – Leave-One-Out MR

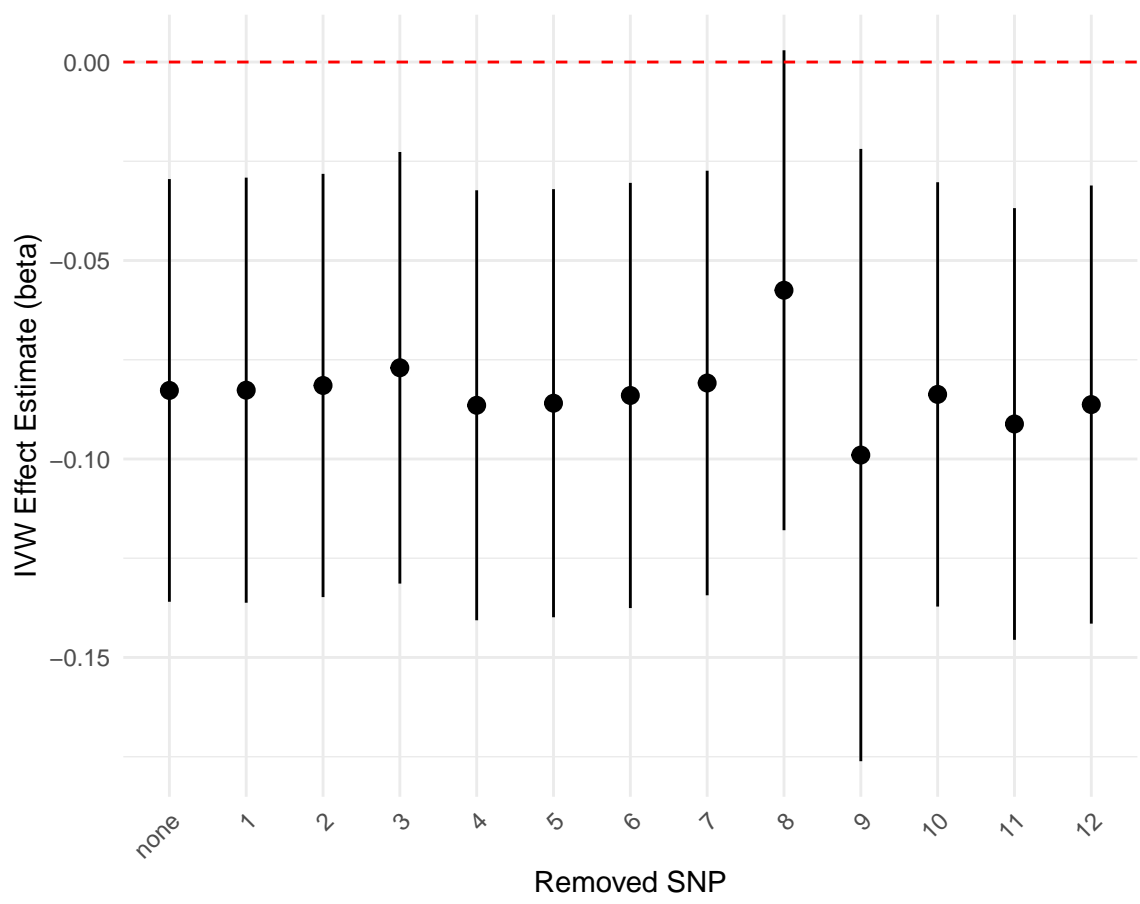

CBR4 – Forest Plot

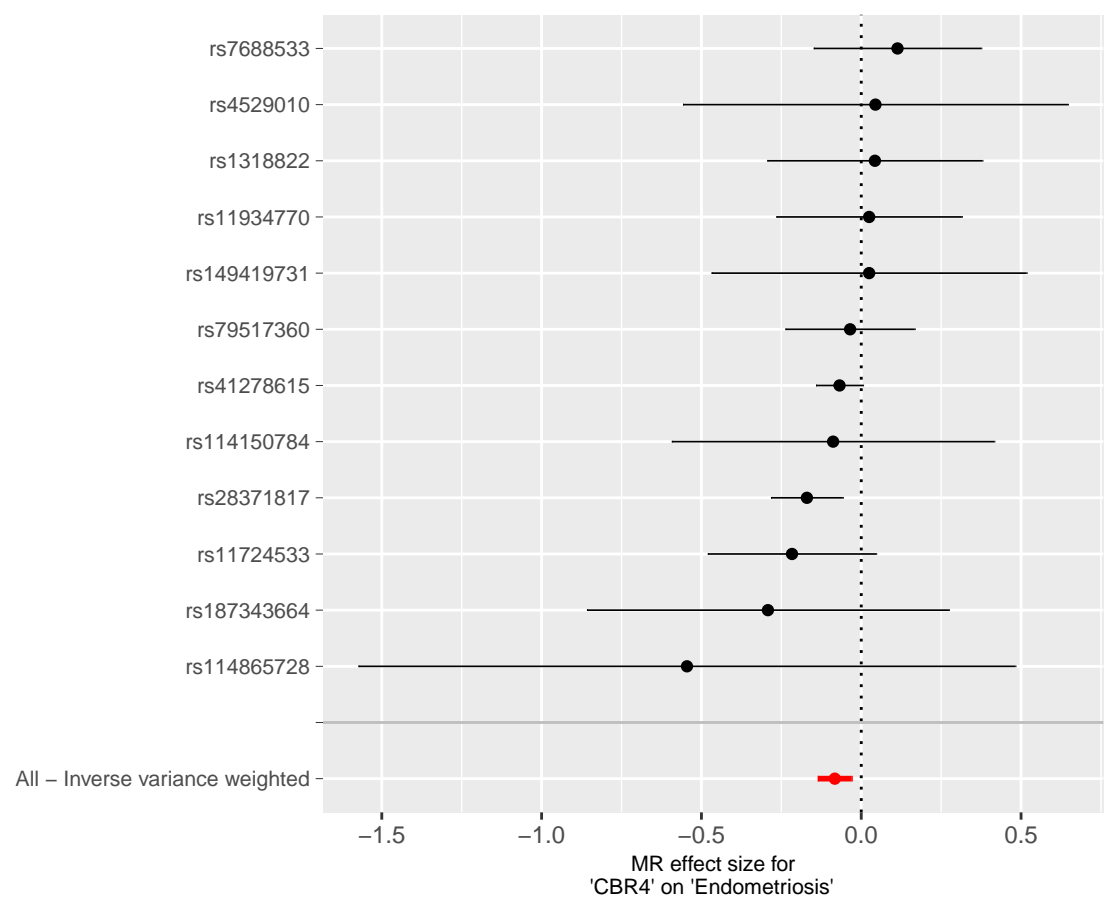

CBR4 – Funnel Plot

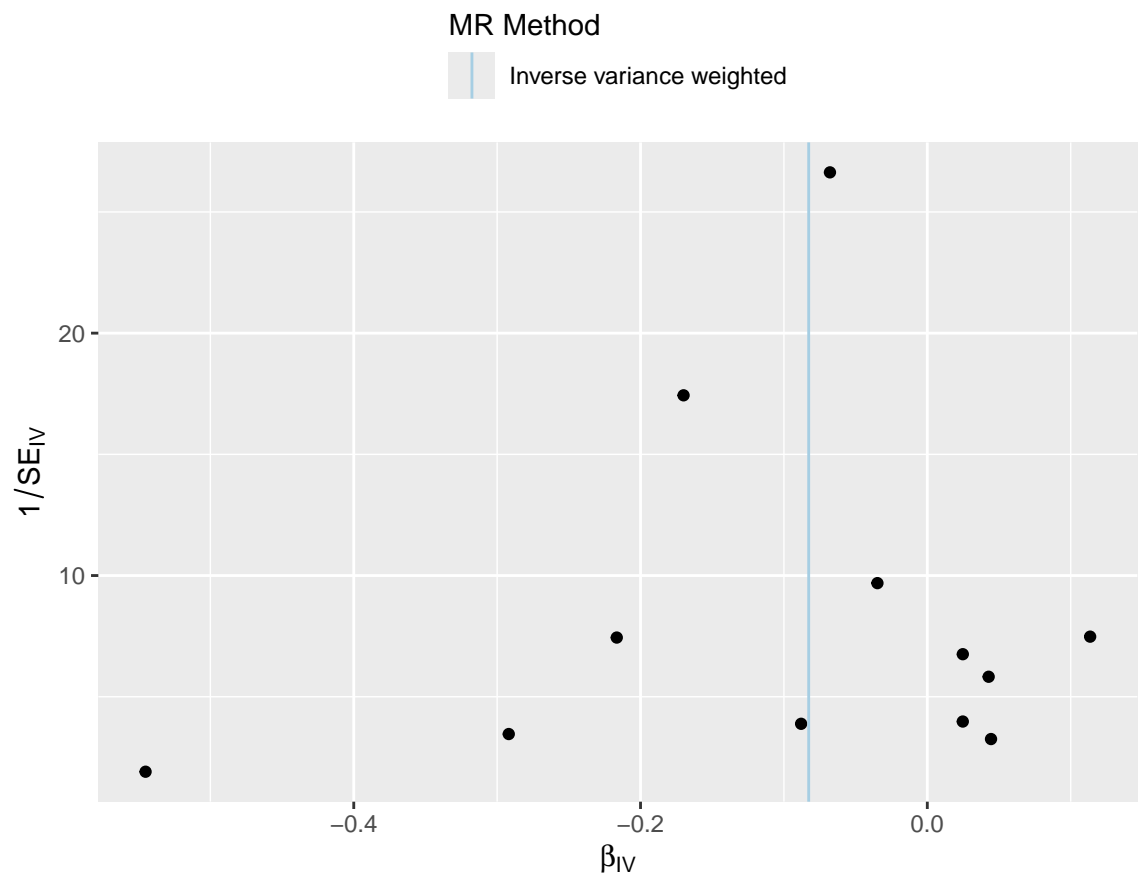

Supplement: Supplementary file 4 [file Datasheet4.zip › Supplementary documents1/eQTL_LOO/CBR4_LOO_MR.pdf]

COX11 – Leave-One-Out MR

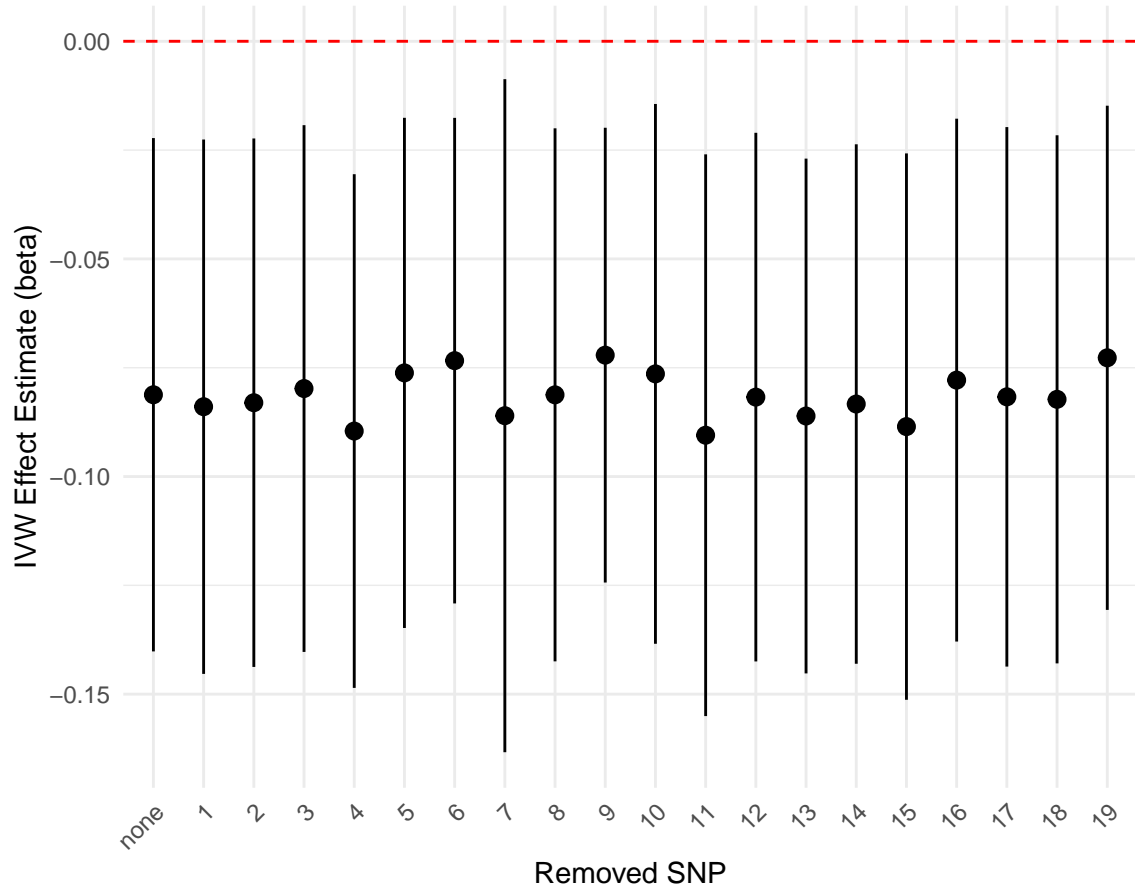

COX11 – Forest Plot

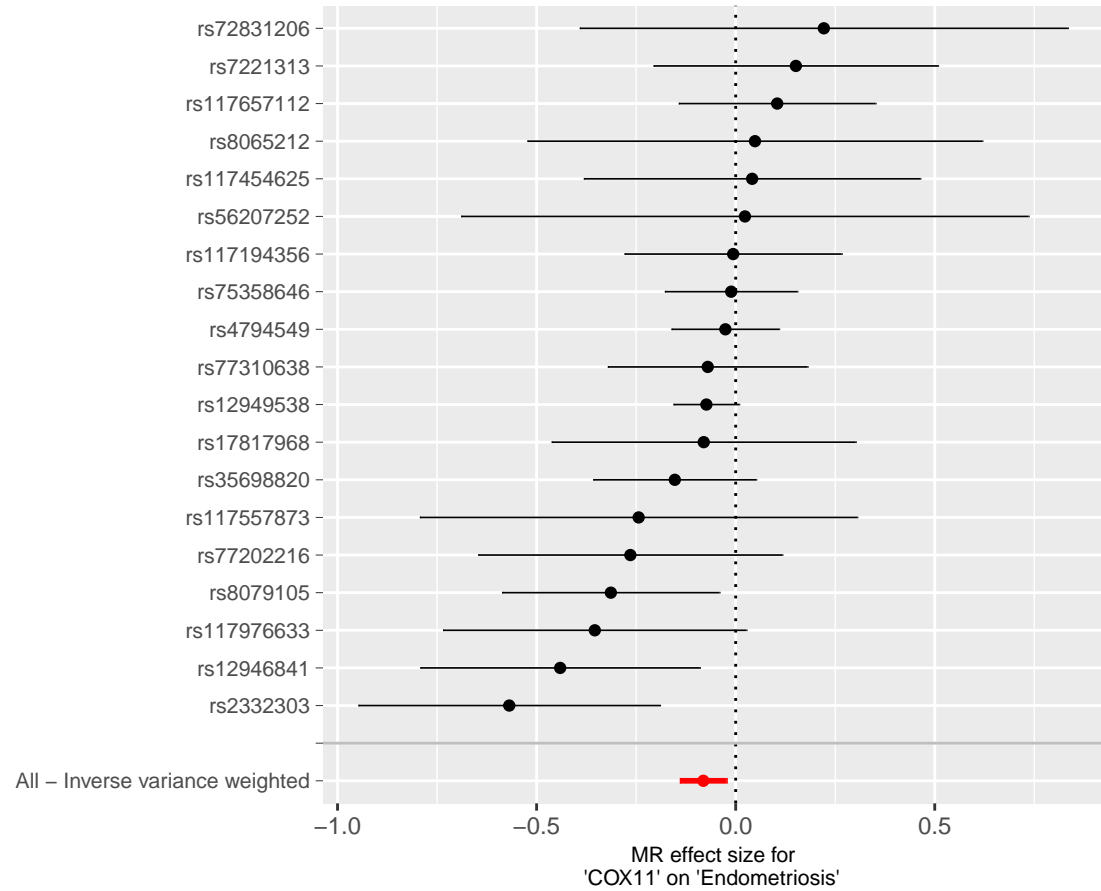

COX11 – Funnel Plot

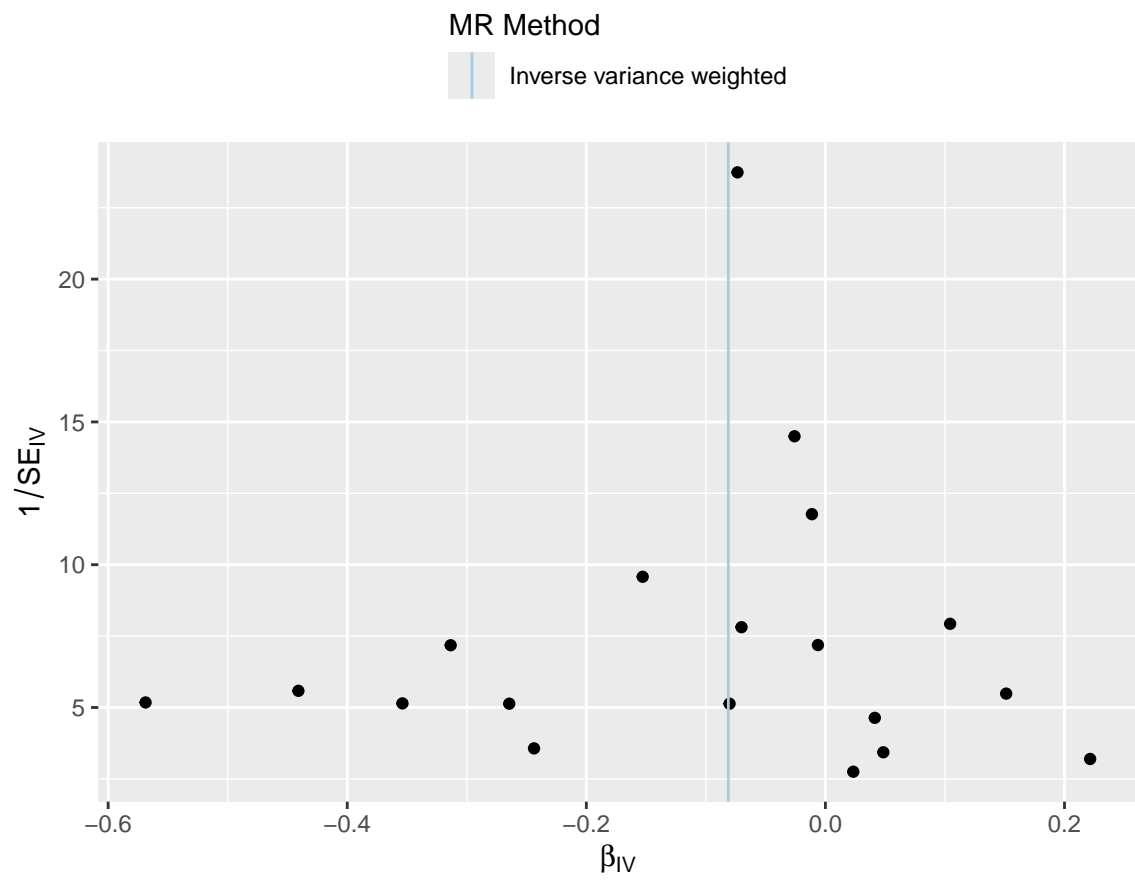

Supplement: Supplementary file 4 [file Datasheet4.zip › Supplementary documents1/eQTL_LOO/COX11_LOO_MR.pdf]

DNA2 – Leave-One-Out MR

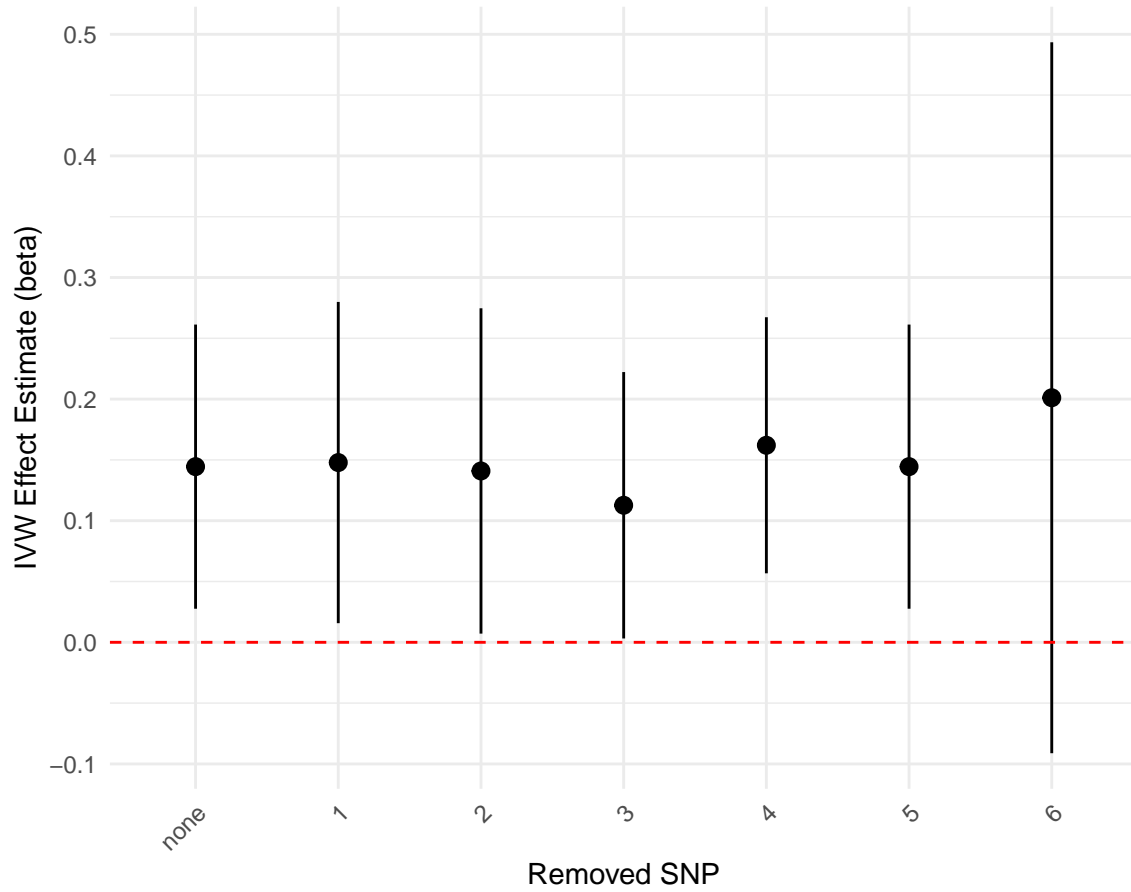

DNA2 – Forest Plot

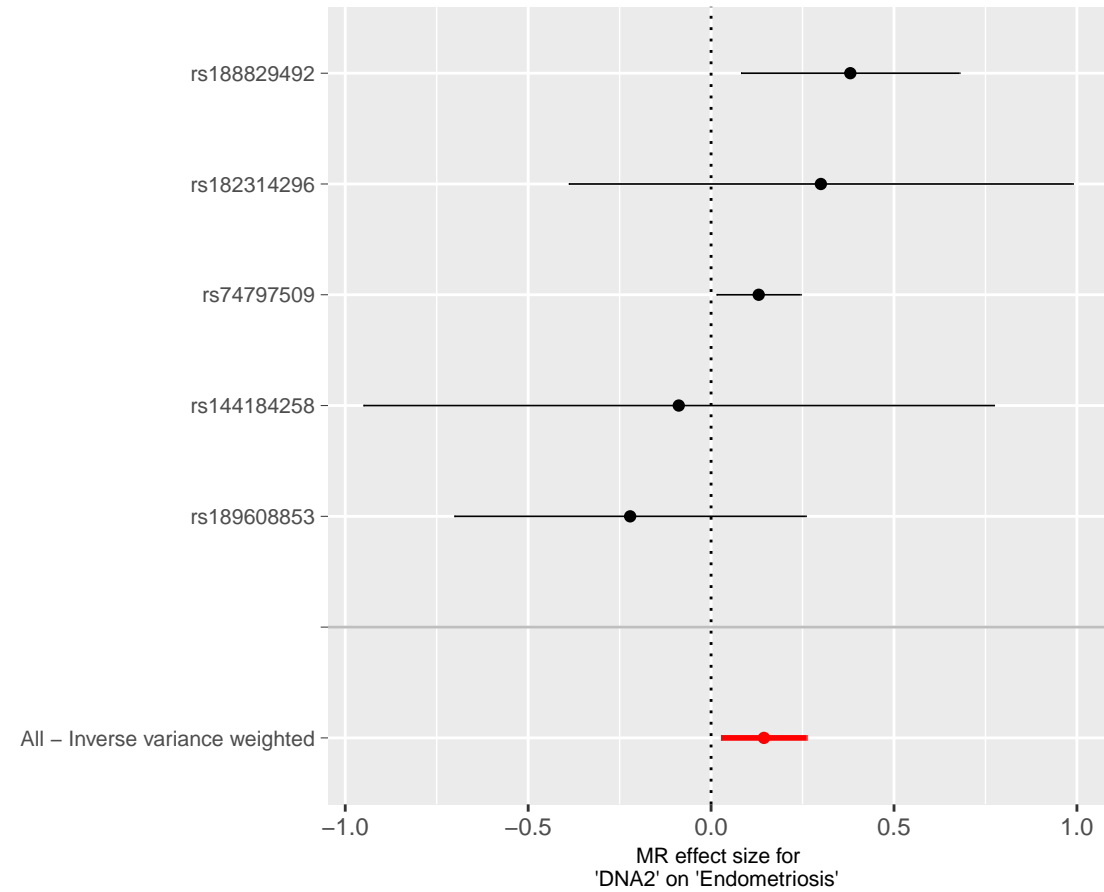

DNA2 – Funnel Plot

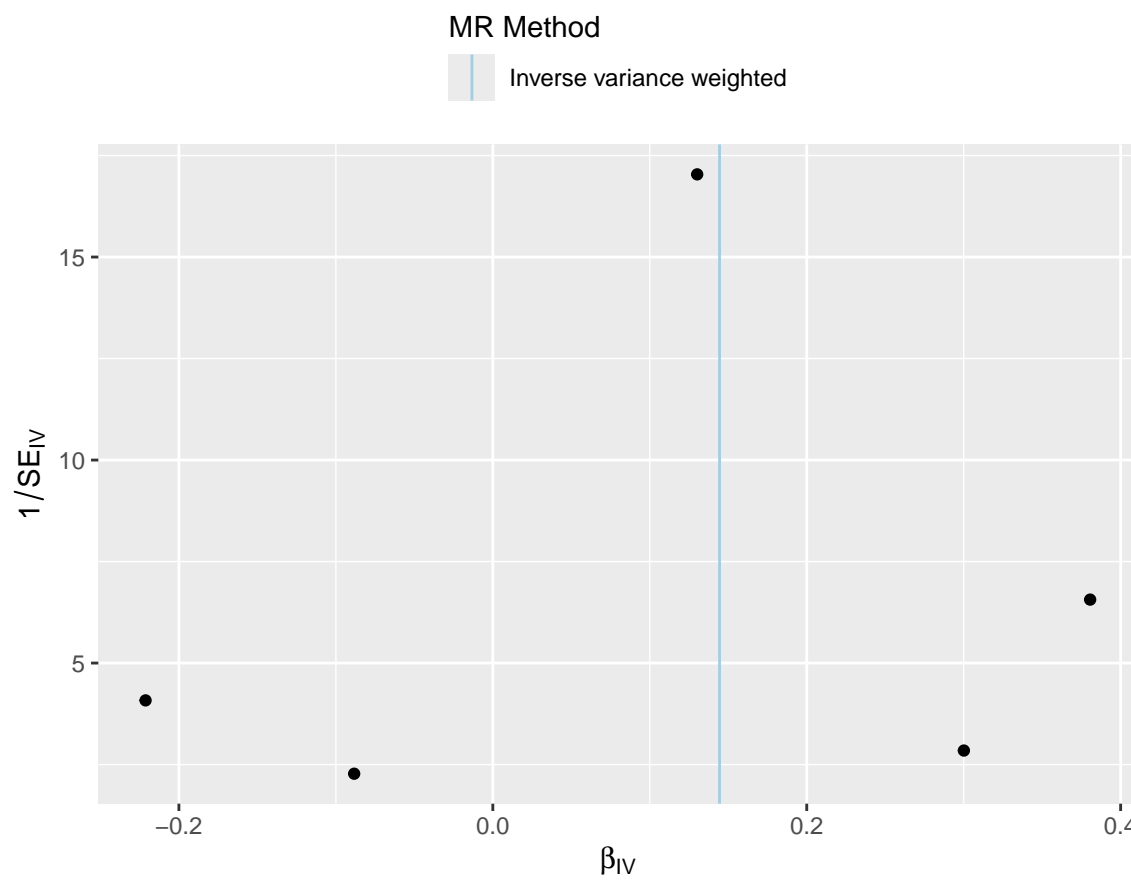

Supplement: Supplementary file 4 [file Datasheet4.zip › Supplementary documents1/eQTL_LOO/DNA2_LOO_MR.pdf]

### EHHADH – Leave-One-Out MR

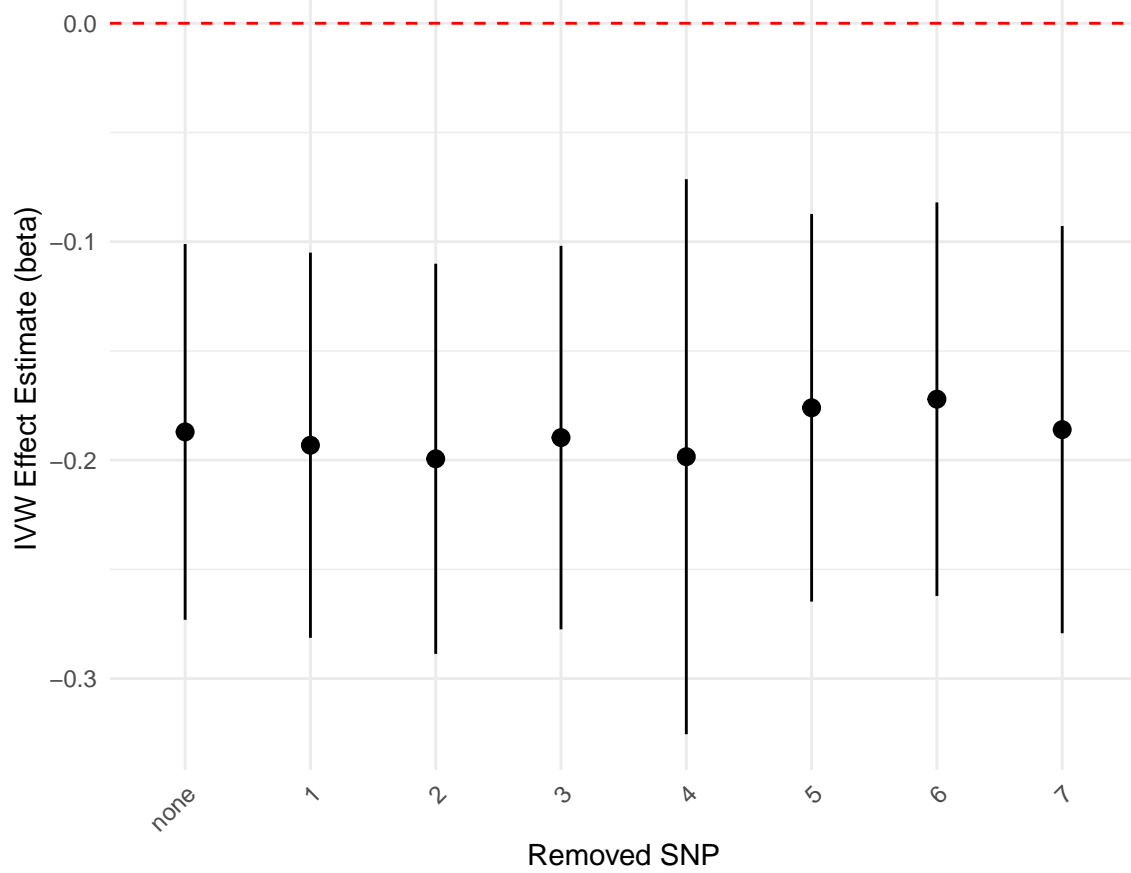

### EHHADH – Forest Plot

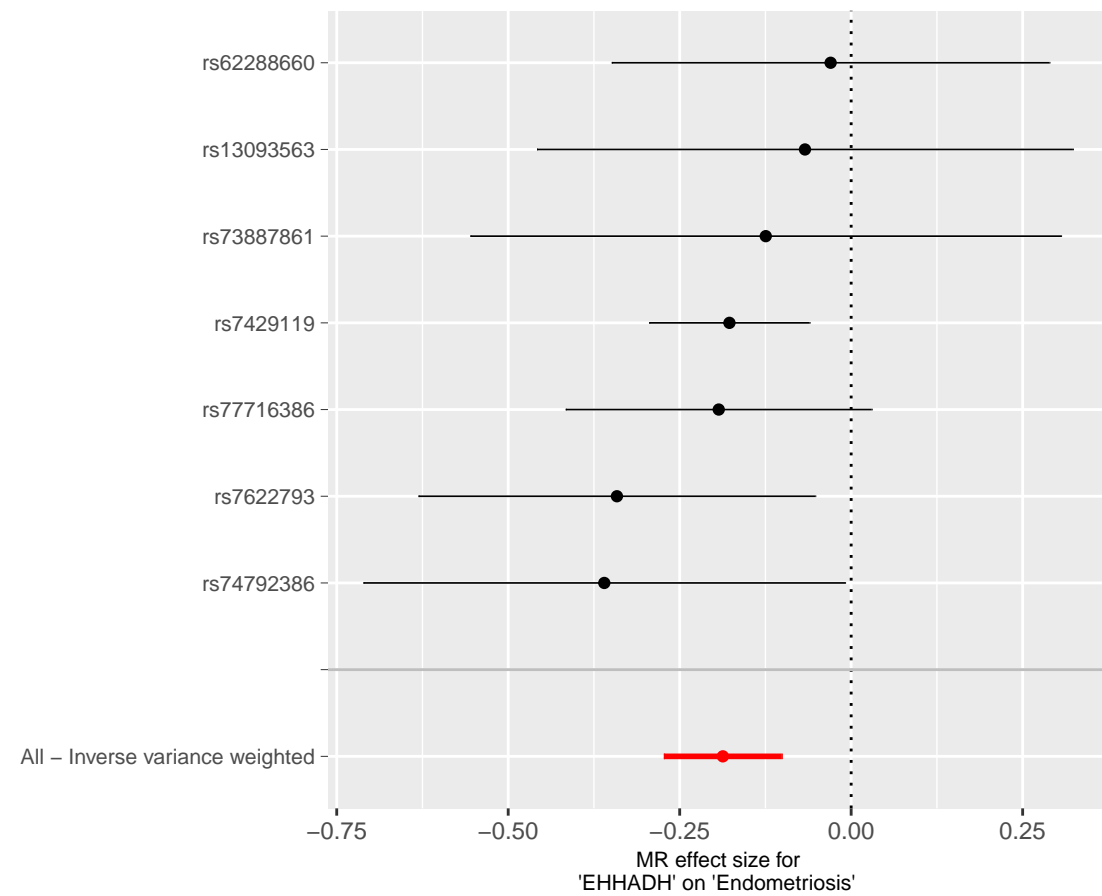

### EHHADH – Funnel Plot

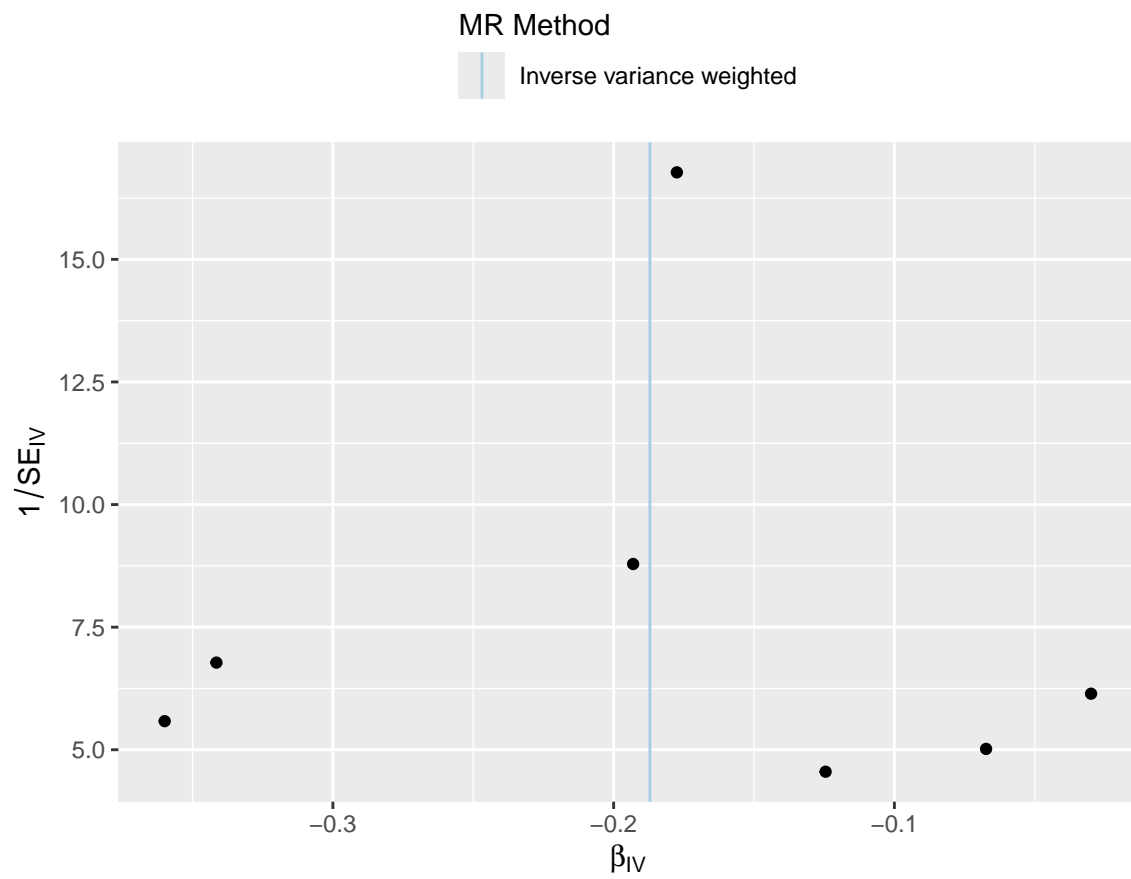

Supplement: Supplementary file 4 [file Datasheet4.zip › Supplementary documents1/eQTL_LOO/EHHADH_LOO_MR.pdf]

ERAL1 – Leave-One-Out MR

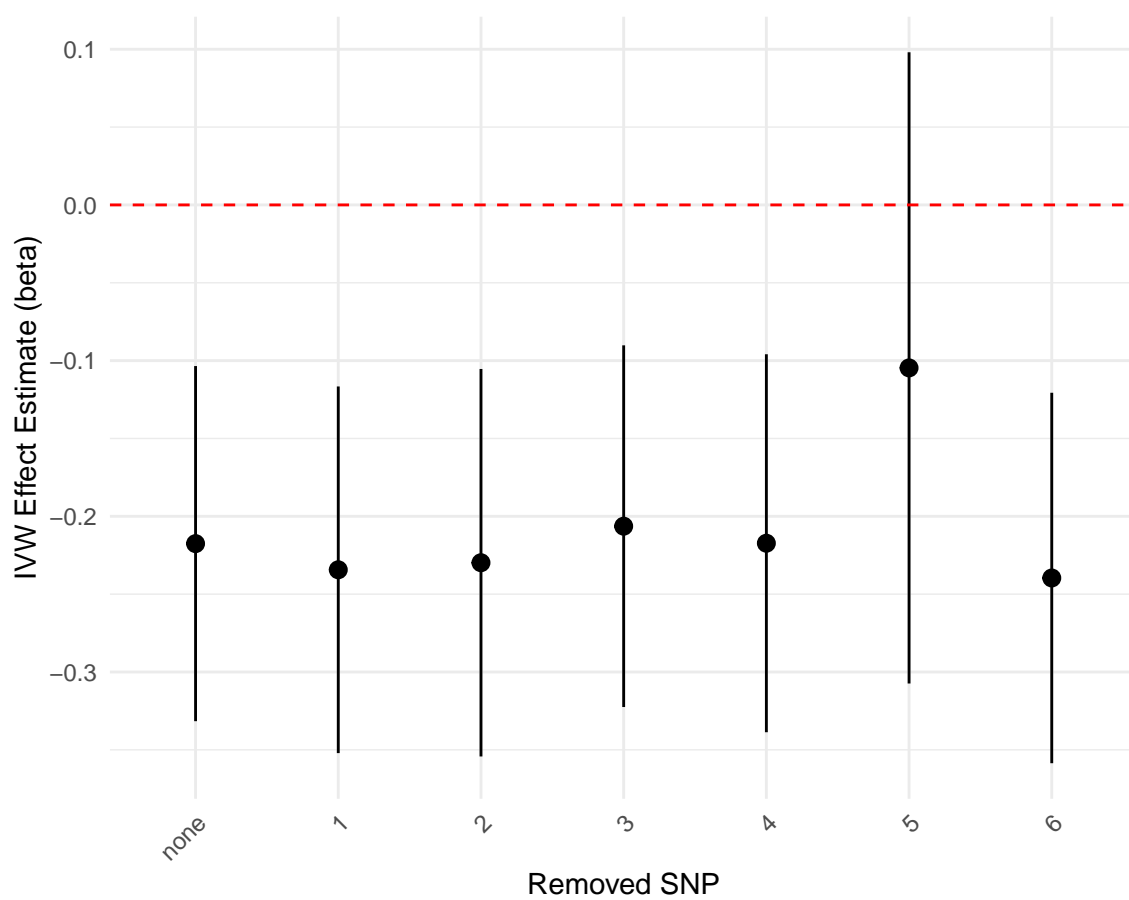

ERAL1 – Forest Plot

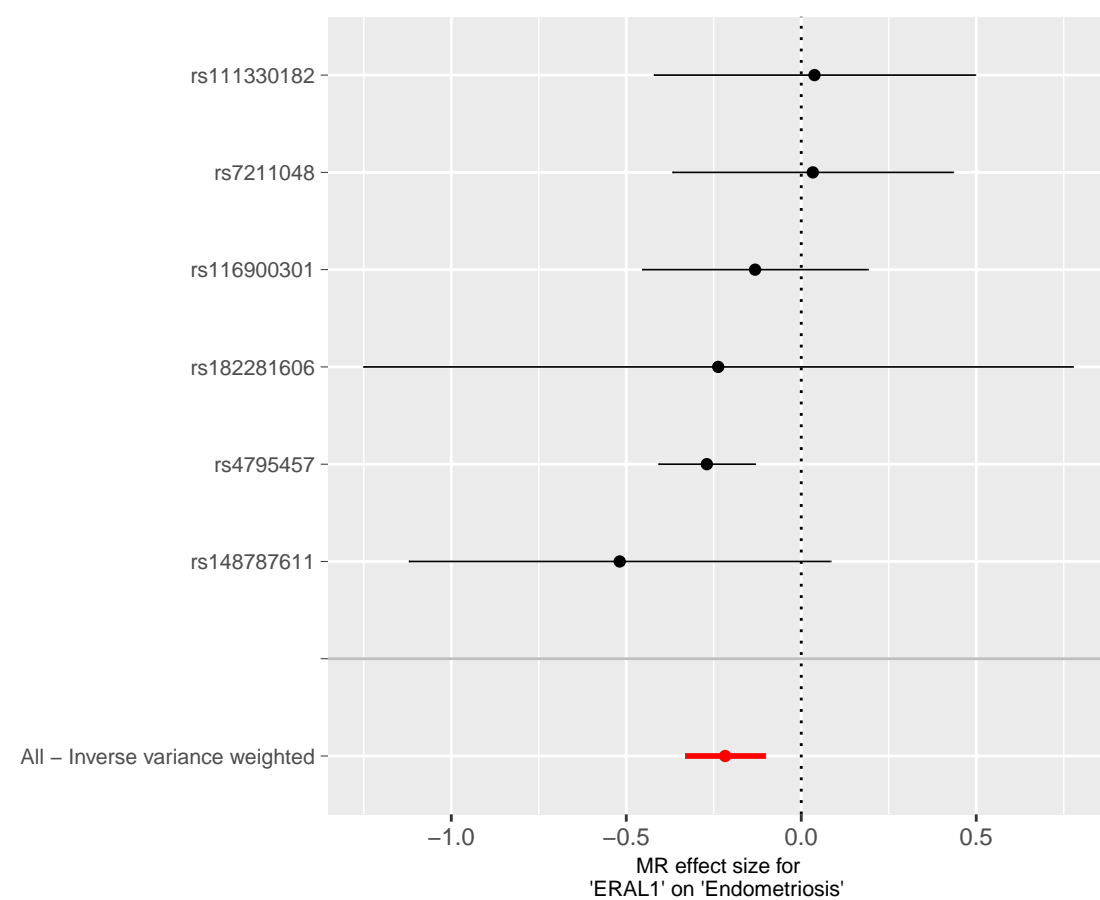

ERAL1 – Funnel Plot

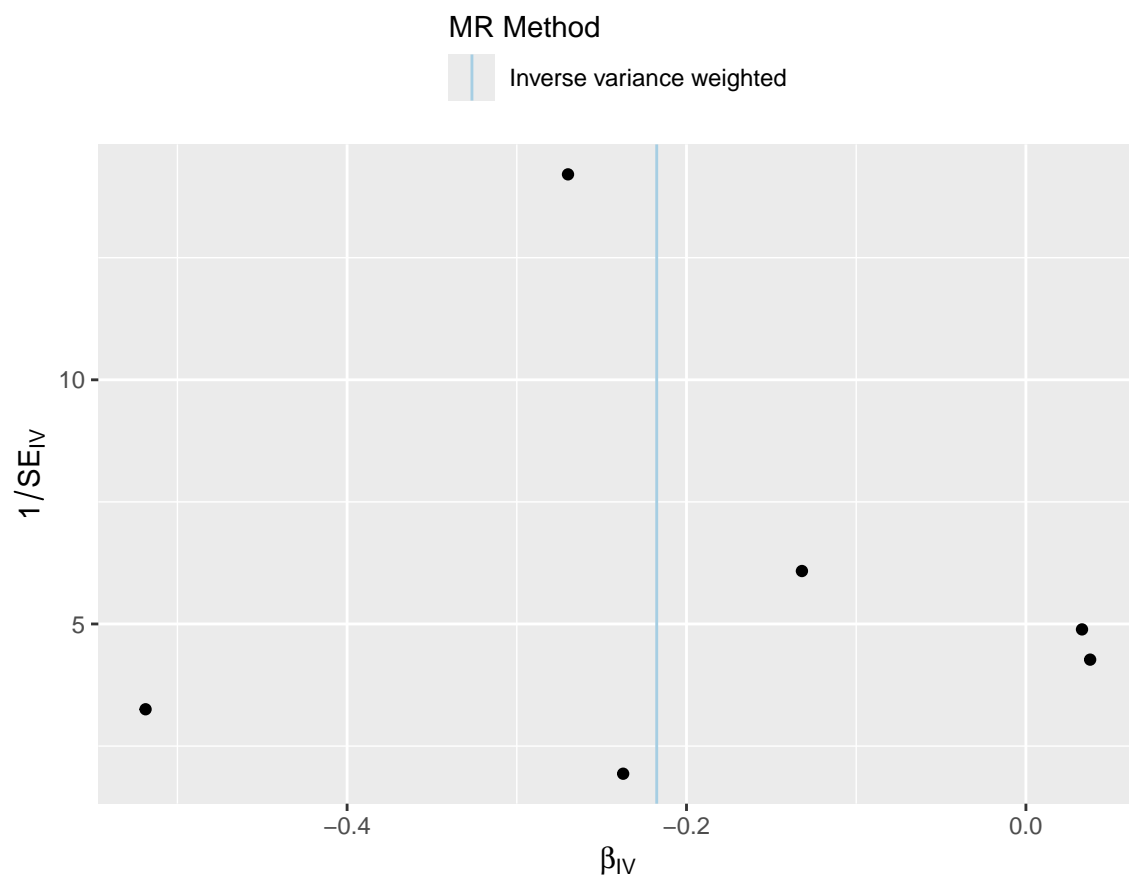

Supplement: Supplementary file 4 [file Datasheet4.zip › Supplementary documents1/eQTL_LOO/ERAL1_LOO_MR.pdf]

### FIS1 – Leave-One-Out MR

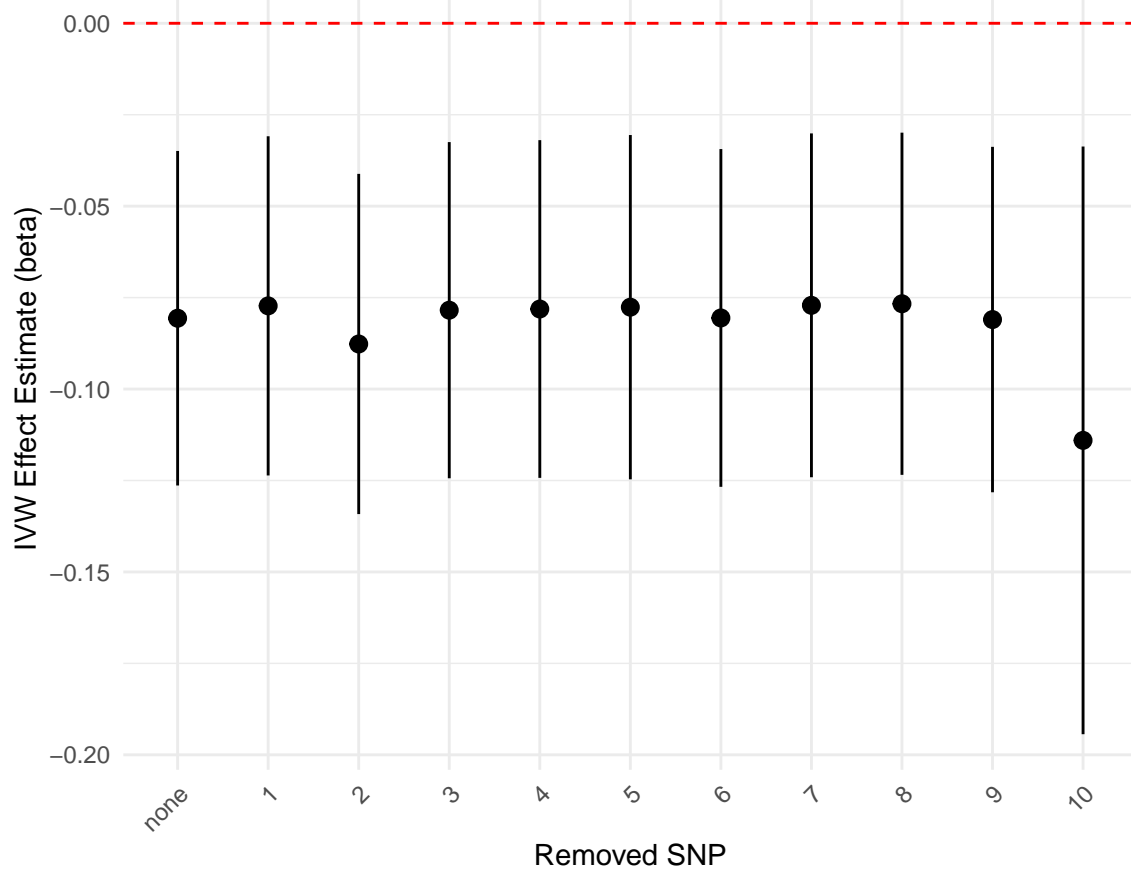

### FIS1 – Forest Plot

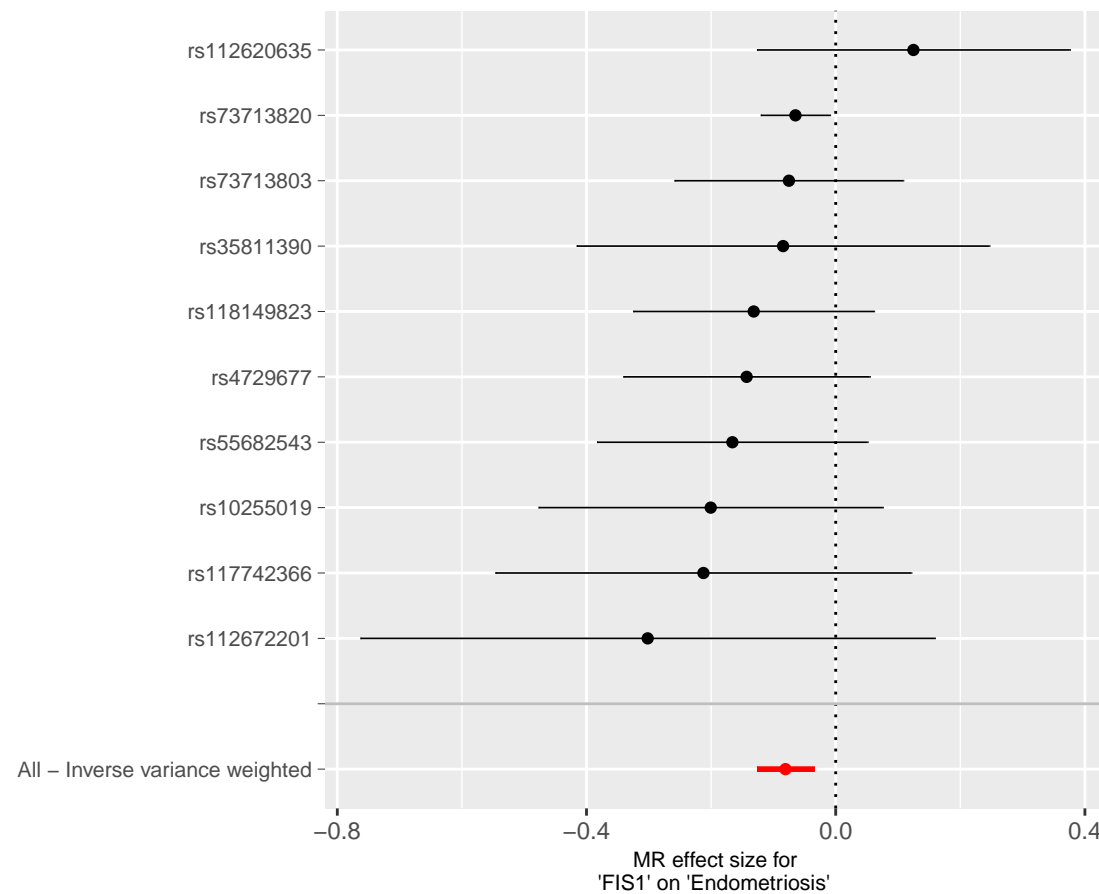

### FIS1 – Funnel Plot

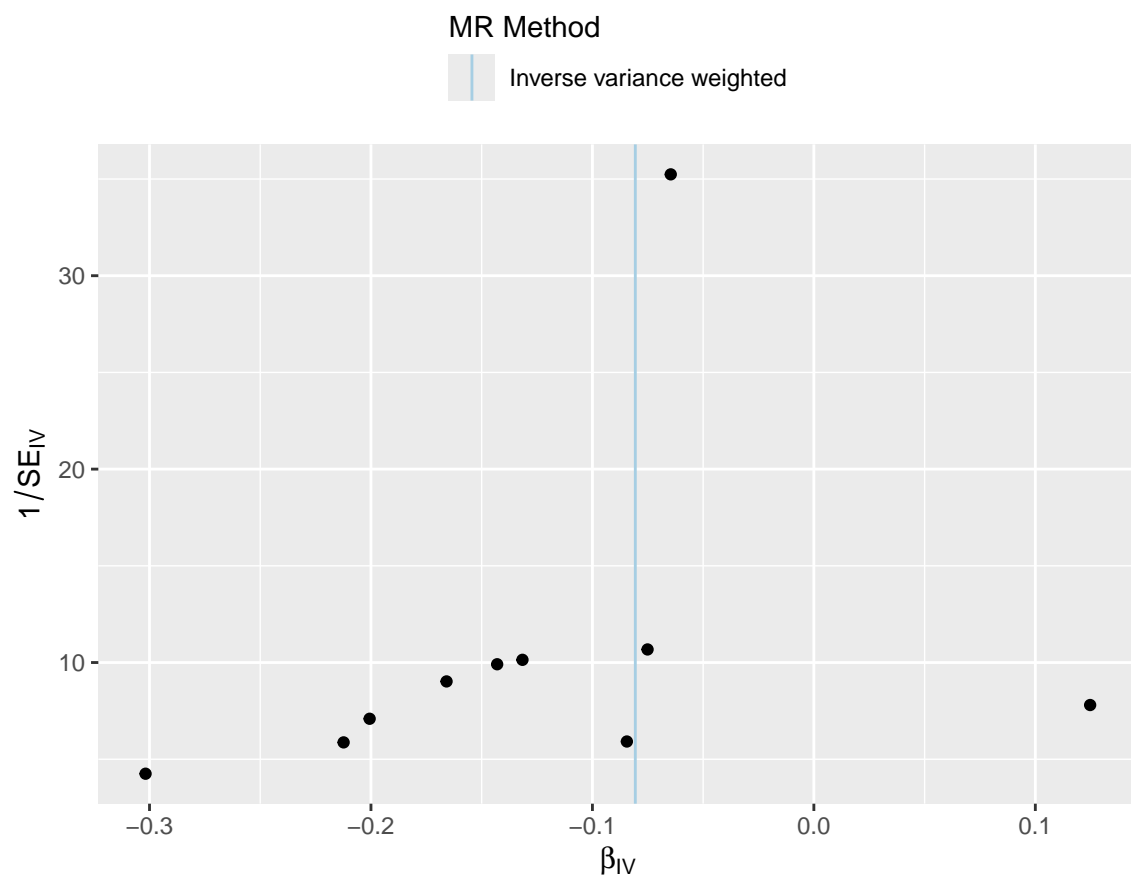

Supplement: Supplementary file 4 [file Datasheet4.zip › Supplementary documents1/eQTL_LOO/FIS1_LOO_MR.pdf]

IMMT – Leave-One-Out MR

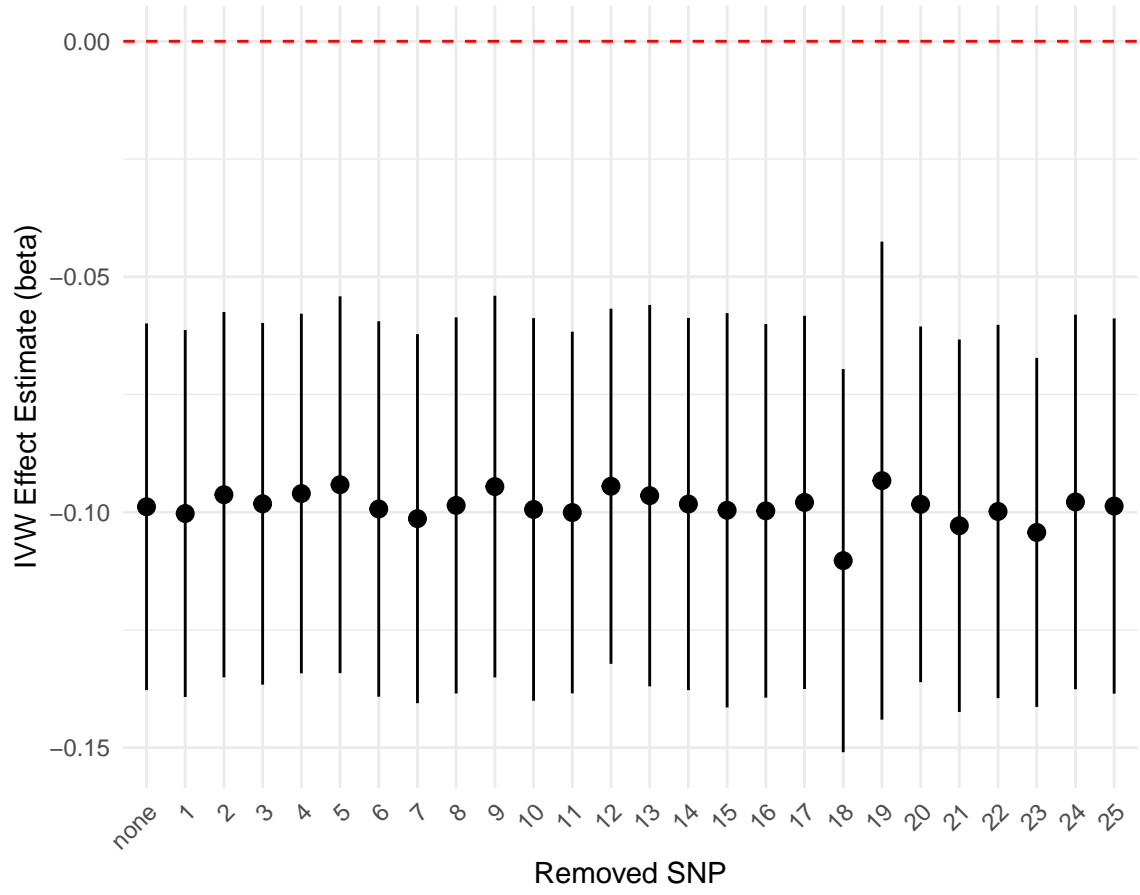

IMMT – Forest Plot

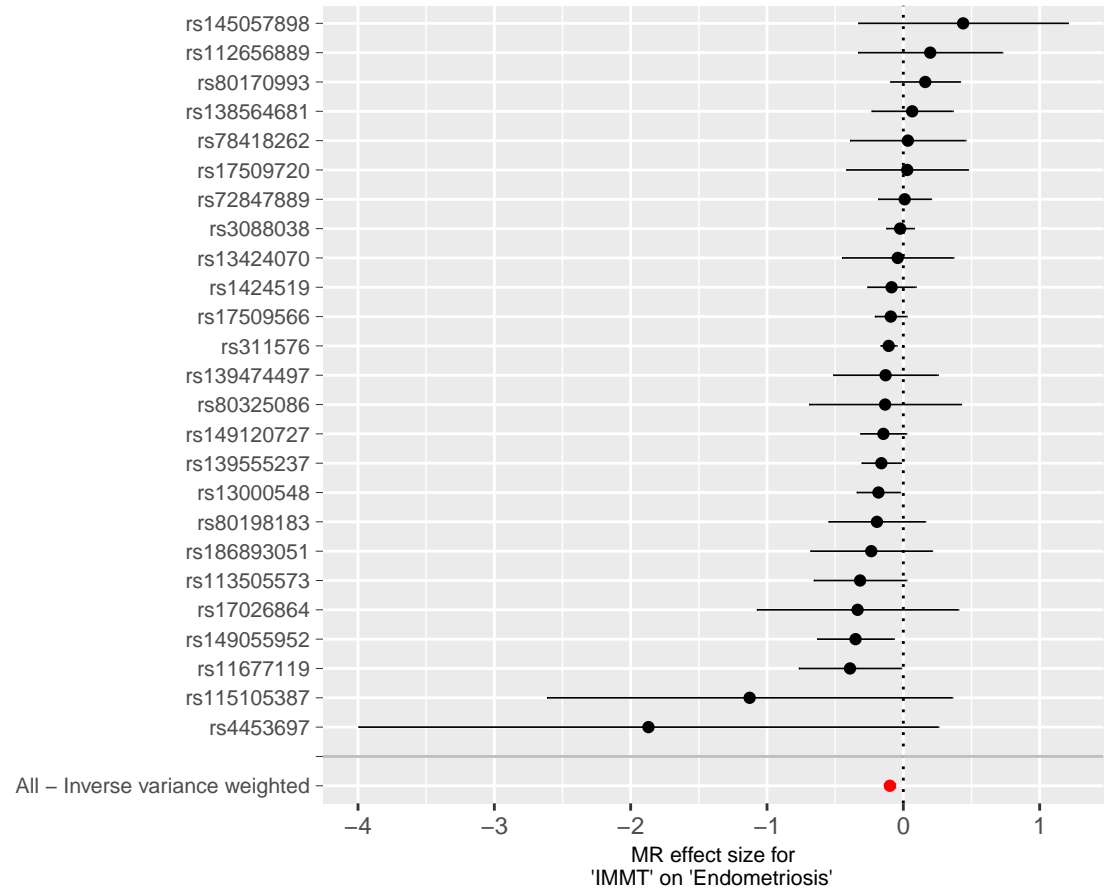

IMMT – Funnel Plot

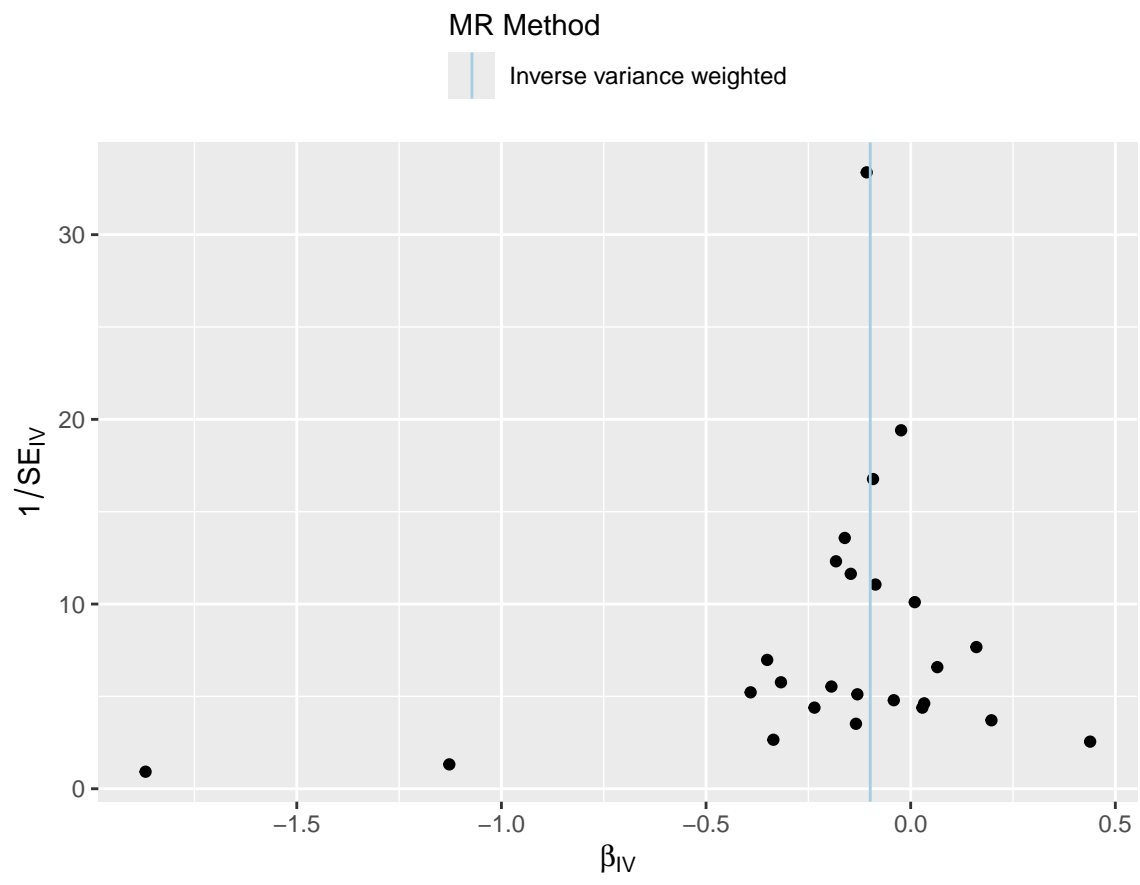

Supplement: Supplementary file 4 [file Datasheet4.zip › Supplementary documents1/eQTL_LOO/IMMT_LOO_MR.pdf]

LYRM7 – Leave-One-Out MR

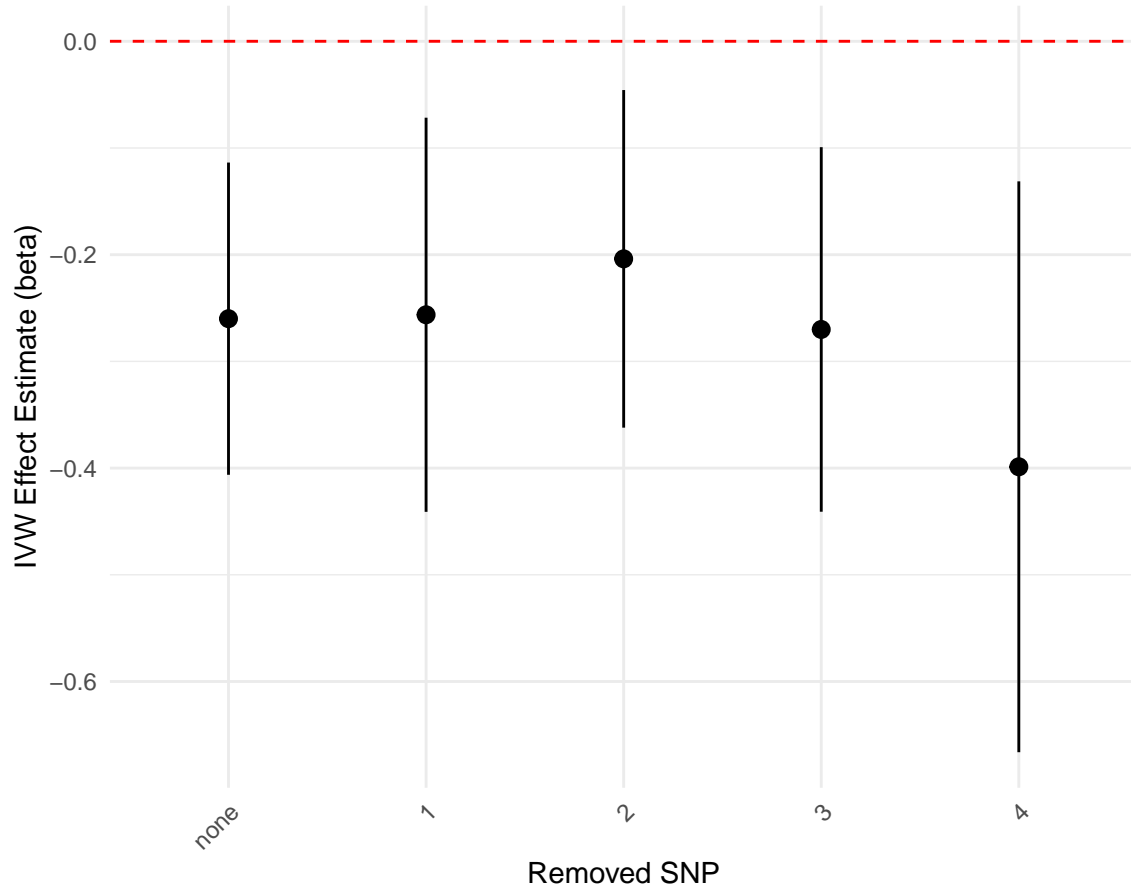

LYRM7 – Forest Plot

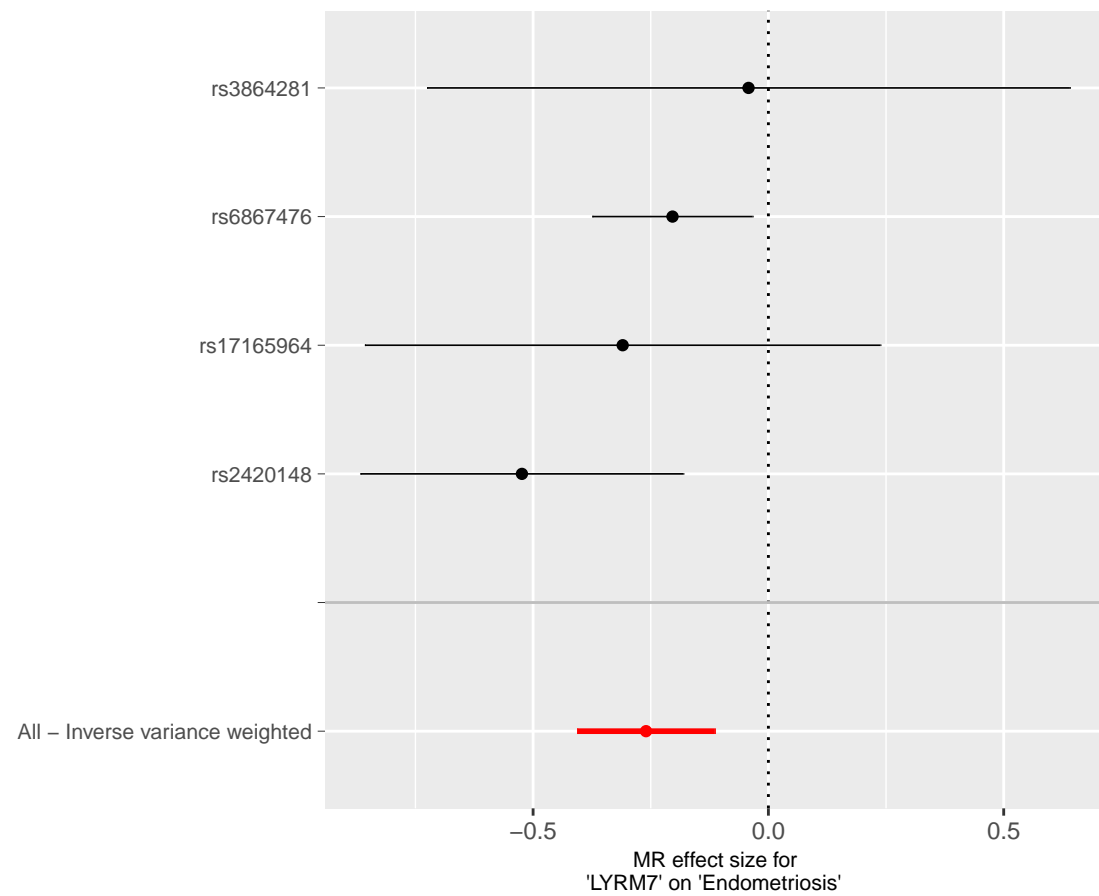

LYRM7 – Funnel Plot

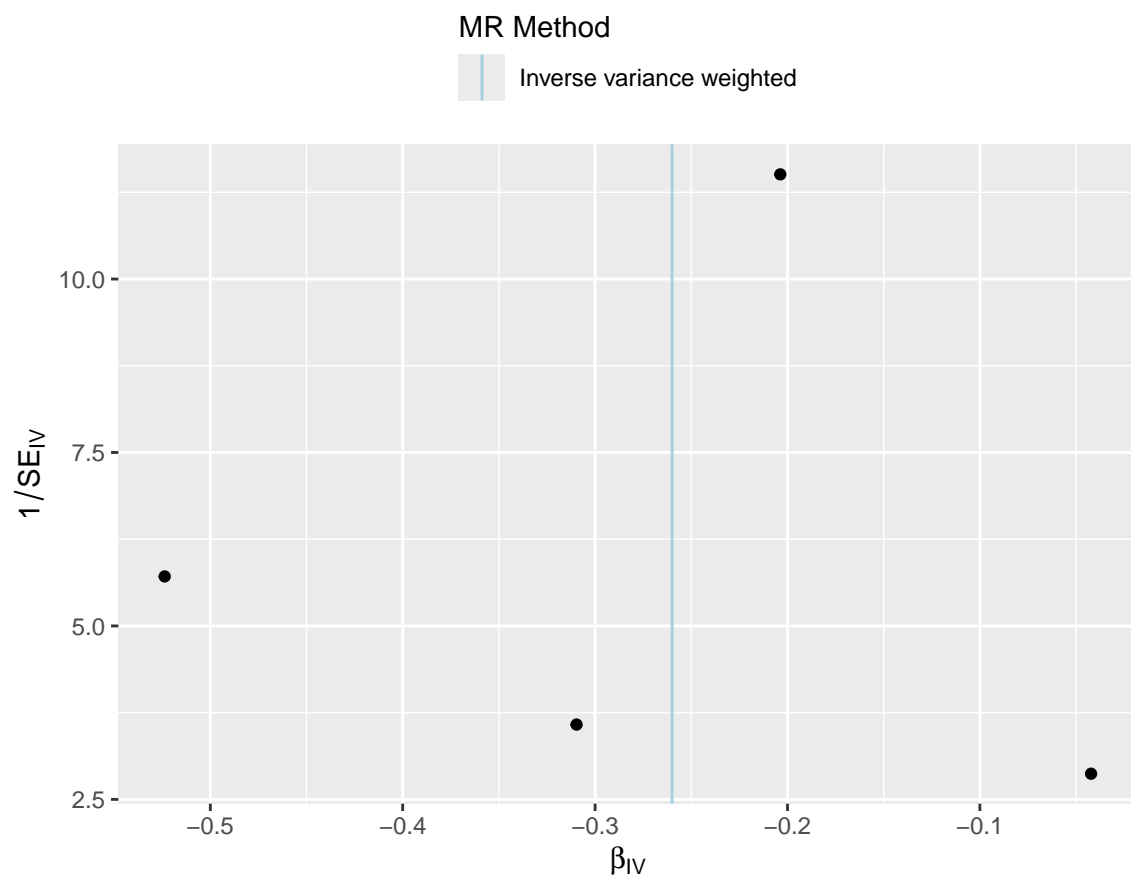

Supplement: Supplementary file 4 [file Datasheet4.zip › Supplementary documents1/eQTL_LOO/LYRM7_LOO_MR.pdf]

ME2 – Leave-One-Out MR

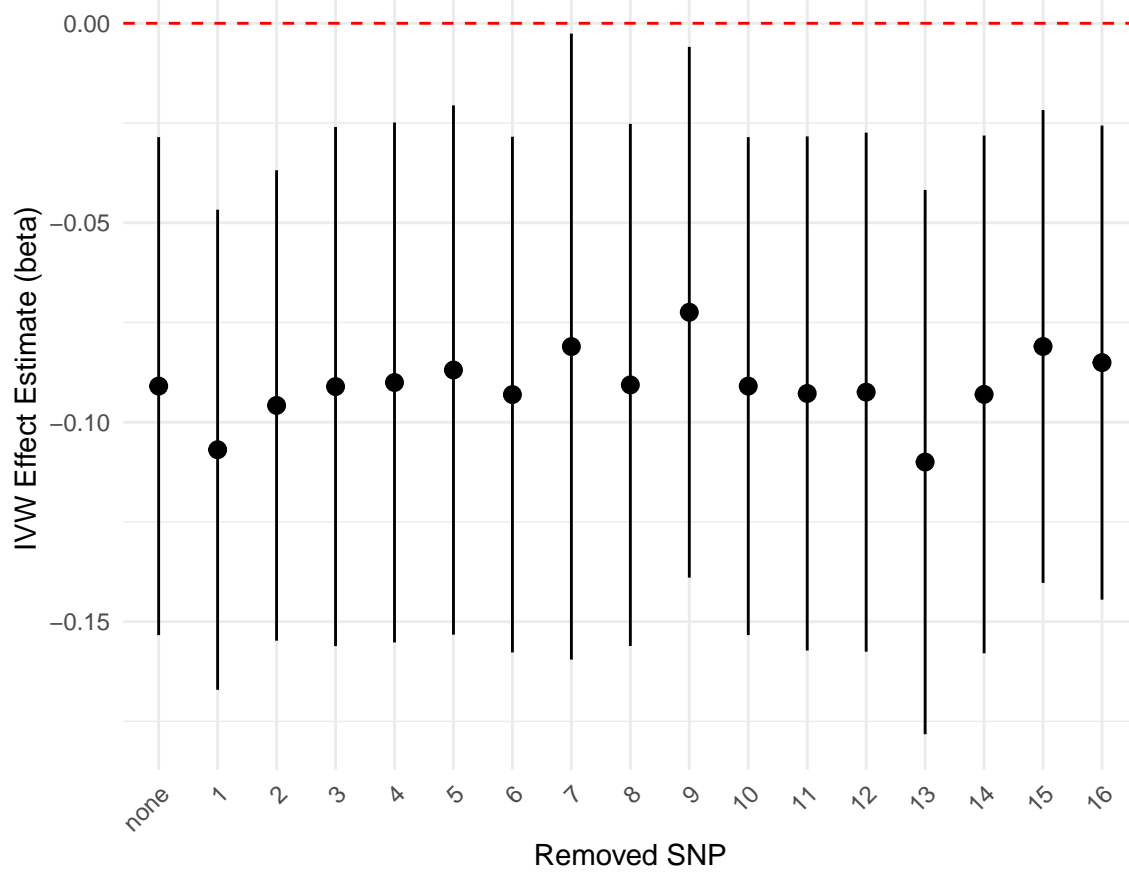

ME2 – Forest Plot

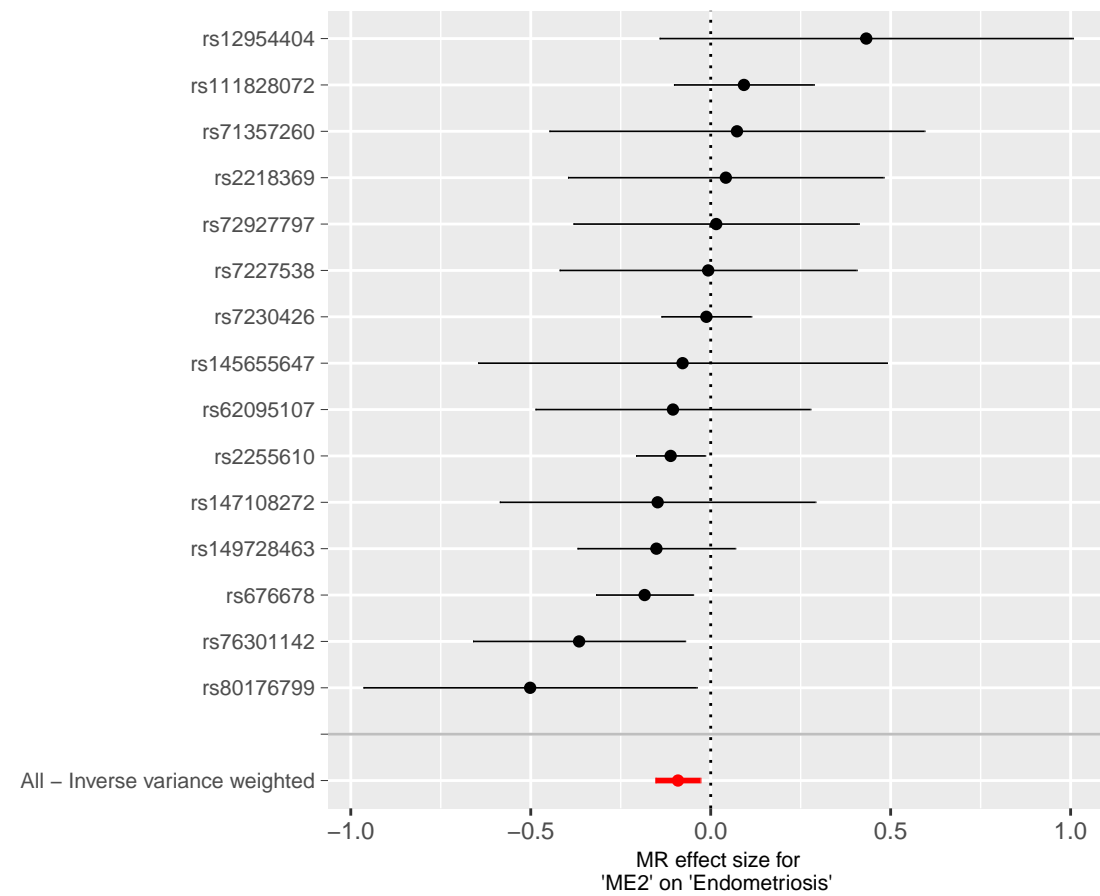

ME2 – Funnel Plot

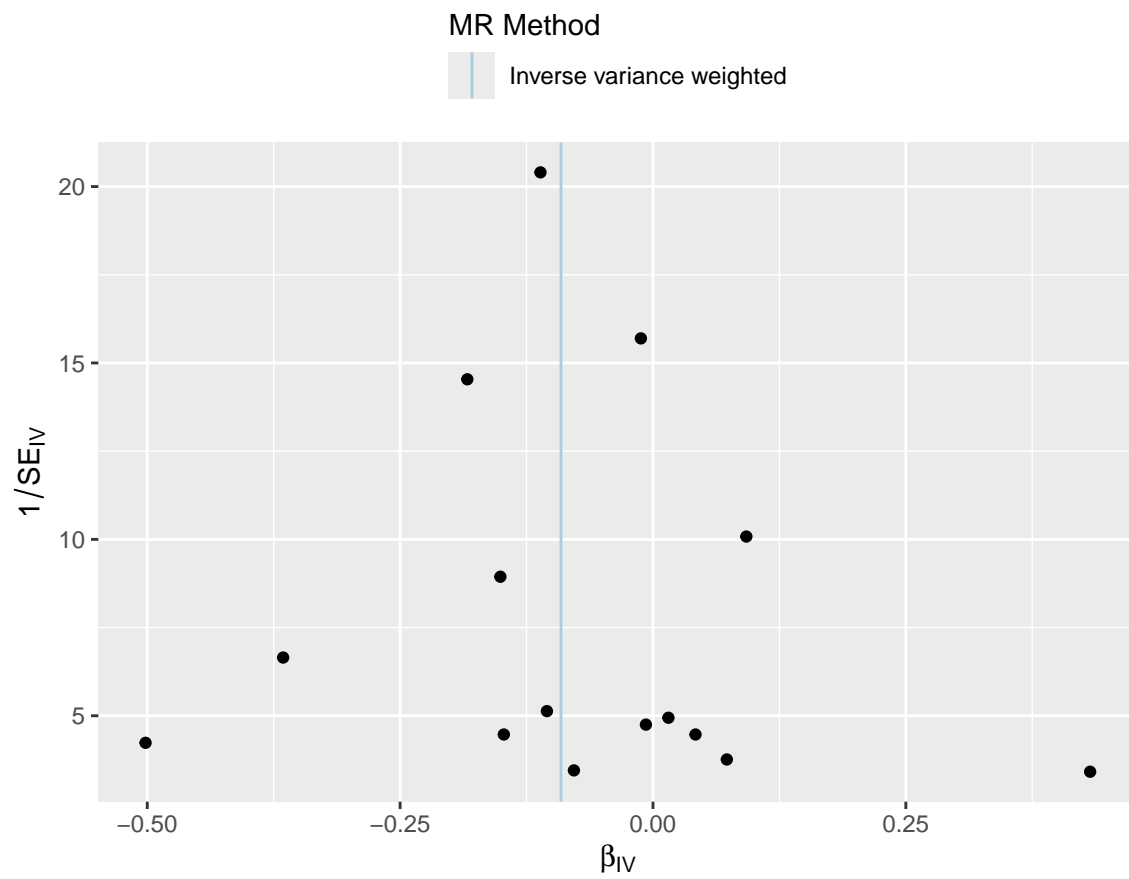

Supplement: Supplementary file 4 [file Datasheet4.zip › Supplementary documents1/eQTL_LOO/ME2_LOO_MR.pdf]

MRPL21 – Leave-One-Out MR

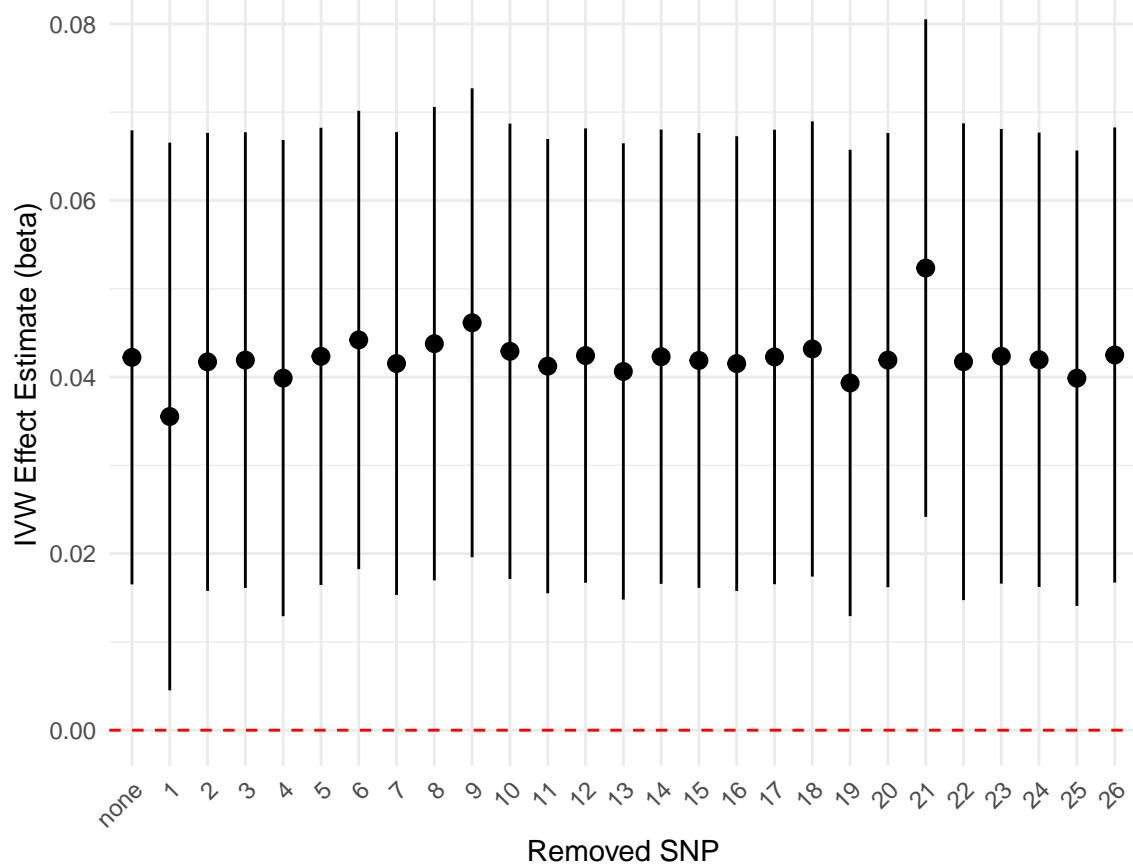

MRPL21 – Funnel Plot

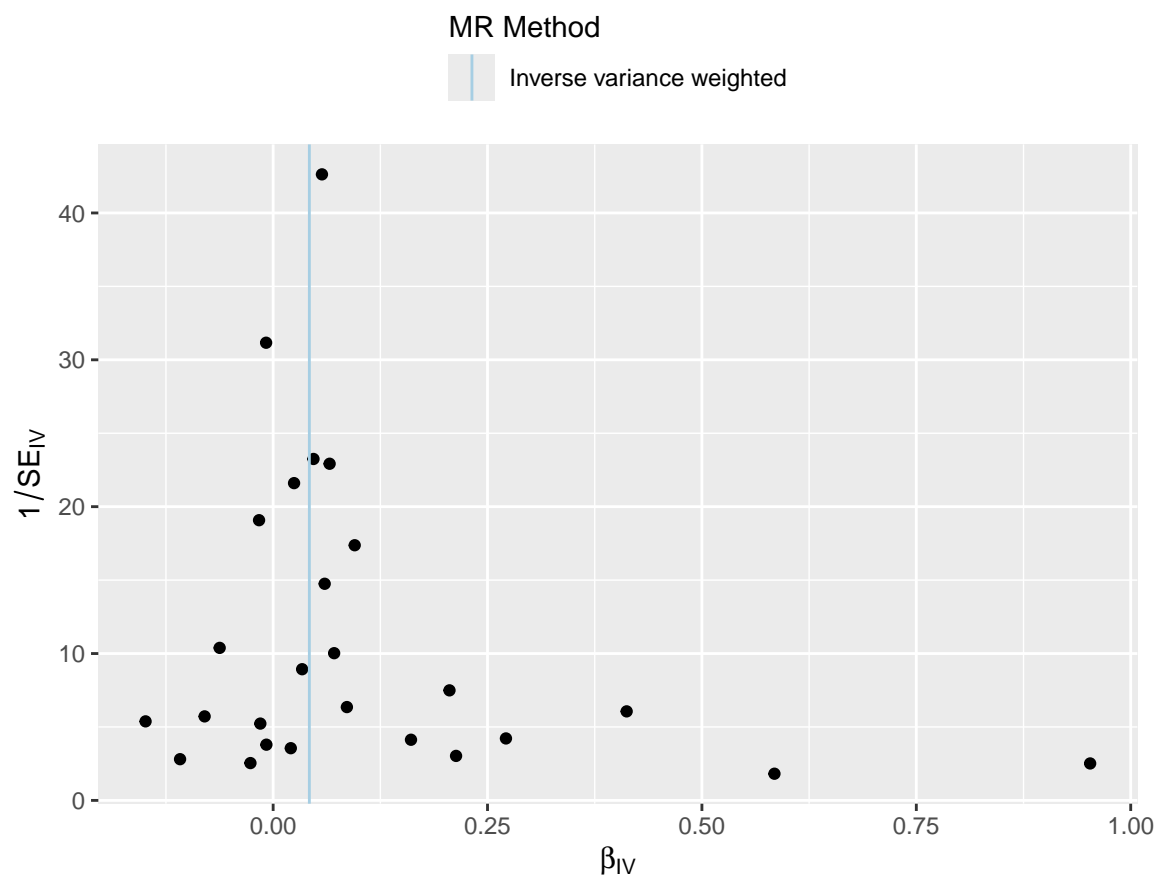

MRPL21 – Forest Plot

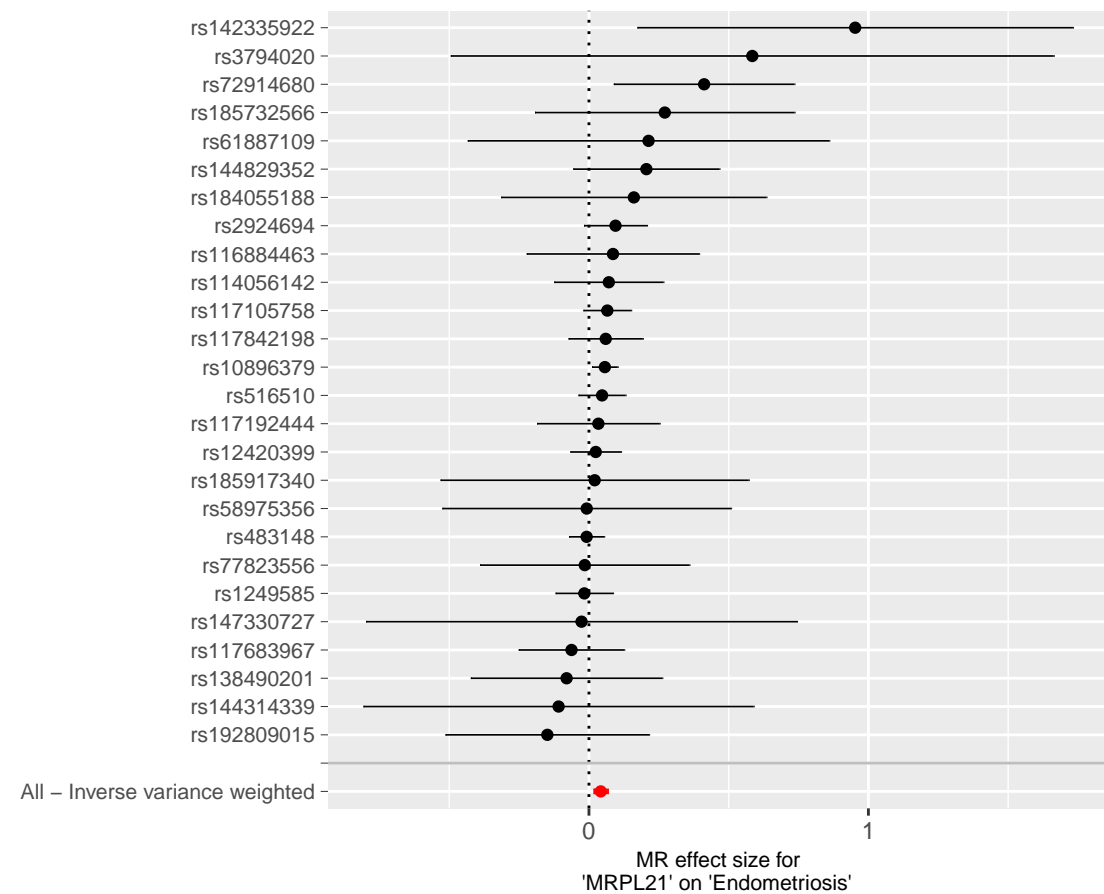

Supplement: Supplementary file 4 [file Datasheet4.zip › Supplementary documents1/eQTL_LOO/MRPL21_LOO_MR.pdf]

MRPL55 – Leave-One-Out MR

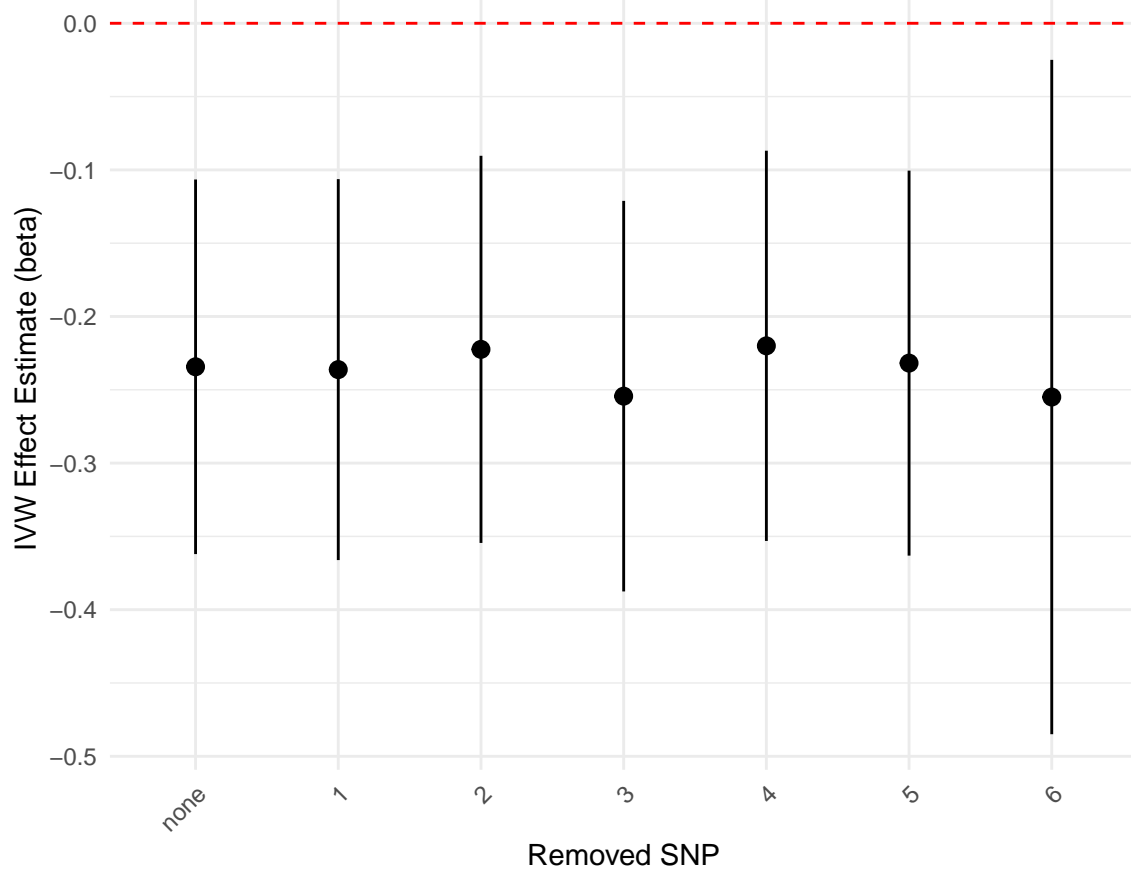

MRPL55 – Forest Plot

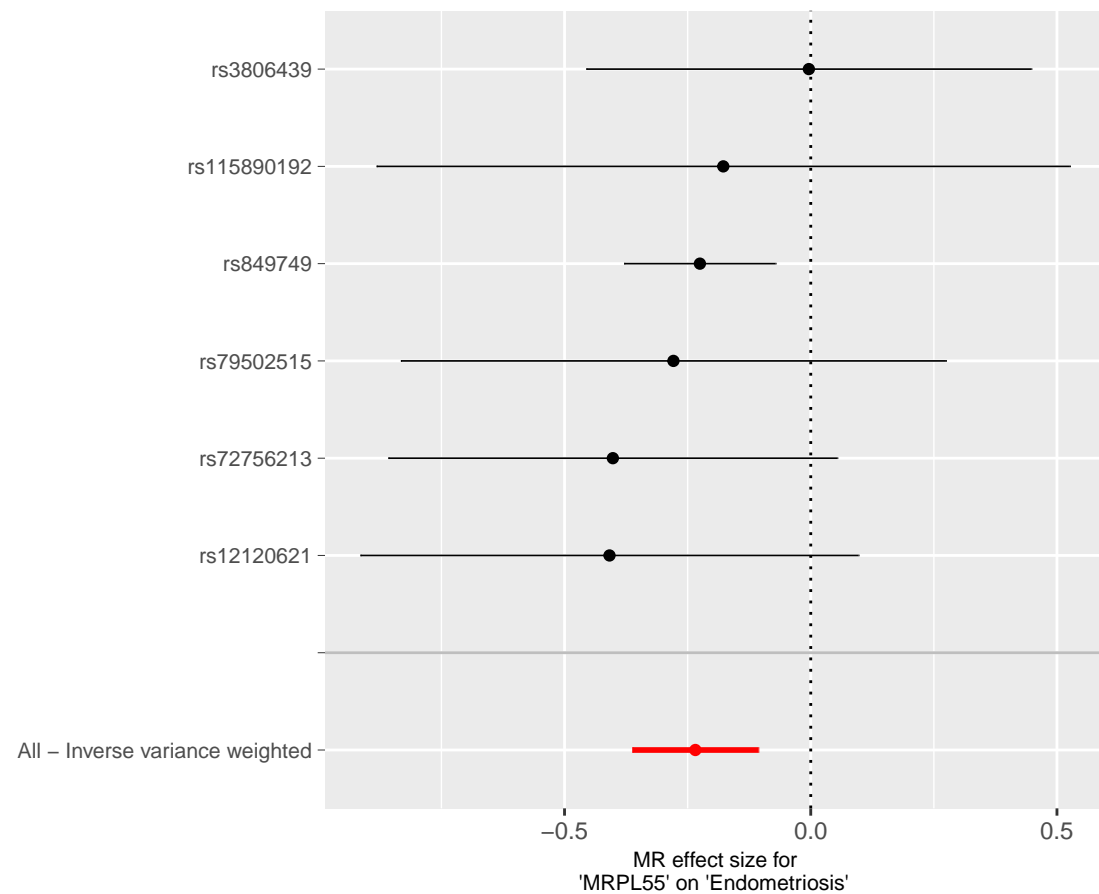

MRPL55 – Funnel Plot

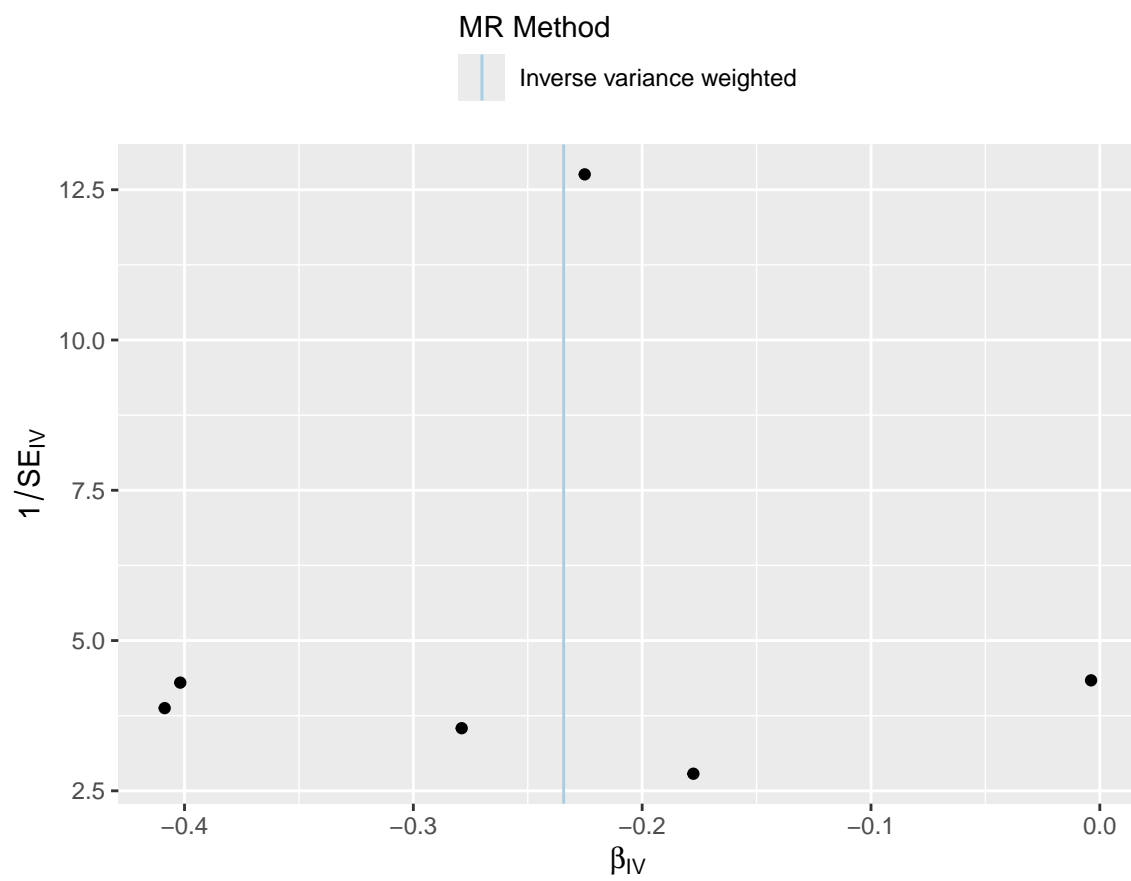

Supplement: Supplementary file 4 [file Datasheet4.zip › Supplementary documents1/eQTL_LOO/MRPL55_LOO_MR.pdf]

MRPS35 – Leave-One-Out MR

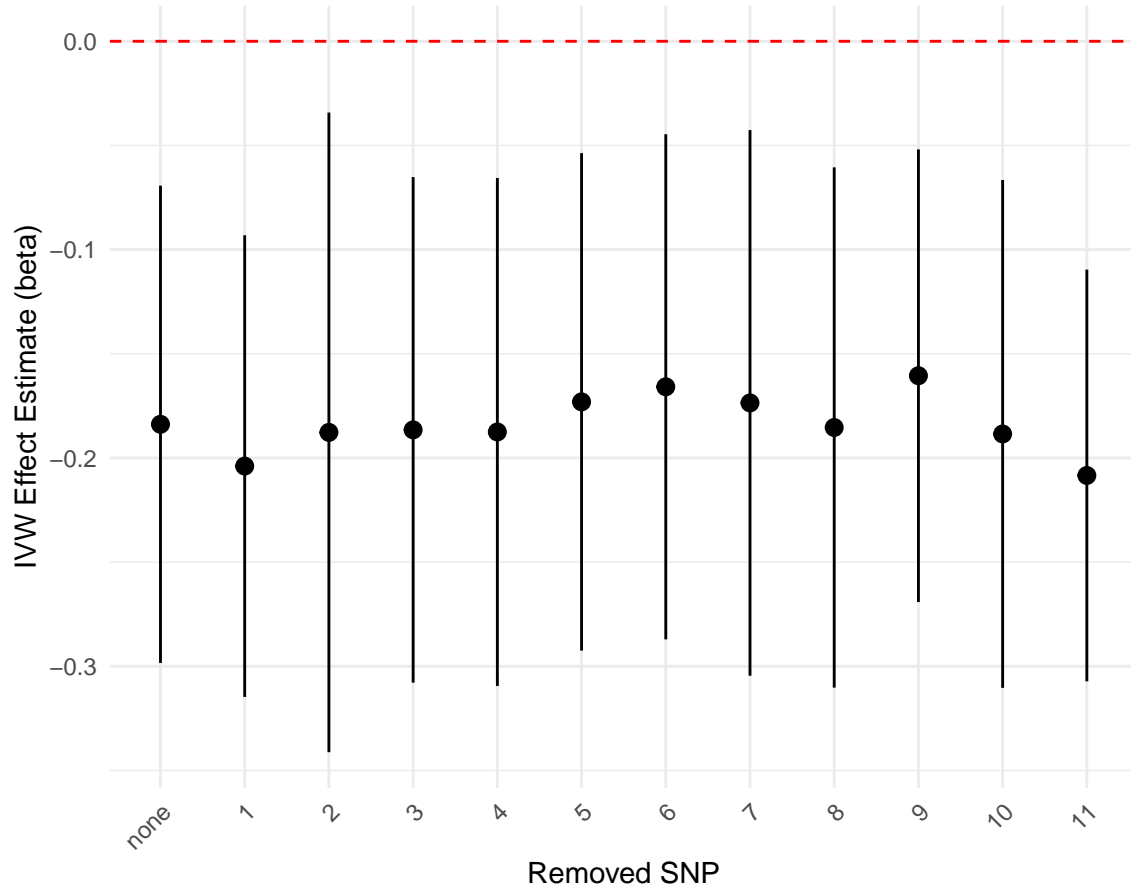

MRPS35 – Funnel Plot

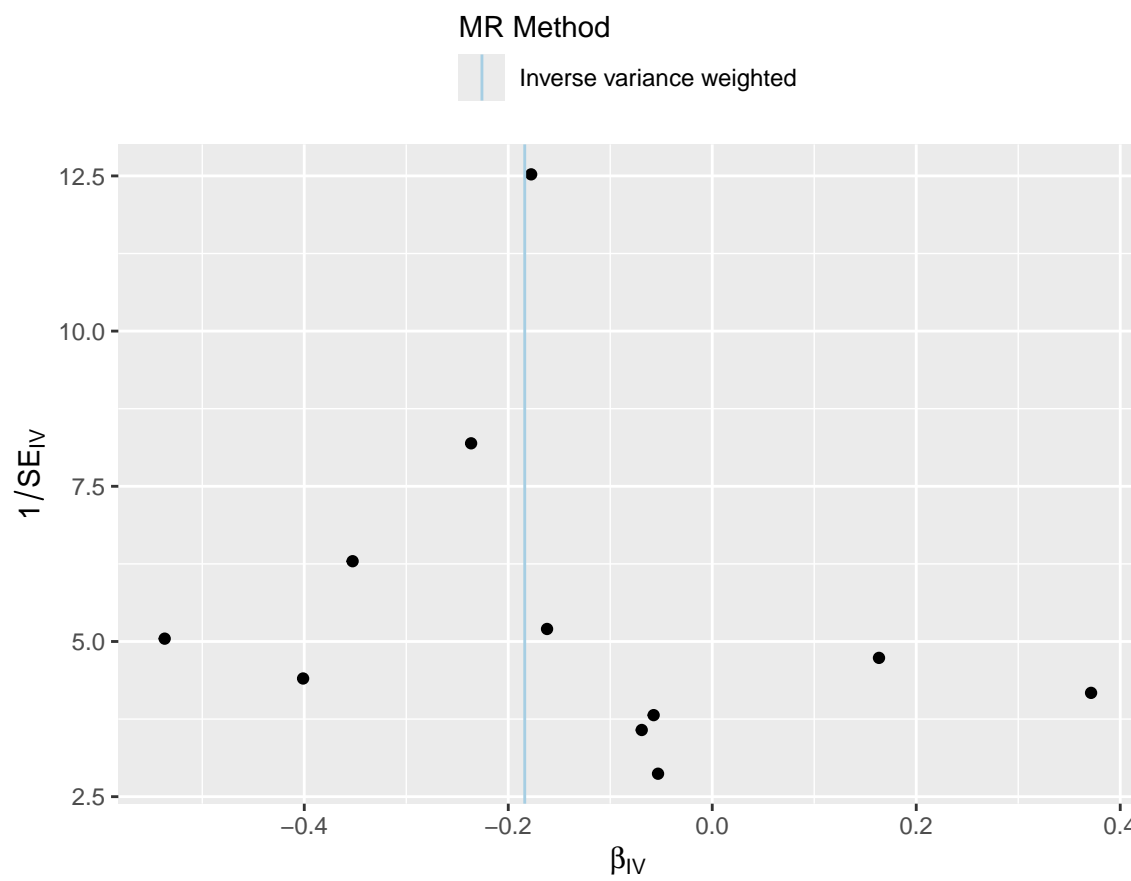

MRPS35 – Forest Plot

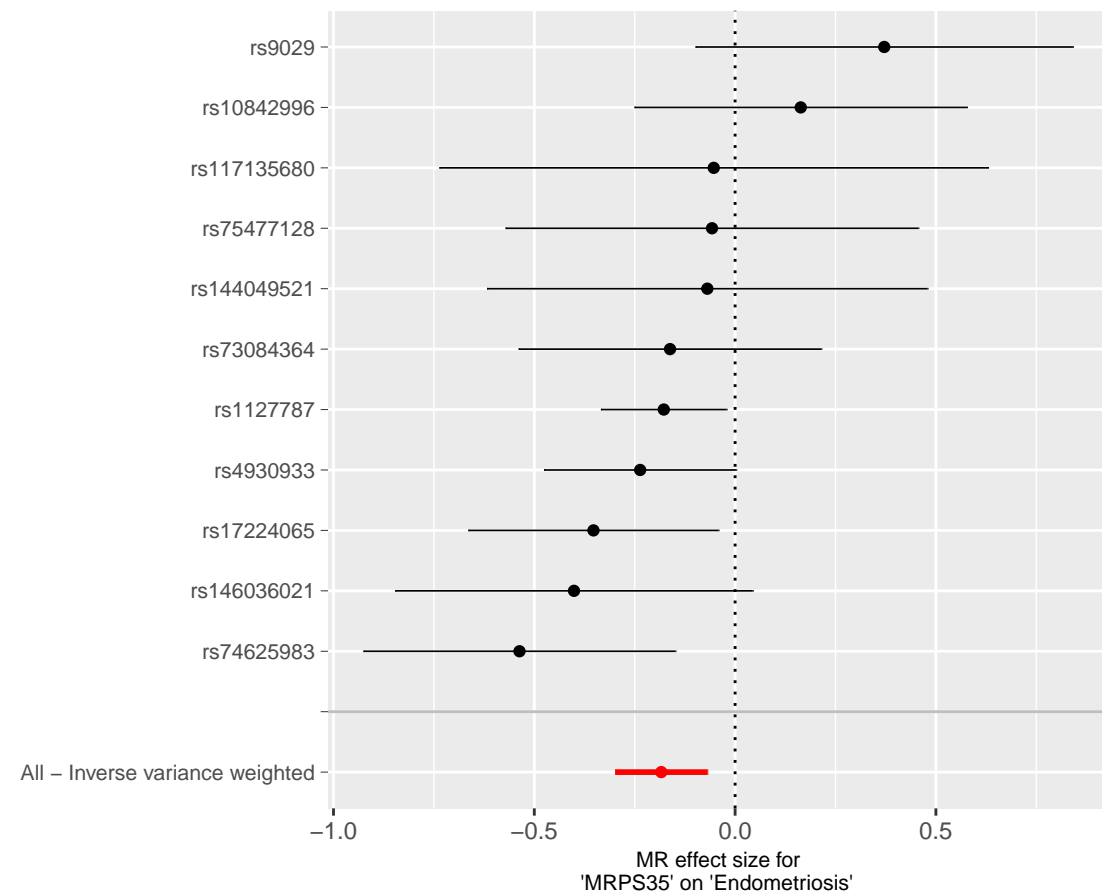

Supplement: Supplementary file 4 [file Datasheet4.zip › Supplementary documents1/eQTL_LOO/MRPS35_LOO_MR.pdf]

NARS2 – Leave-One-Out MR

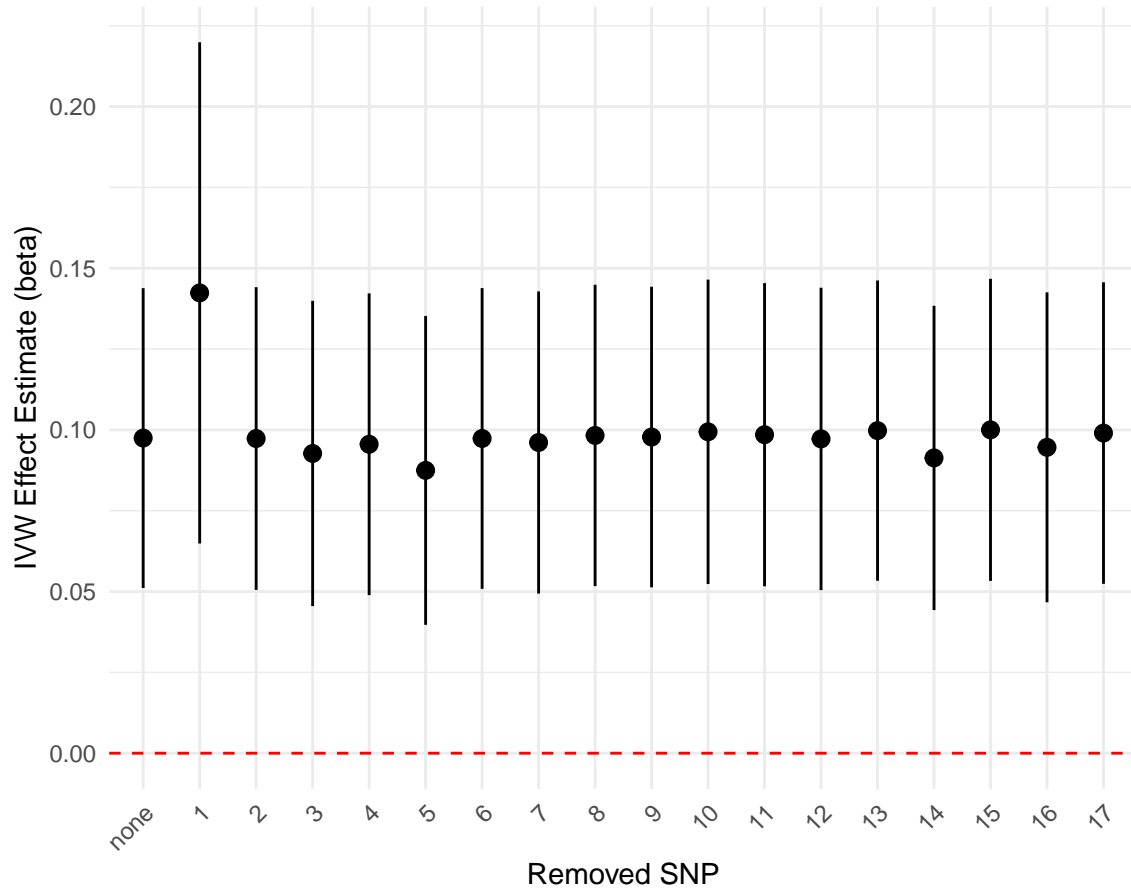

NARS2 – Forest Plot

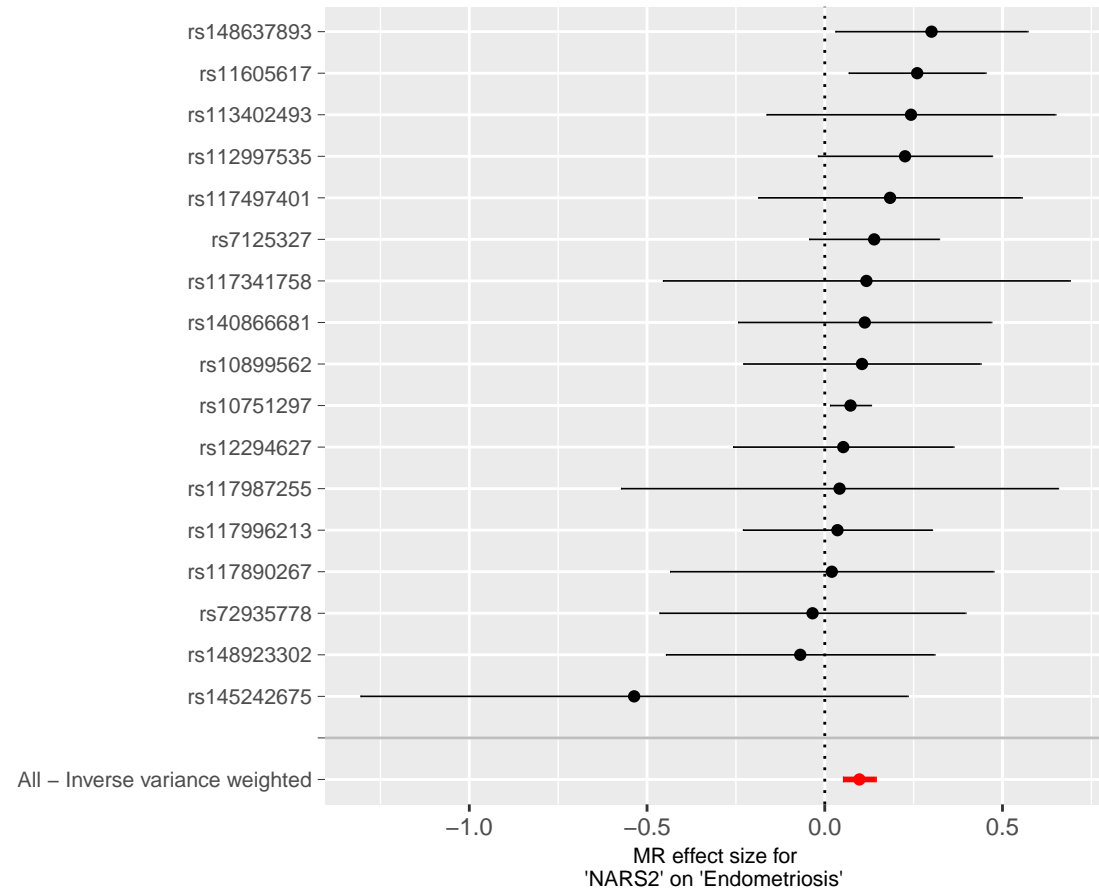

NARS2 – Funnel Plot

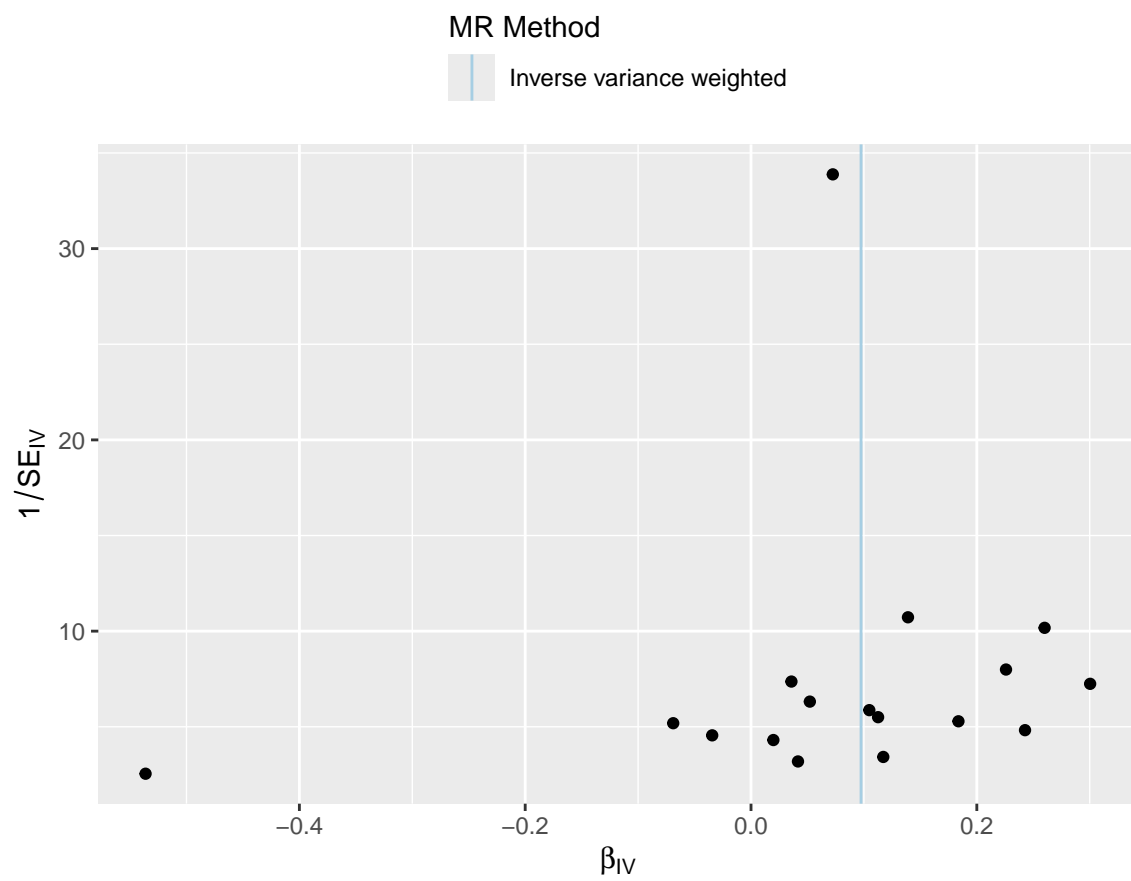

Supplement: Supplementary file 4 [file Datasheet4.zip › Supplementary documents1/eQTL_LOO/NARS2_LOO_MR.pdf]

NFU1 – Leave-One-Out MR

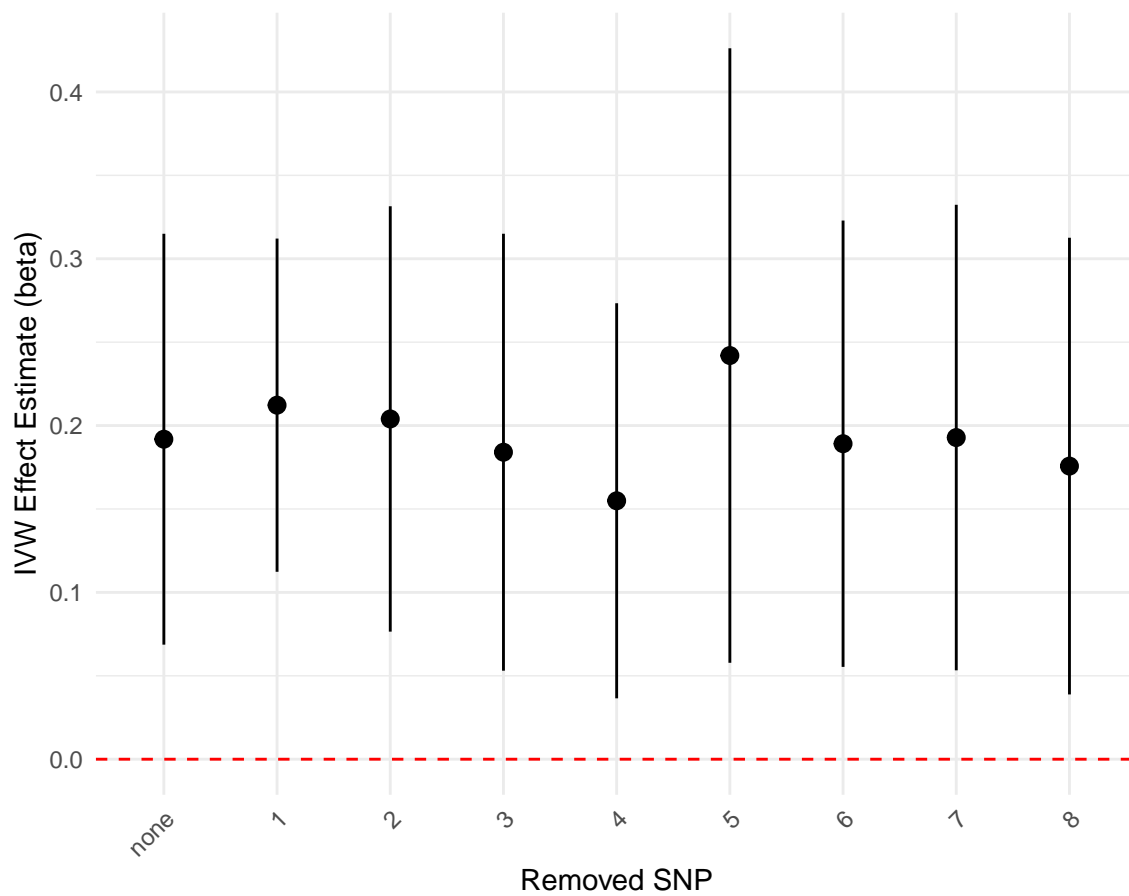

NFU1 – Forest Plot

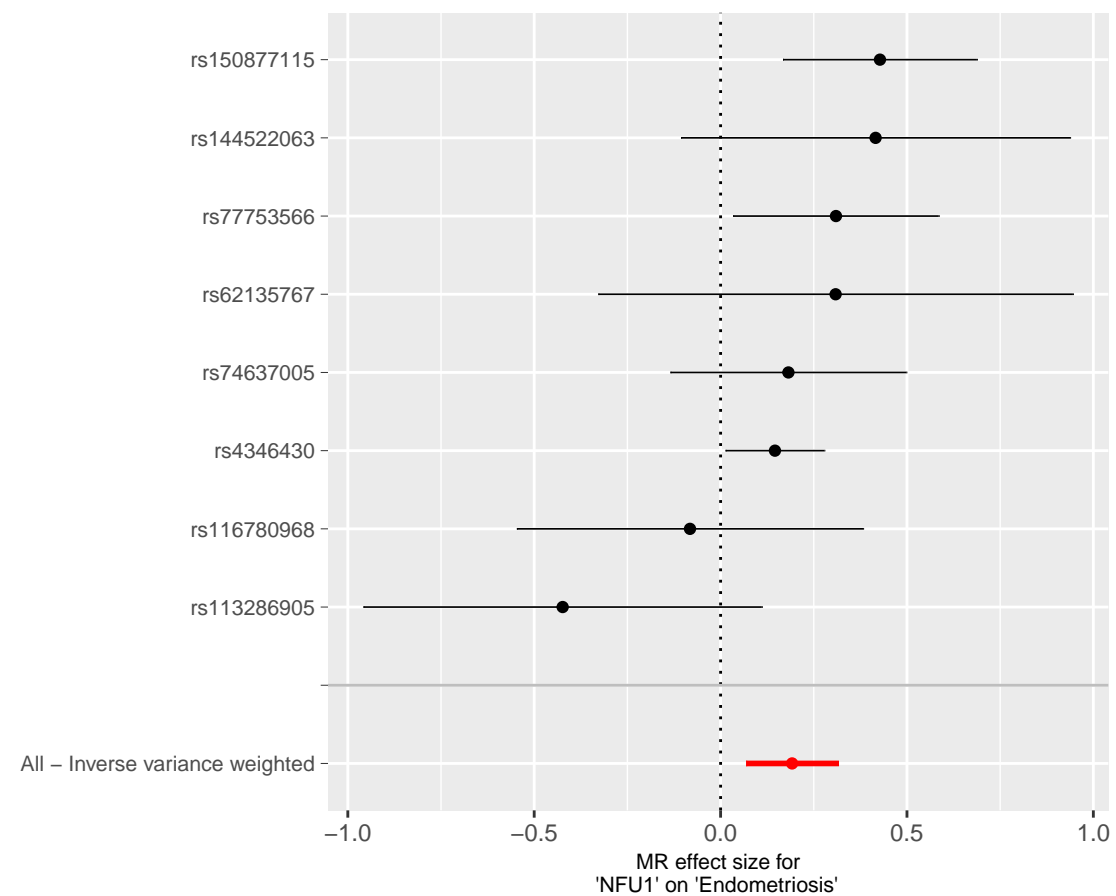

NFU1 – Funnel Plot

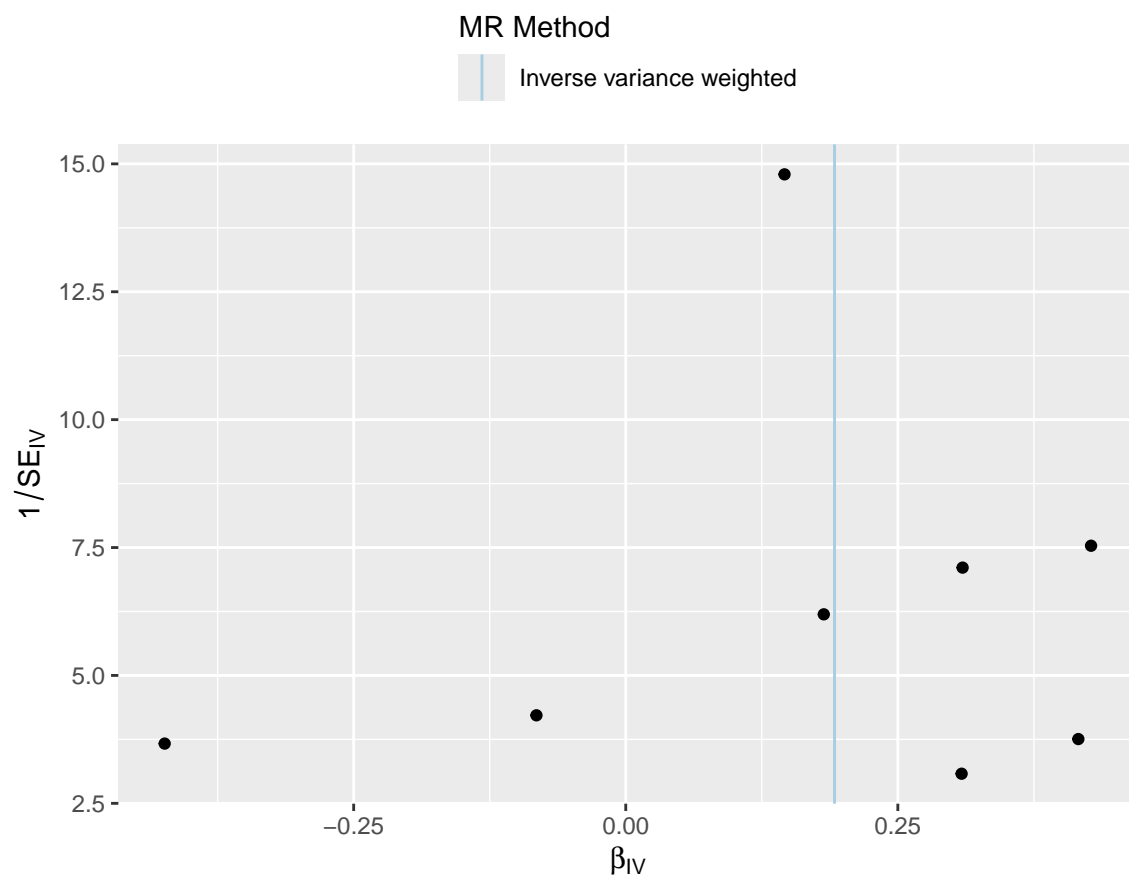

Supplement: Supplementary file 4 [file Datasheet4.zip › Supplementary documents1/eQTL_LOO/NFU1_LOO_MR.pdf]

NME4 – Leave-One-Out MR

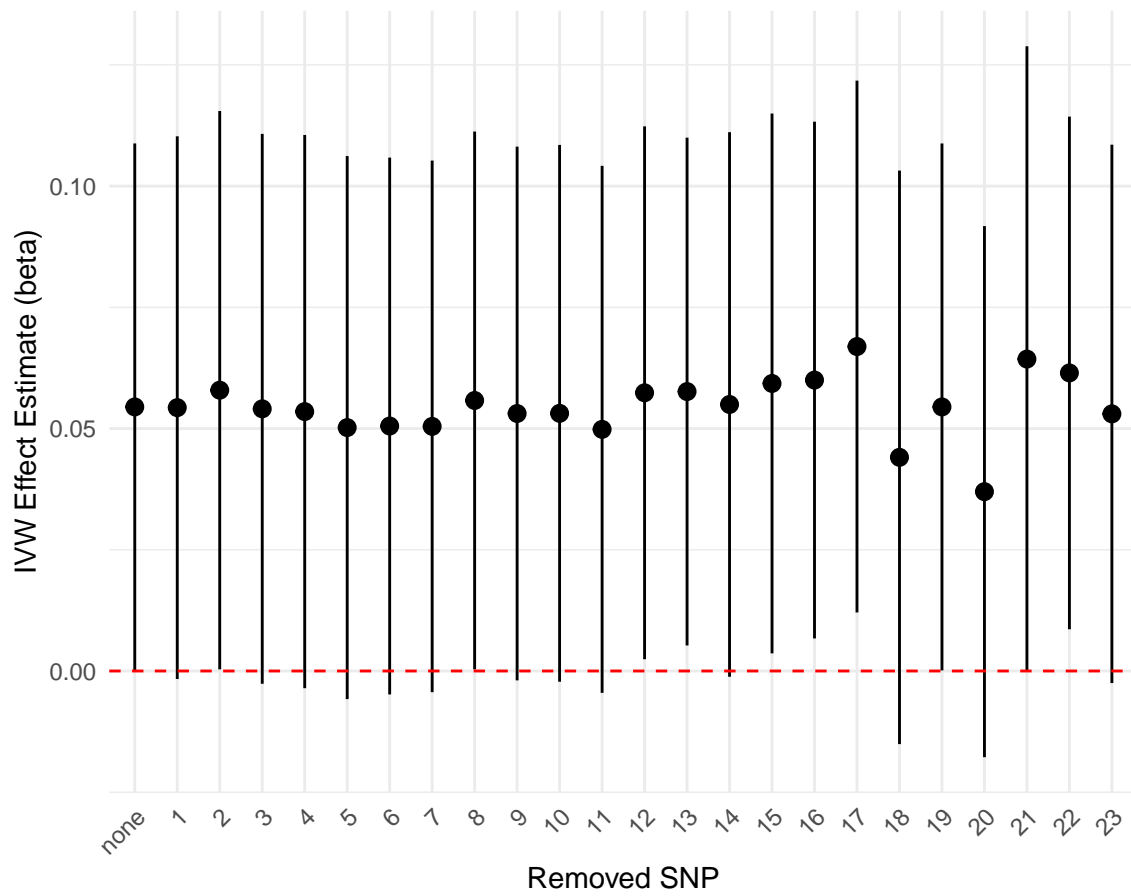

NME4 – Funnel Plot

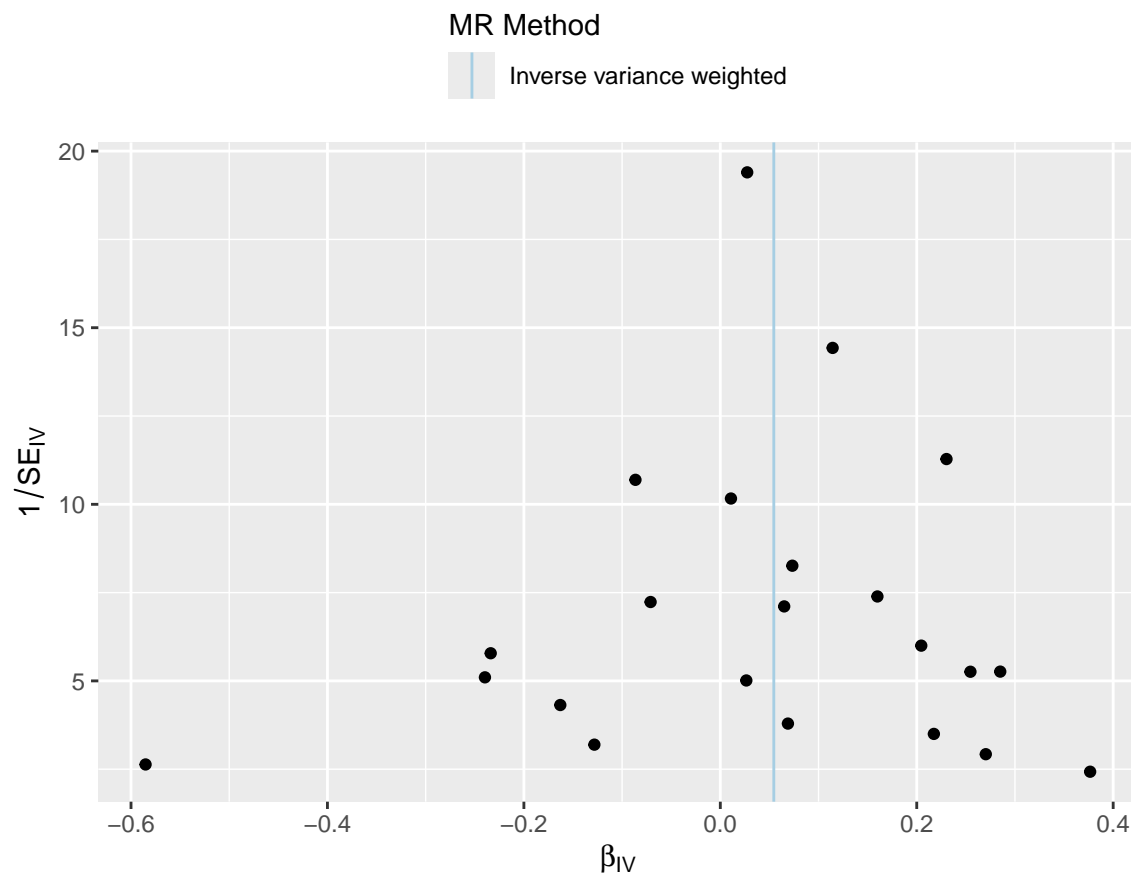

NME4 – Forest Plot

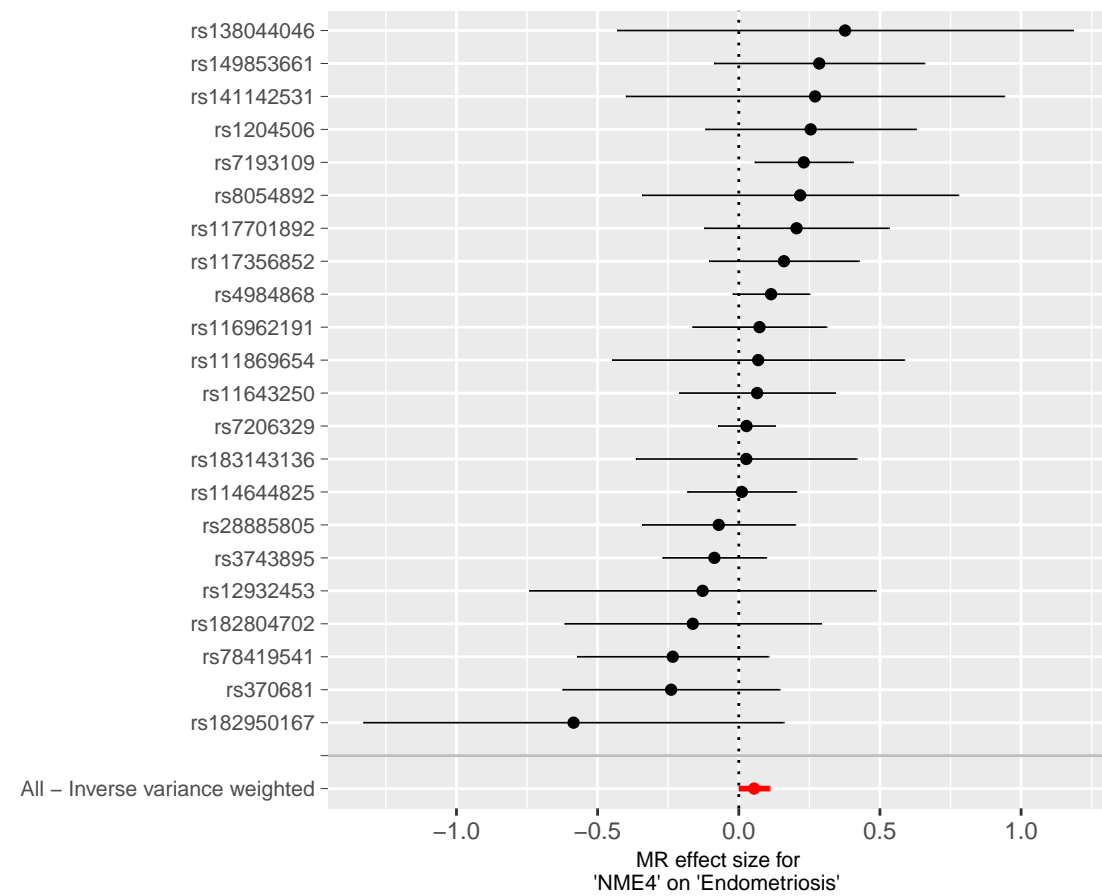

Supplement: Supplementary file 4 [file Datasheet4.zip › Supplementary documents1/eQTL_LOO/NME4_LOO_MR.pdf]

SLC25A29 – Leave-One-Out MR

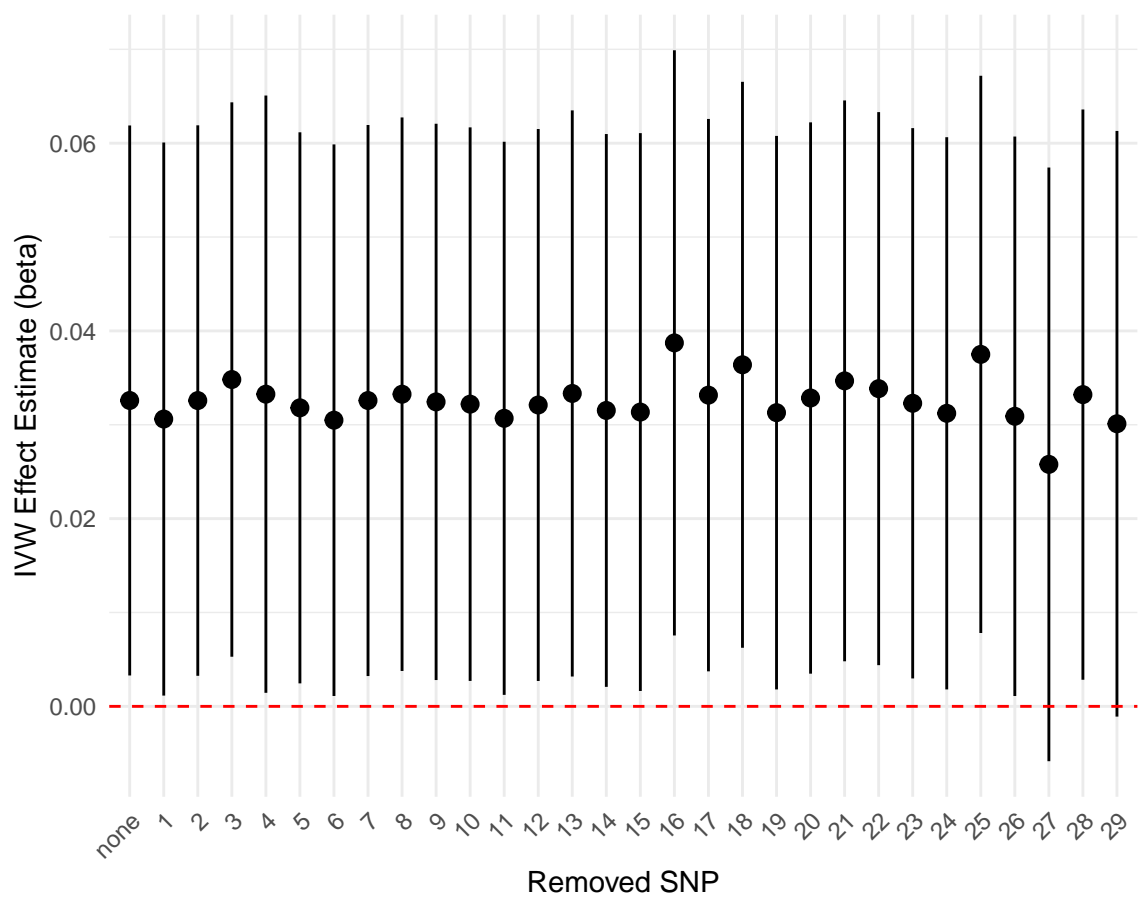

SLC25A29 – Forest Plot

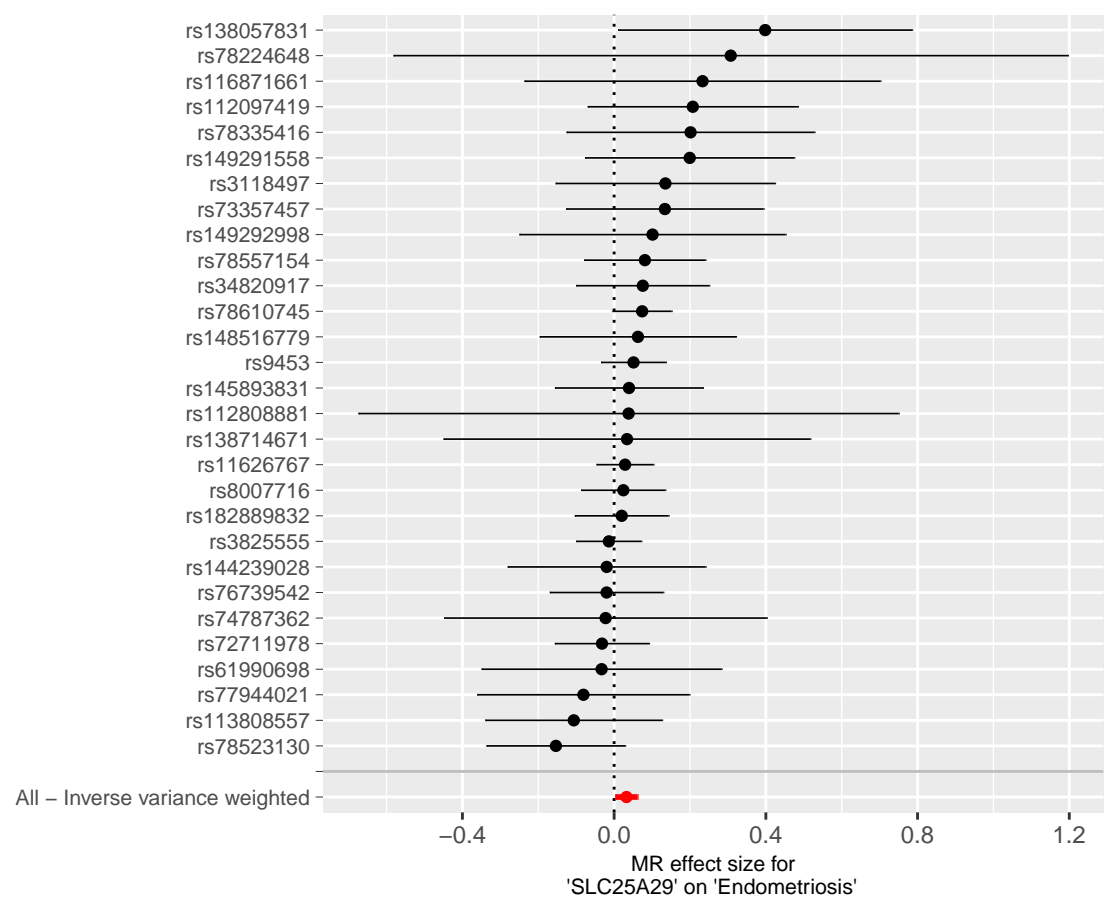

SLC25A29 – Funnel Plot

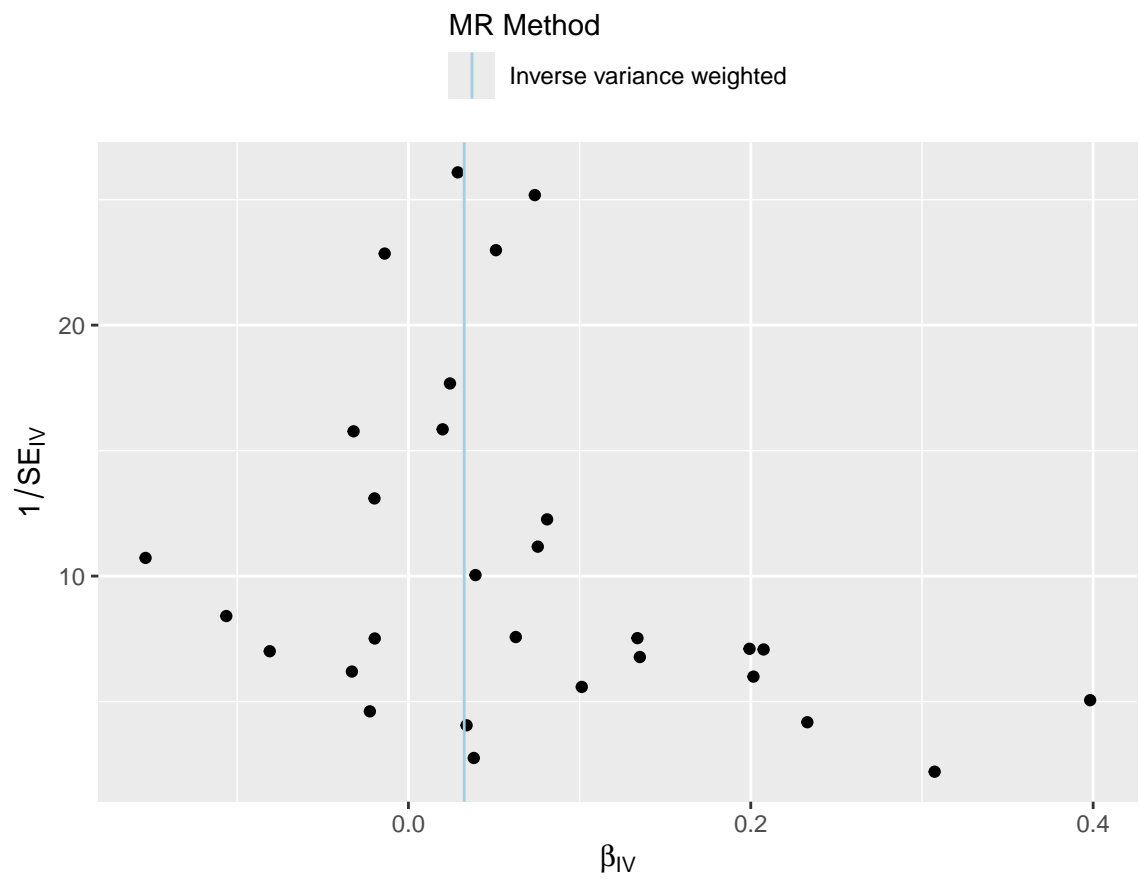

Supplement: Supplementary file 4 [file Datasheet4.zip › Supplementary documents1/eQTL_LOO/SLC25A29_LOO_MR.pdf]

UNG – Leave-One-Out MR

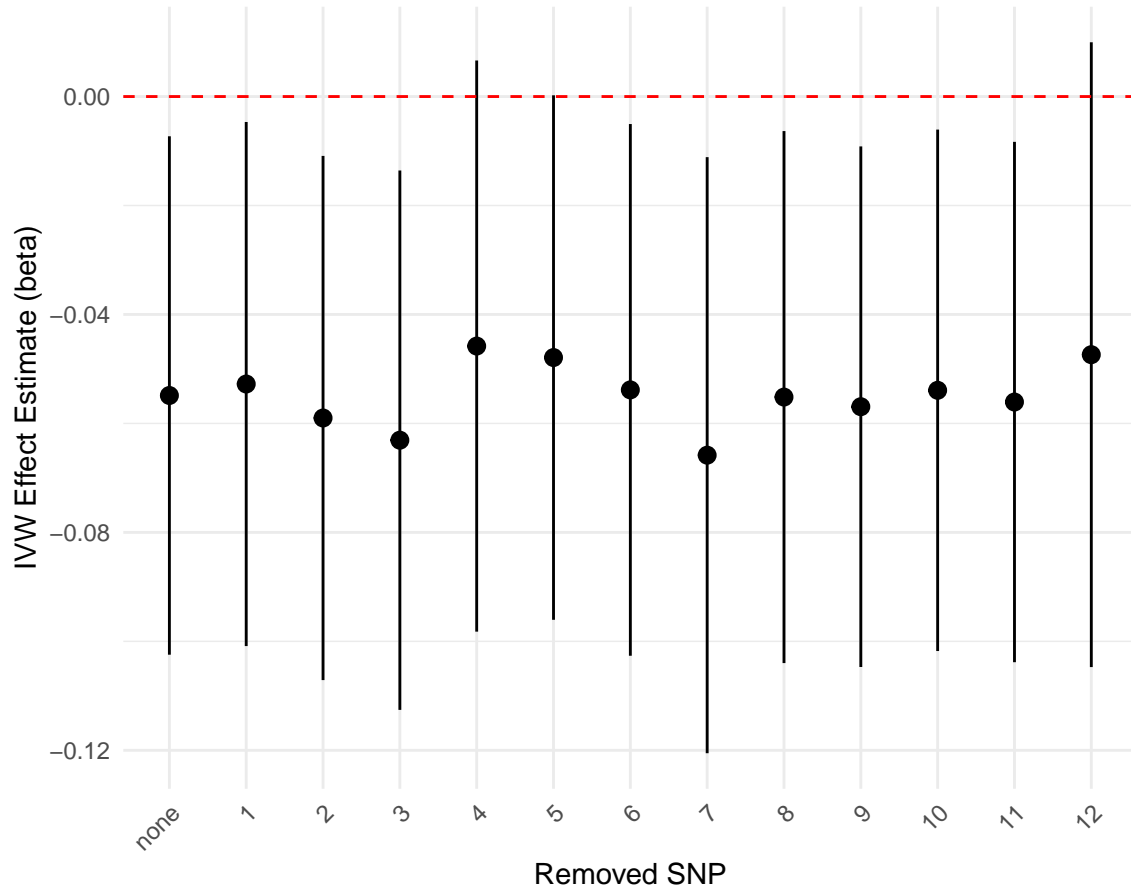

UNG – Forest Plot

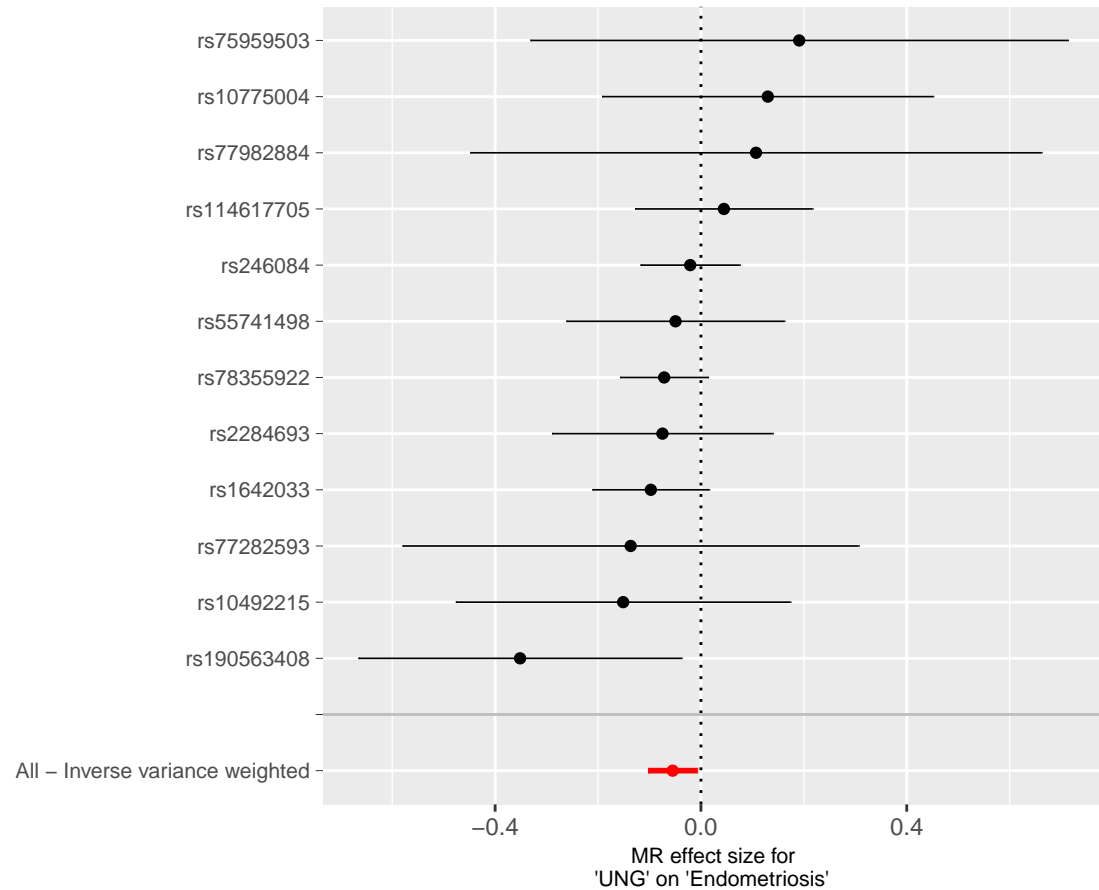

UNG – Funnel Plot

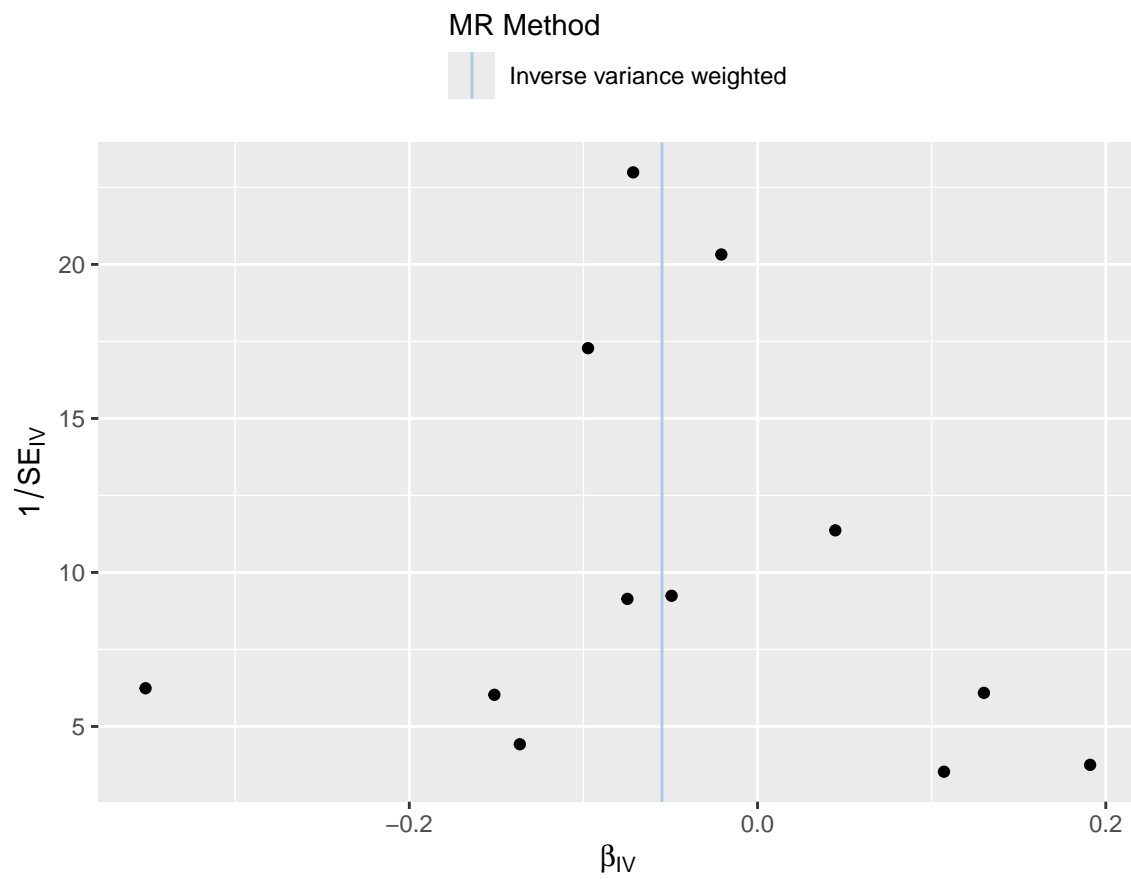

Supplement: Supplementary file 4 [file Datasheet4.zip › Supplementary documents1/eQTL_LOO/UNG_LOO_MR.pdf]

cg00432937 – Leave-One-Out MR

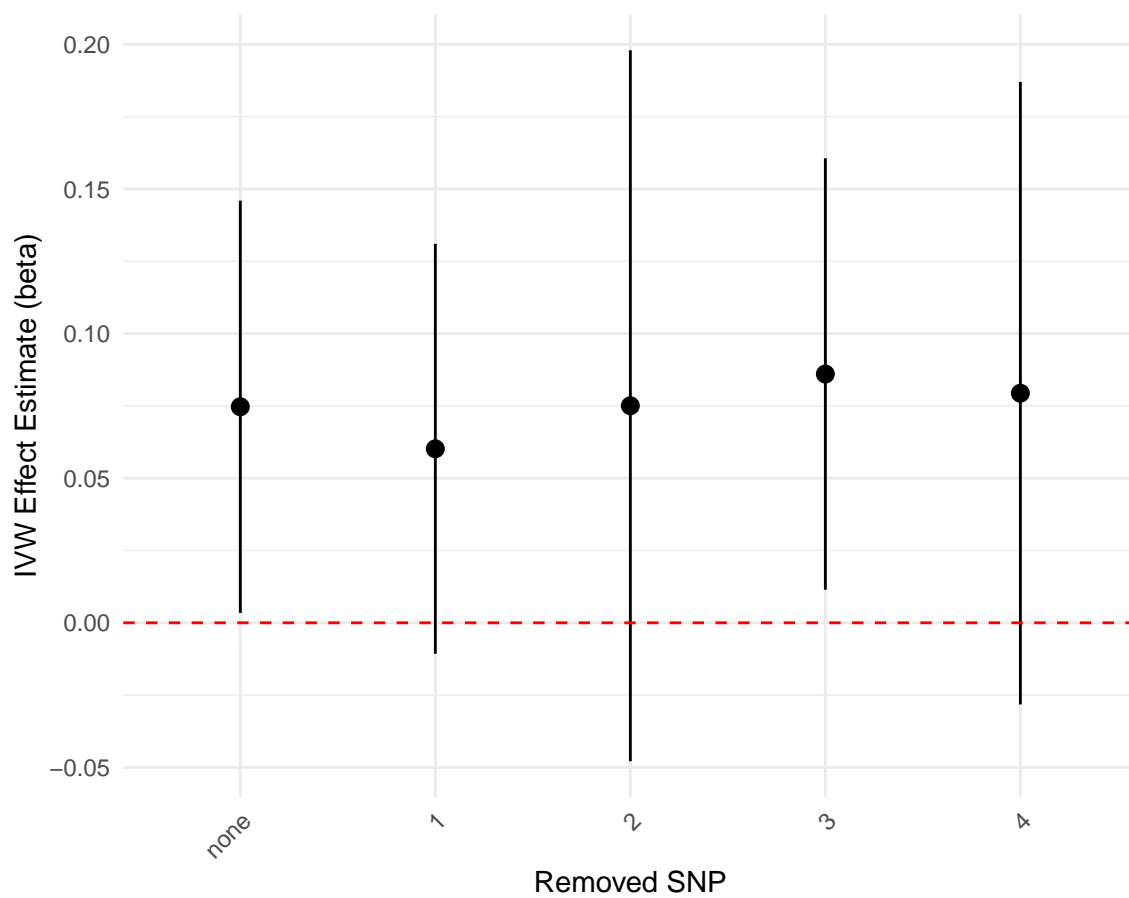

cg00432937 – Forest Plot

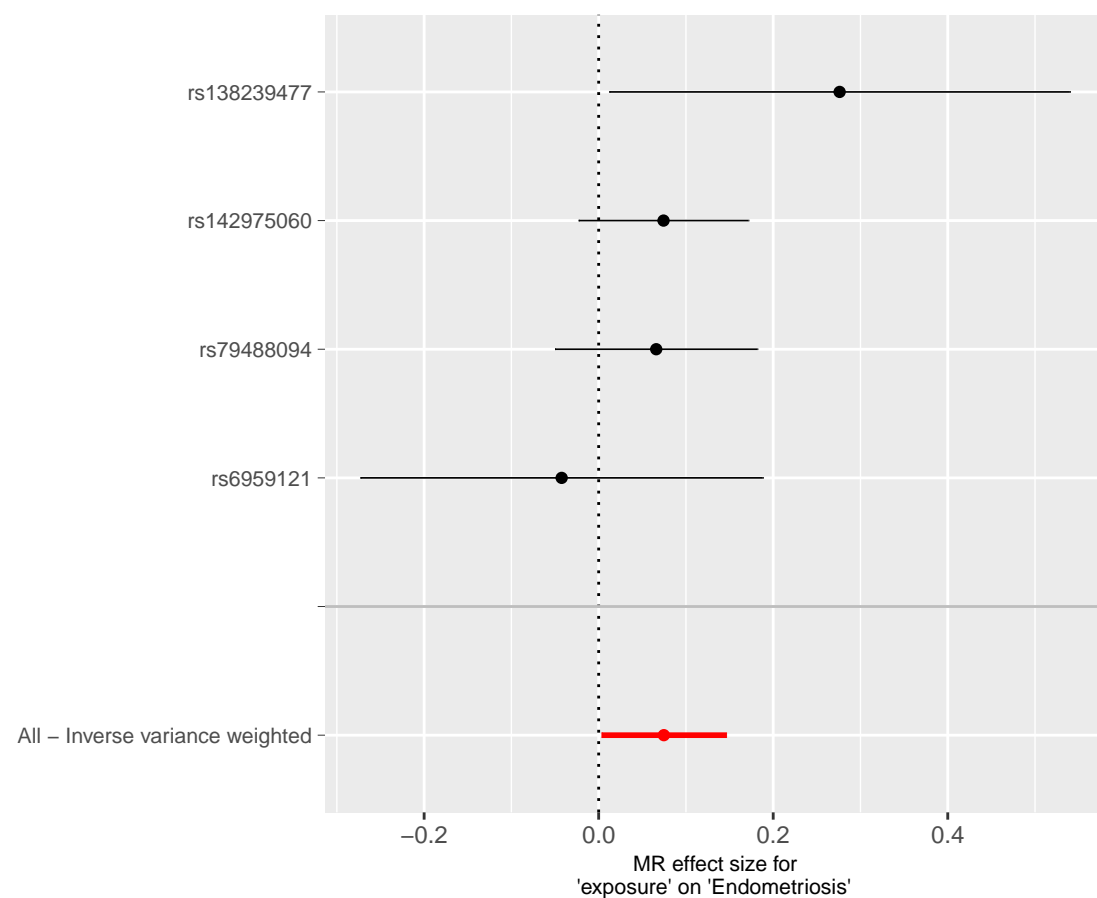

cg00432937 – Funnel Plot

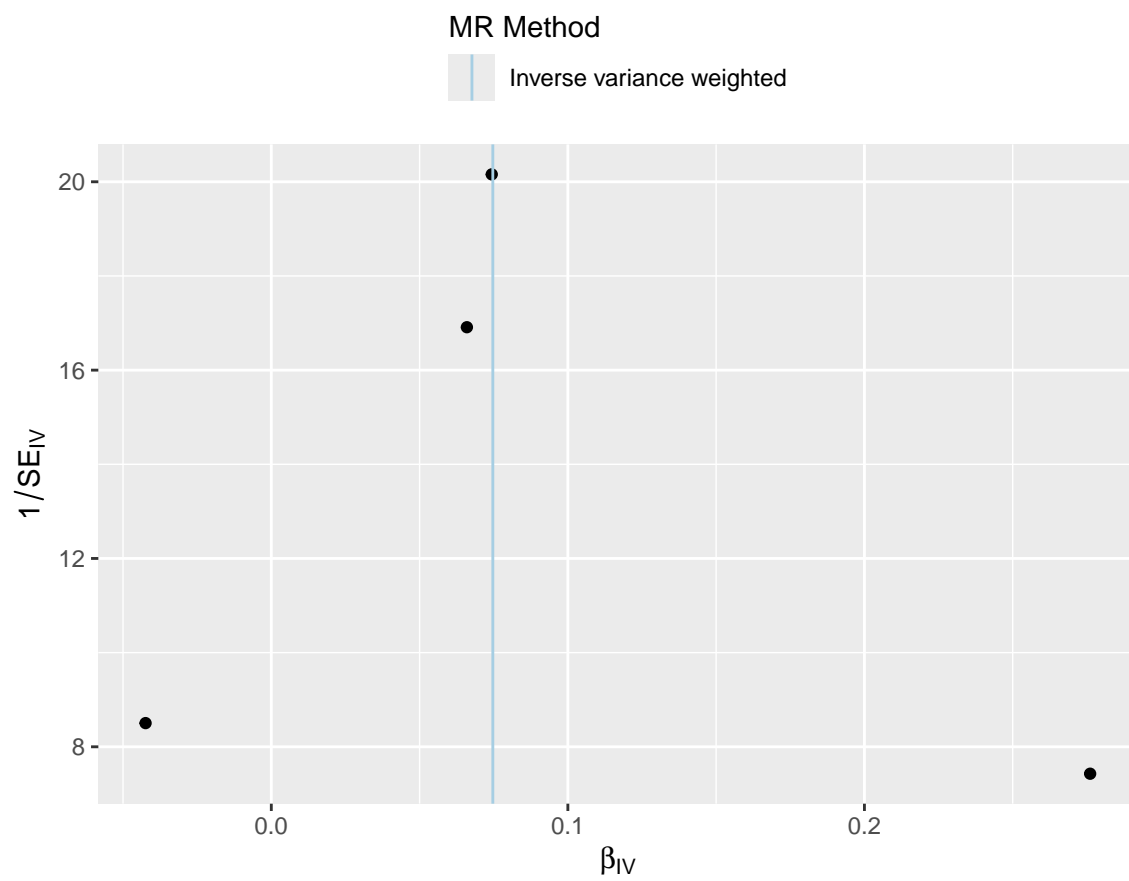

Supplement: Supplementary file 5 [file Datasheet5.zip › Supplementary documents3/mQTL_LOO/cg00432937_LOO_MR.pdf]

cg02049041 – Leave-One-Out MR

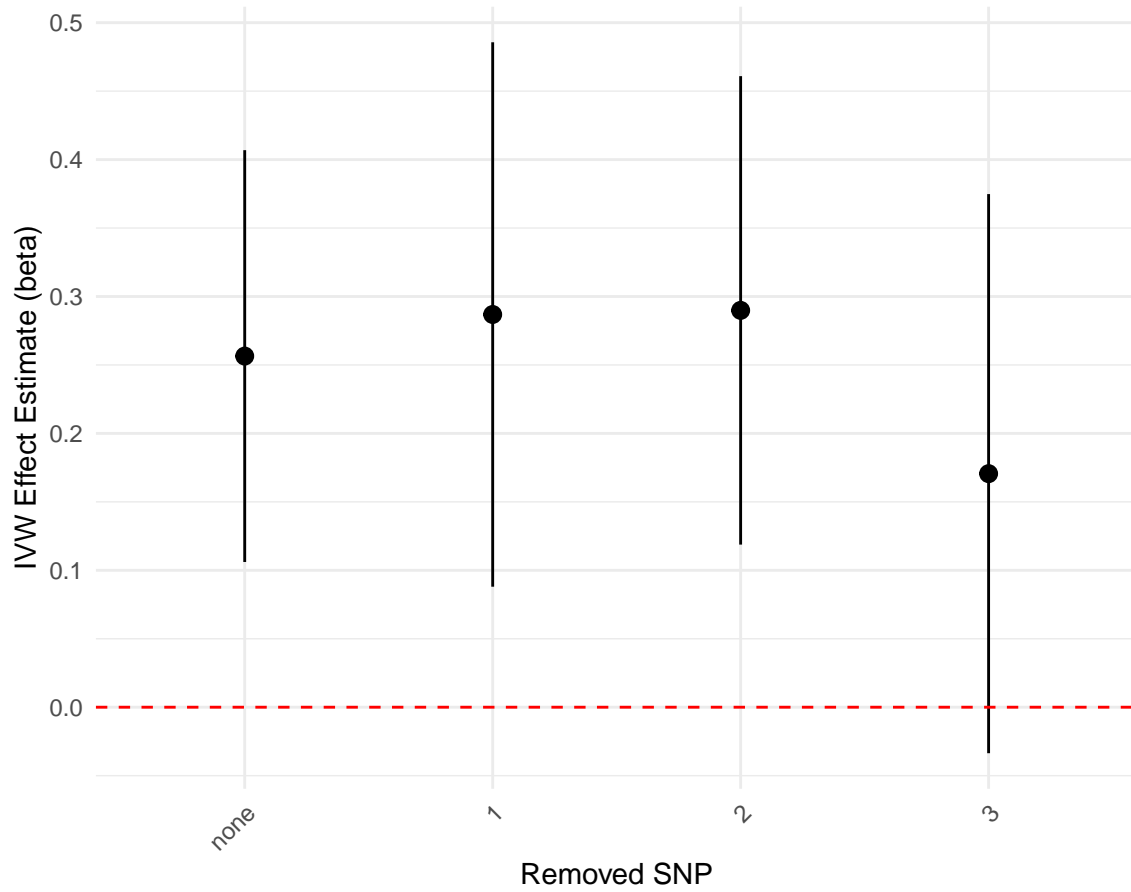

cg02049041 – Forest Plot

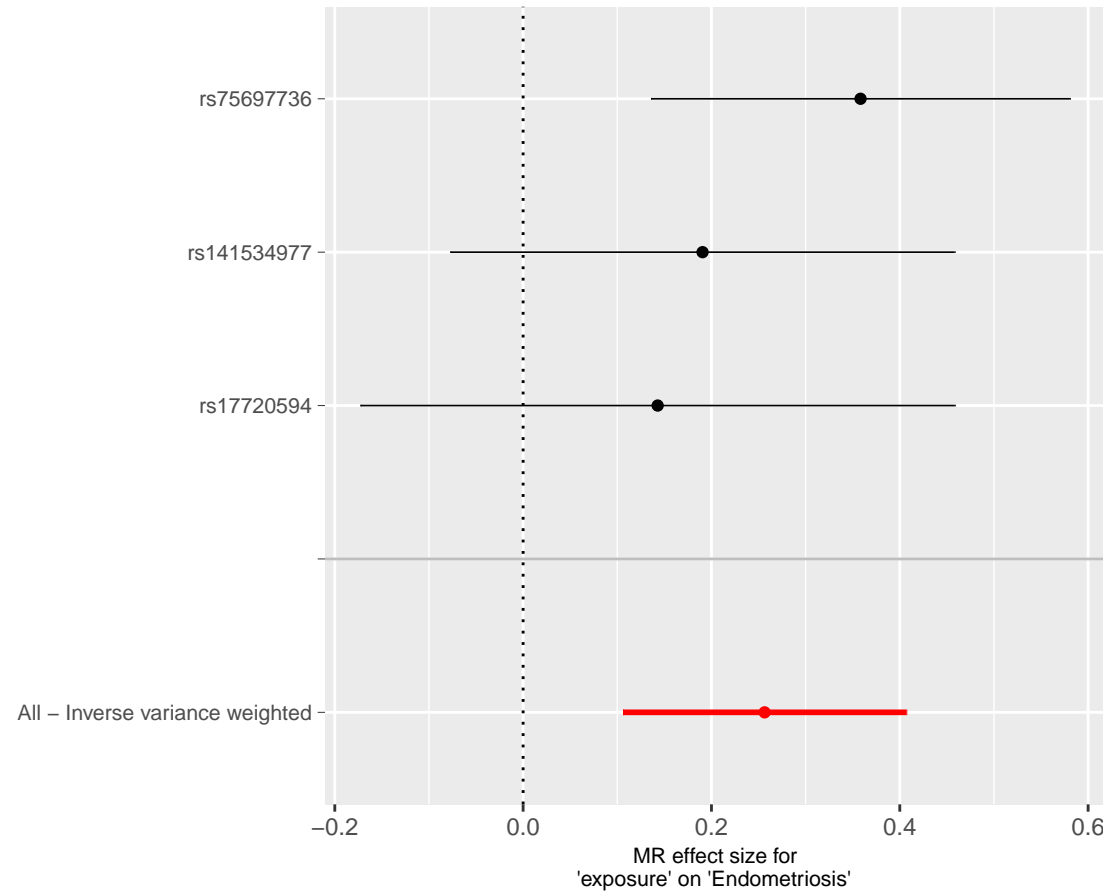

cg02049041 – Funnel Plot

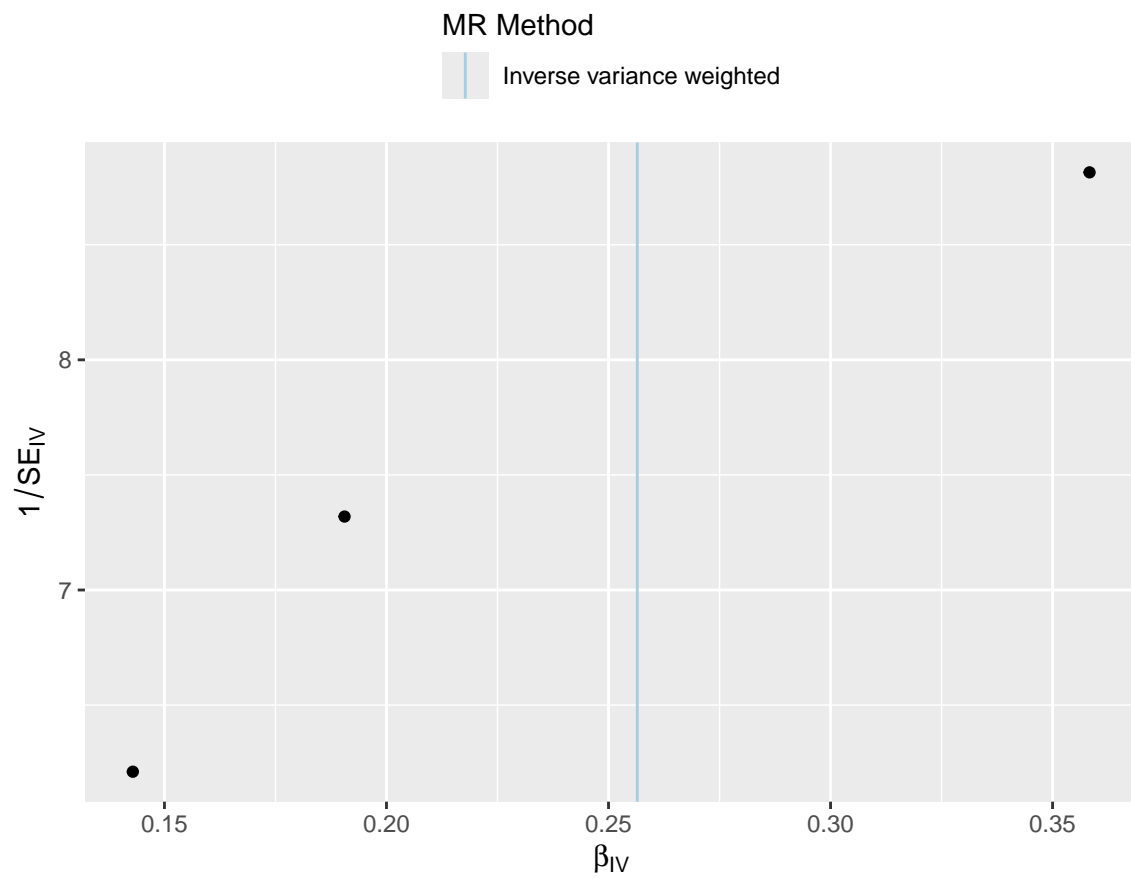

Supplement: Supplementary file 5 [file Datasheet5.zip › Supplementary documents3/mQTL_LOO/cg02049041_LOO_MR.pdf]

cg02753203 – Leave-One-Out MR

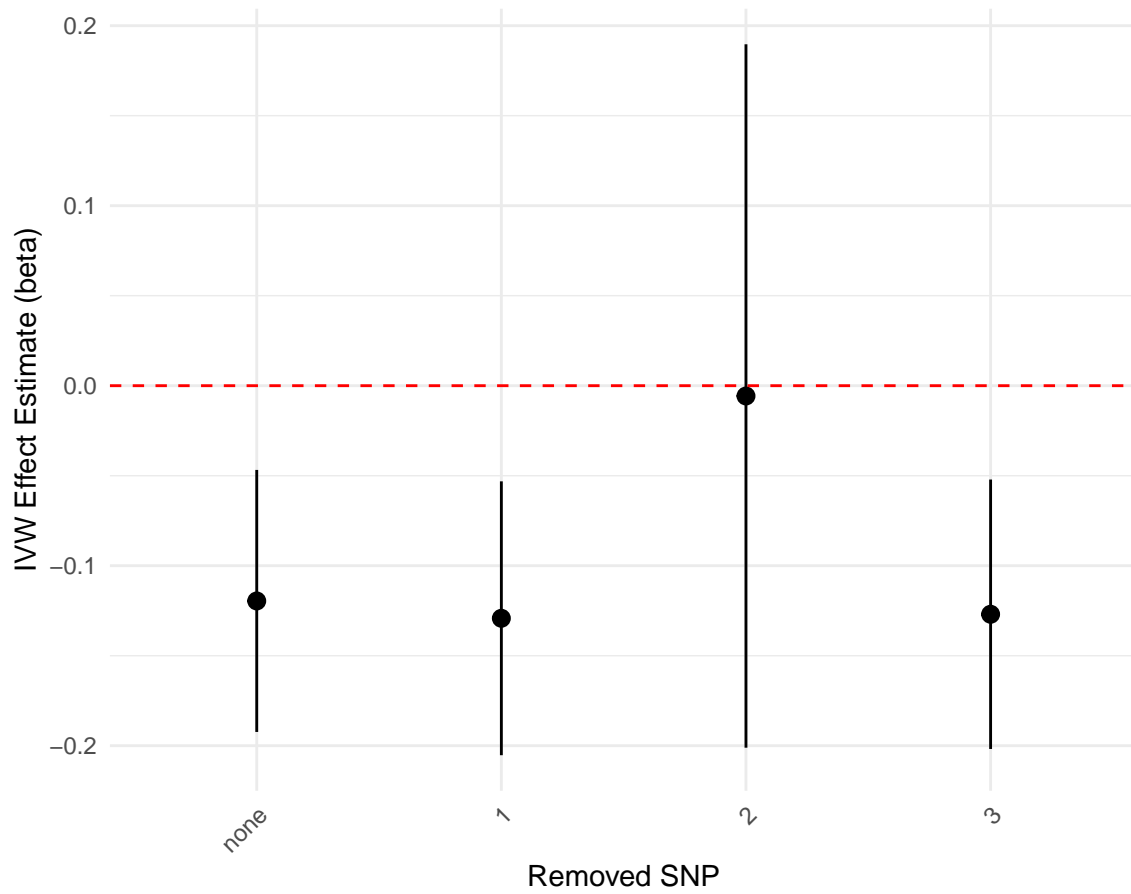

cg02753203 – Forest Plot

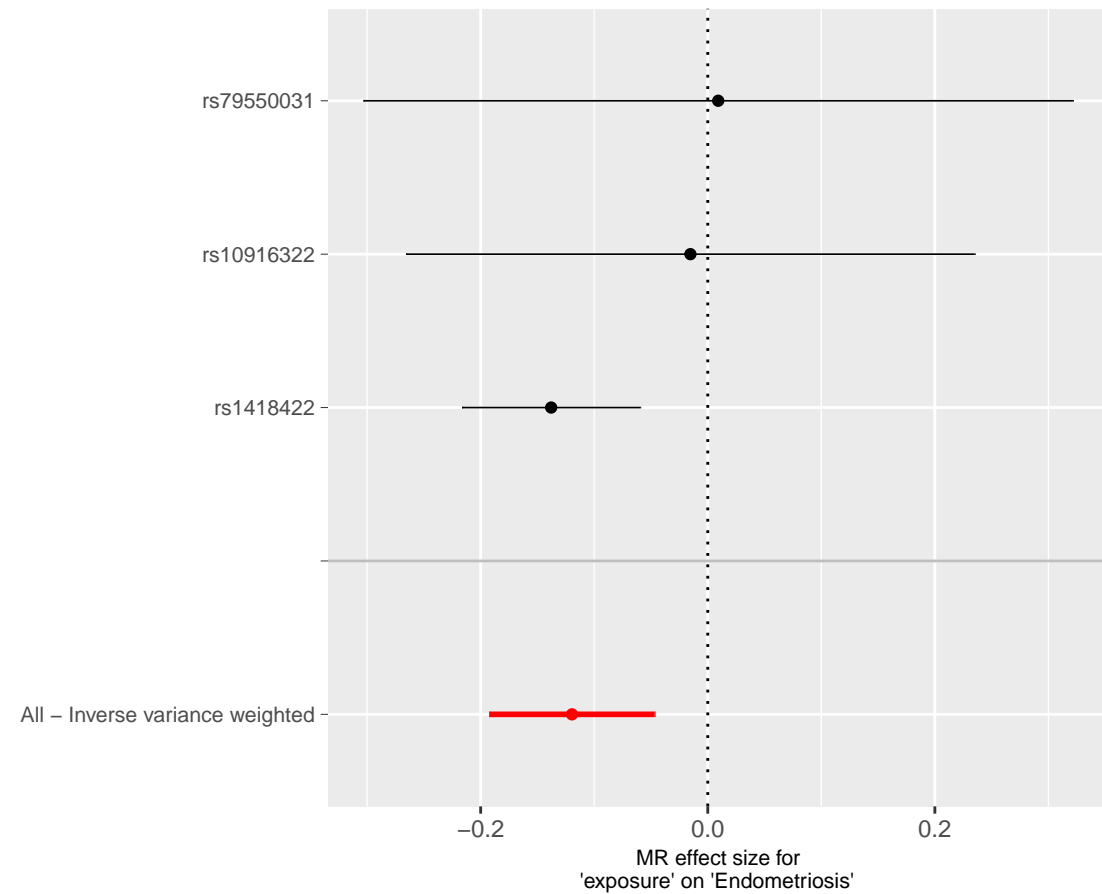

cg02753203 – Funnel Plot

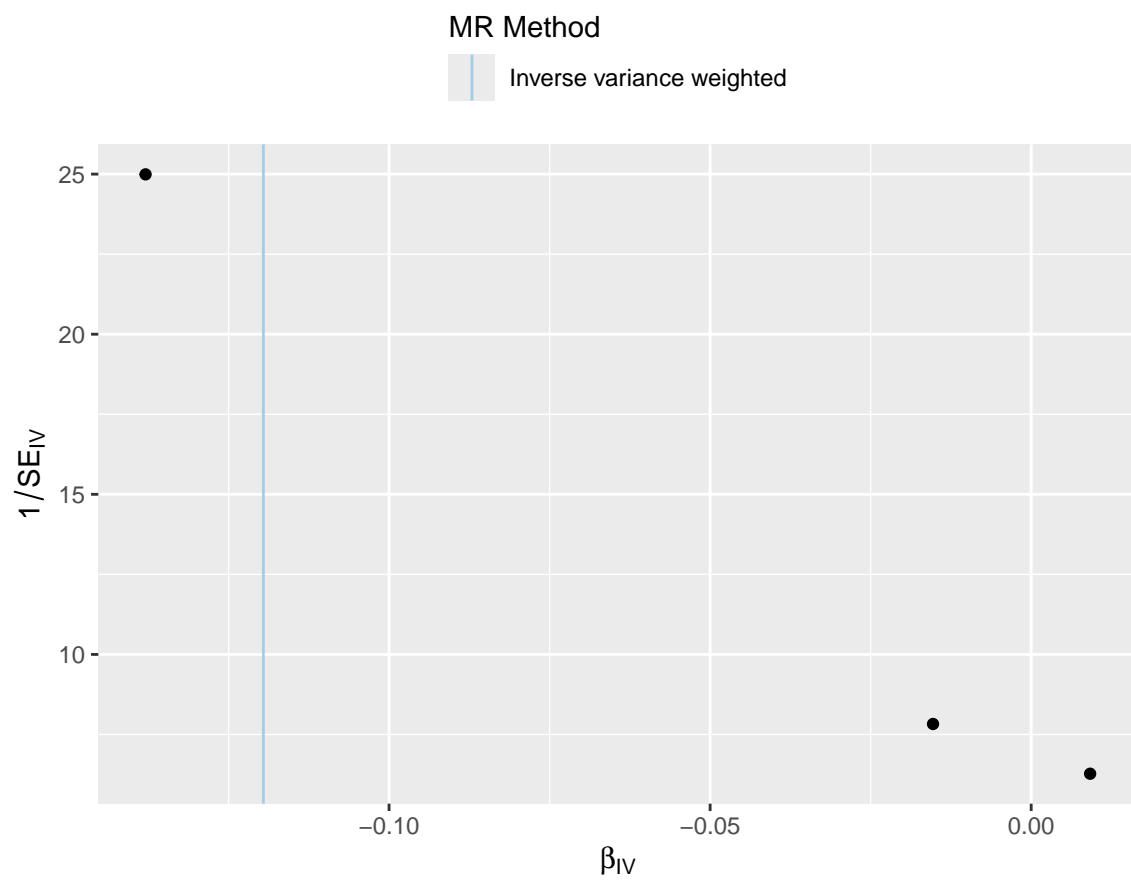

Supplement: Supplementary file 5 [file Datasheet5.zip › Supplementary documents3/mQTL_LOO/cg02753203_LOO_MR.pdf]

cg05987564 – Leave-One-Out MR

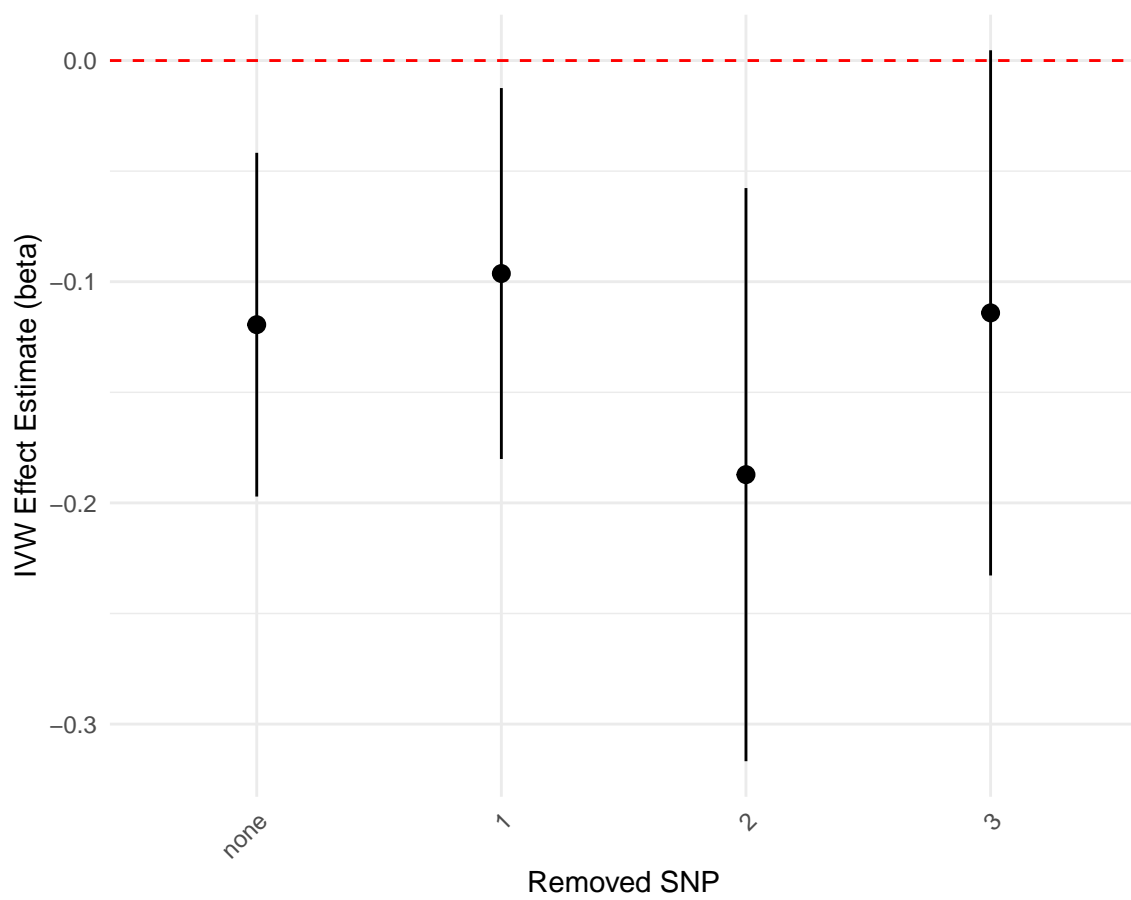

cg05987564 – Forest Plot

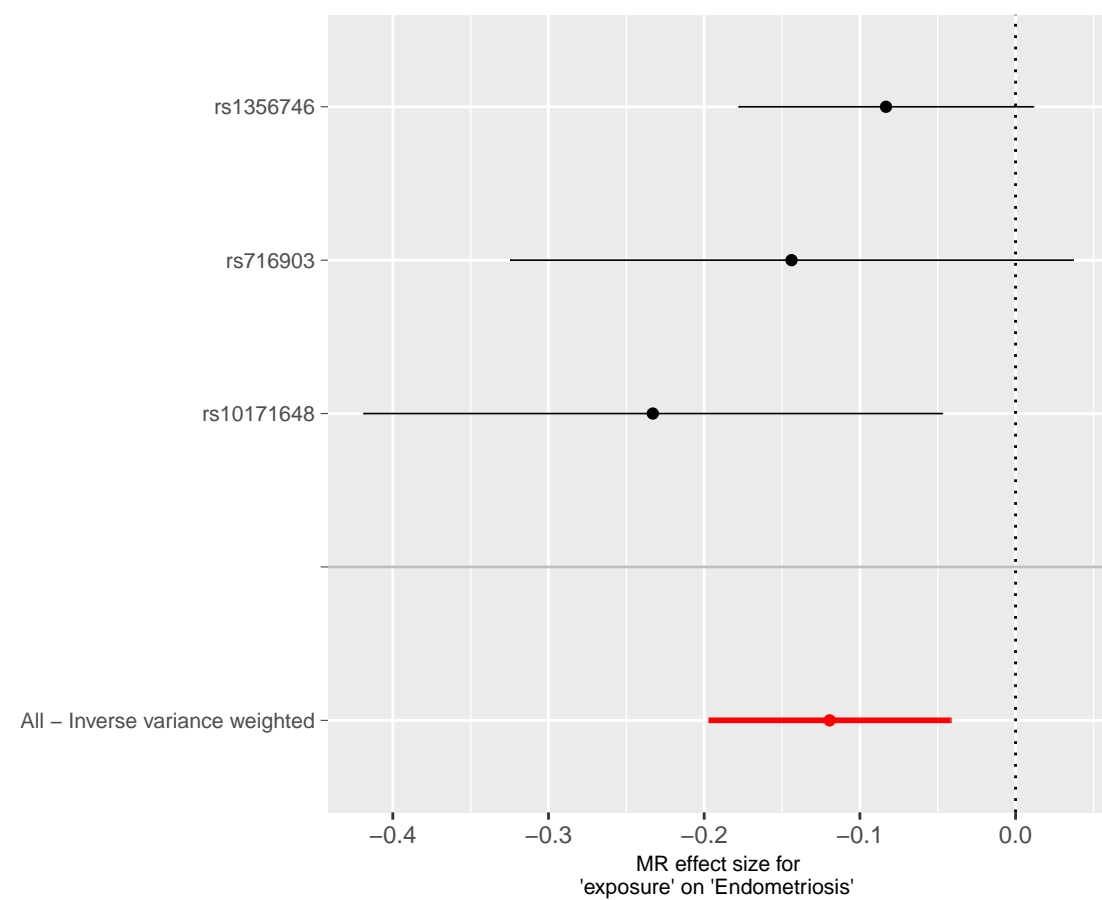

cg05987564 – Funnel Plot

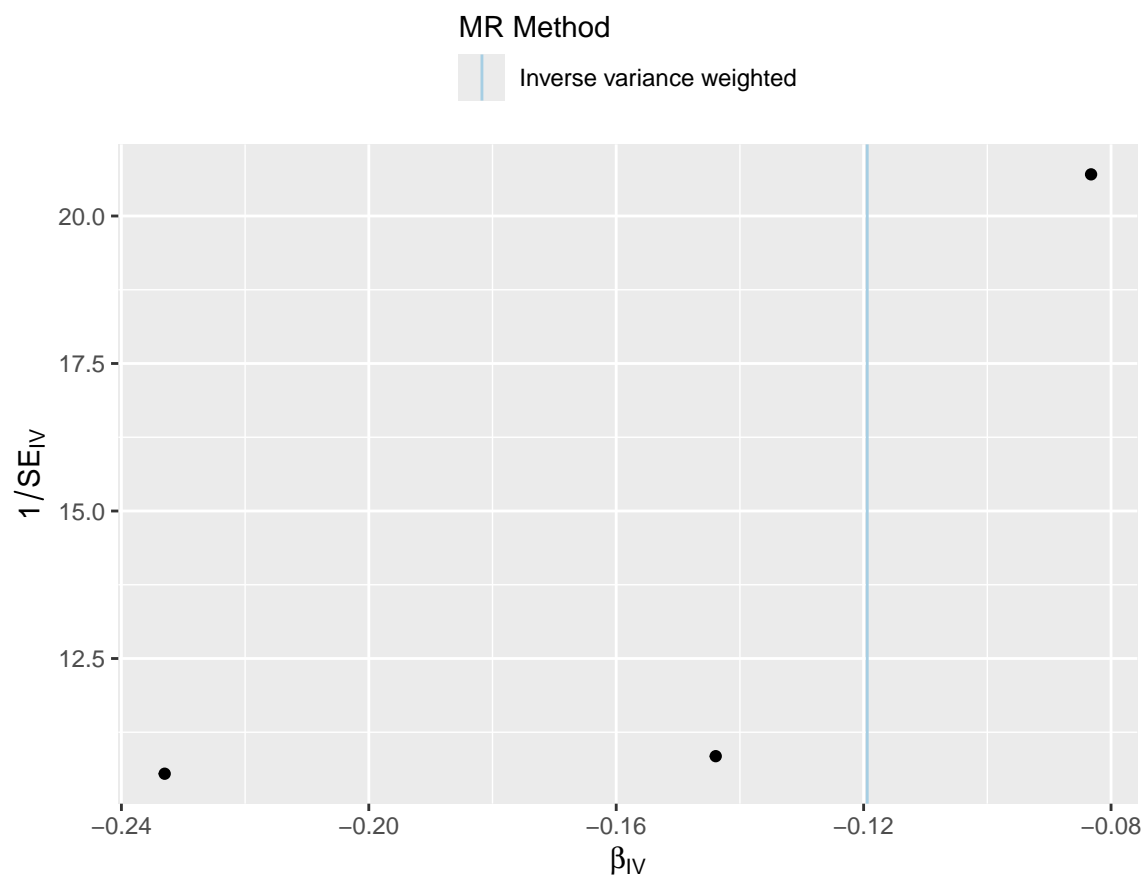

Supplement: Supplementary file 5 [file Datasheet5.zip › Supplementary documents3/mQTL_LOO/cg05987564_LOO_MR.pdf]

cg06183267 – Leave-One-Out MR

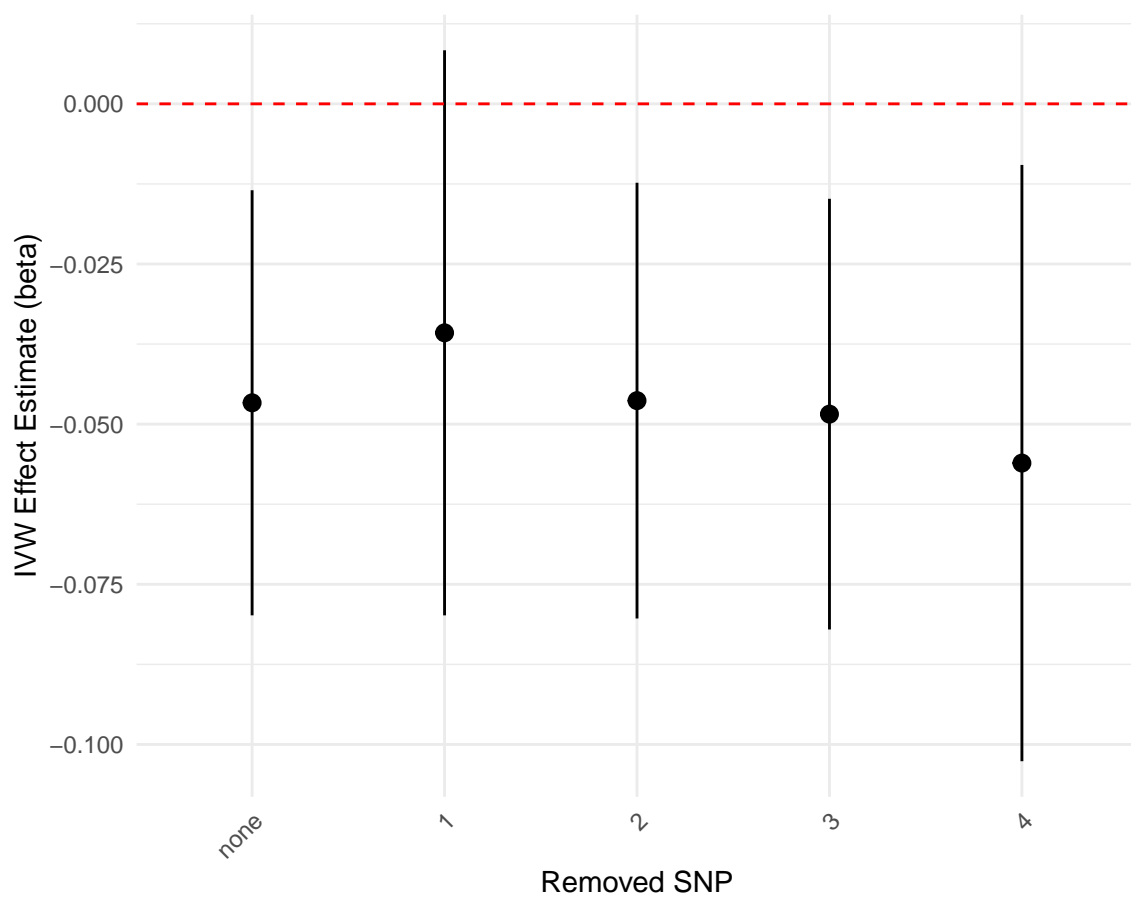

cg06183267 – Forest Plot

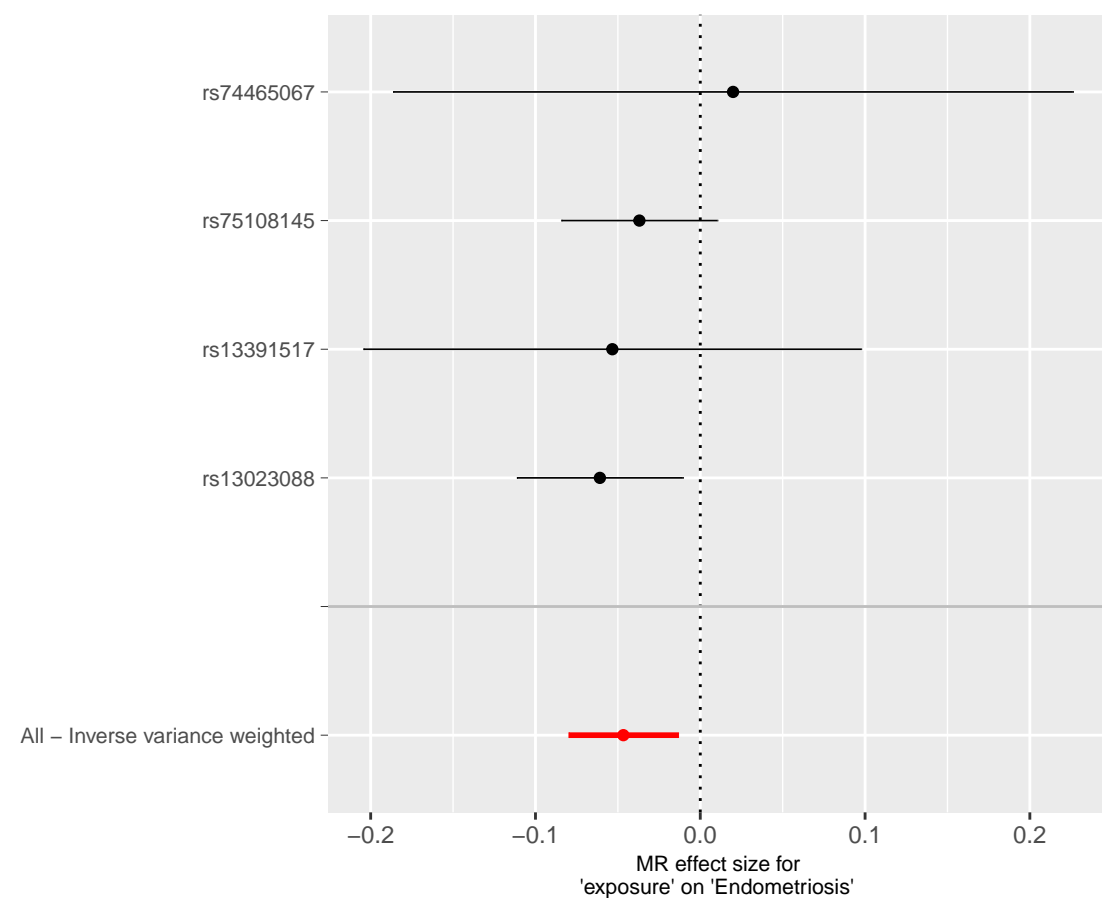

cg06183267 – Funnel Plot

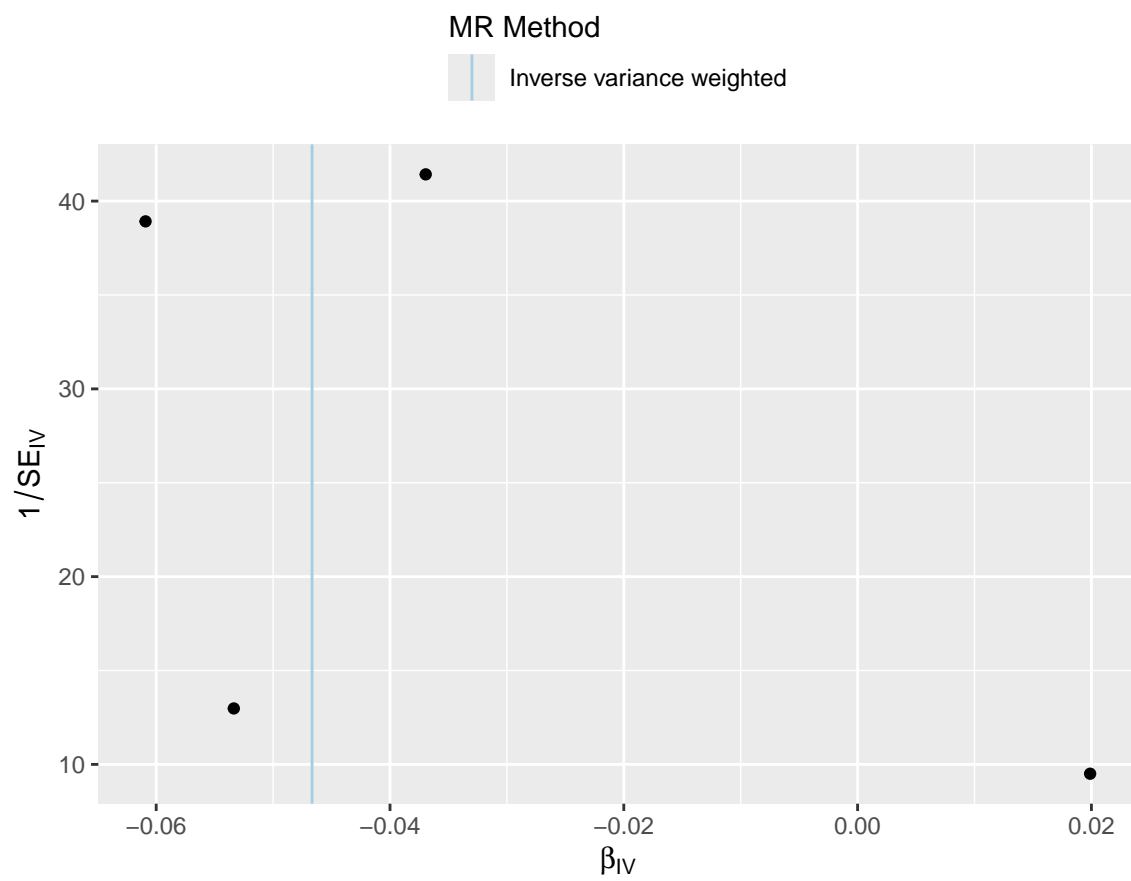

Supplement: Supplementary file 5 [file Datasheet5.zip › Supplementary documents3/mQTL_LOO/cg06183267_LOO_MR.pdf]

cg06365623 – Leave-One-Out MR

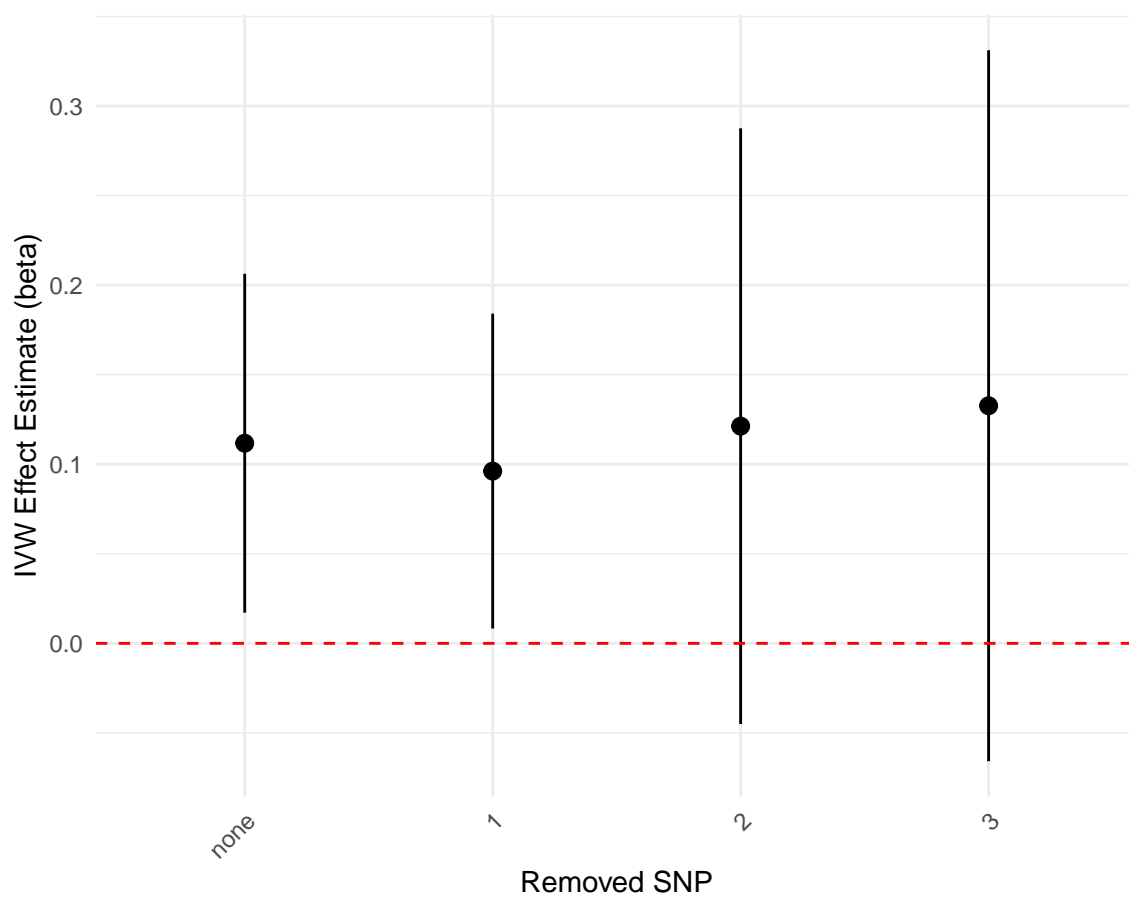

cg06365623 – Funnel Plot

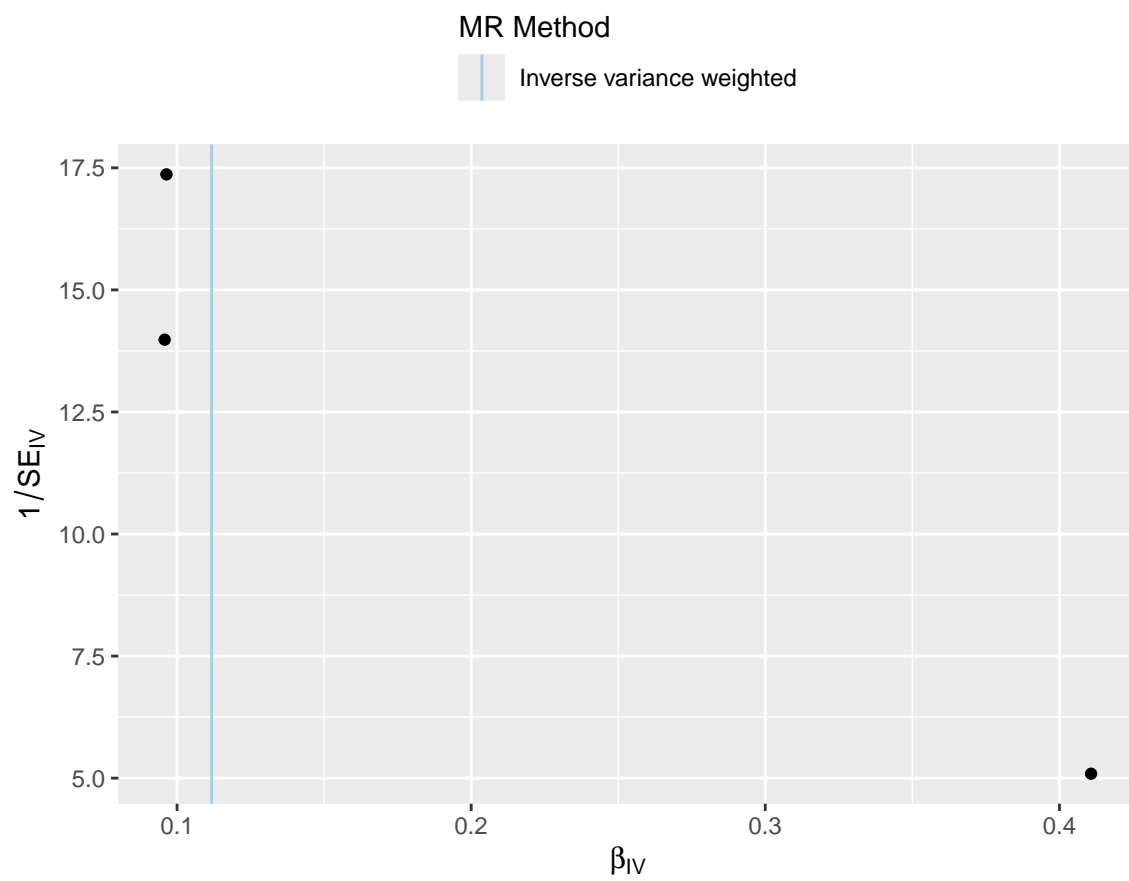

cg06365623 – Forest Plot

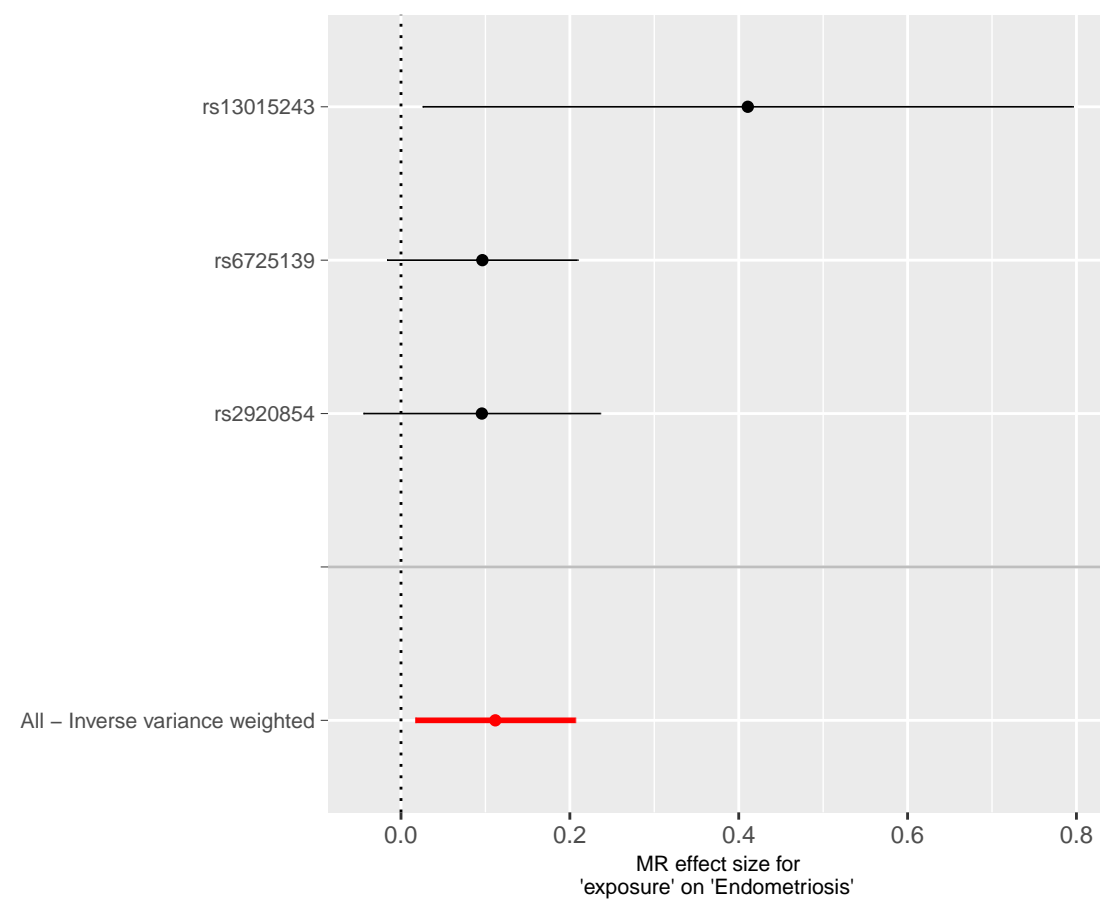

Supplement: Supplementary file 5 [file Datasheet5.zip › Supplementary documents3/mQTL_LOO/cg06365623_LOO_MR.pdf]

cg06380072 – Leave-One-Out MR

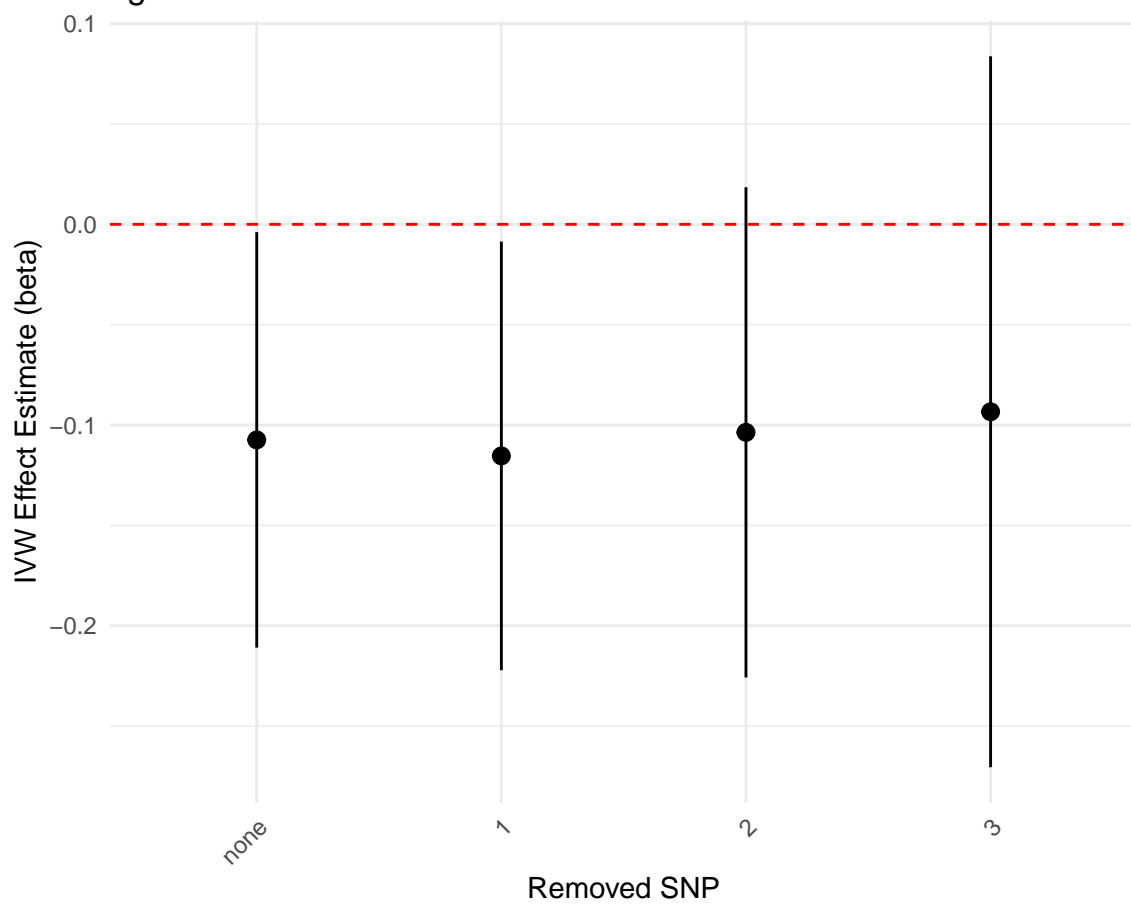

cg06380072 – Forest Plot

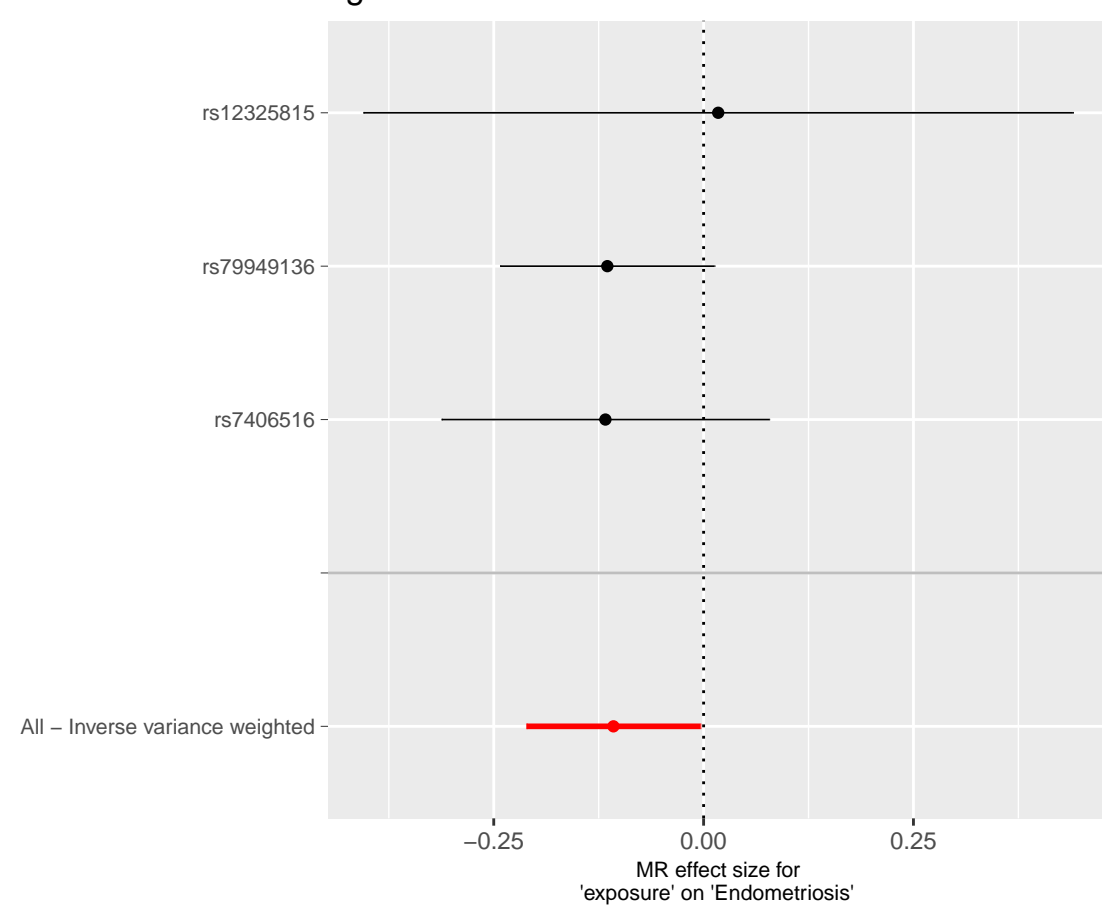

cg06380072 – Funnel Plot

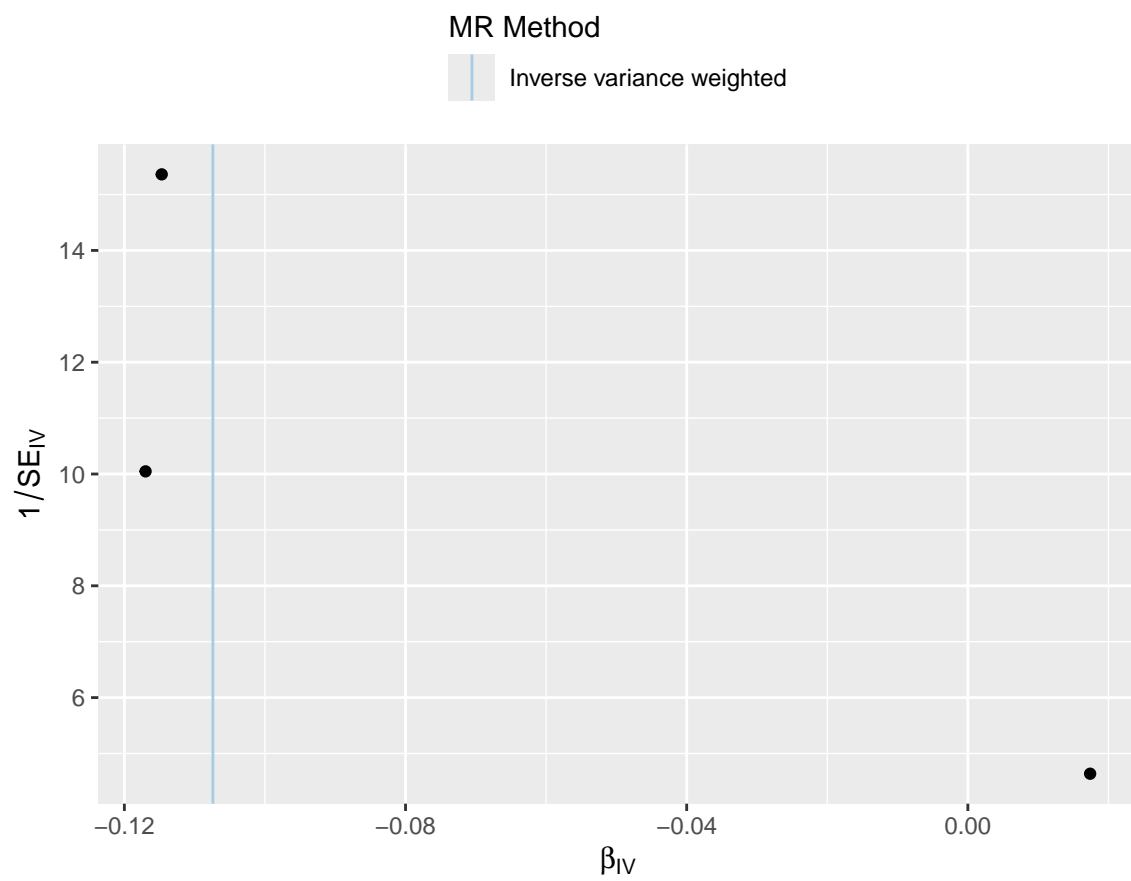

Supplement: Supplementary file 5 [file Datasheet5.zip › Supplementary documents3/mQTL_LOO/cg06380072_LOO_MR.pdf]

cg10108389 – Leave-One-Out MR

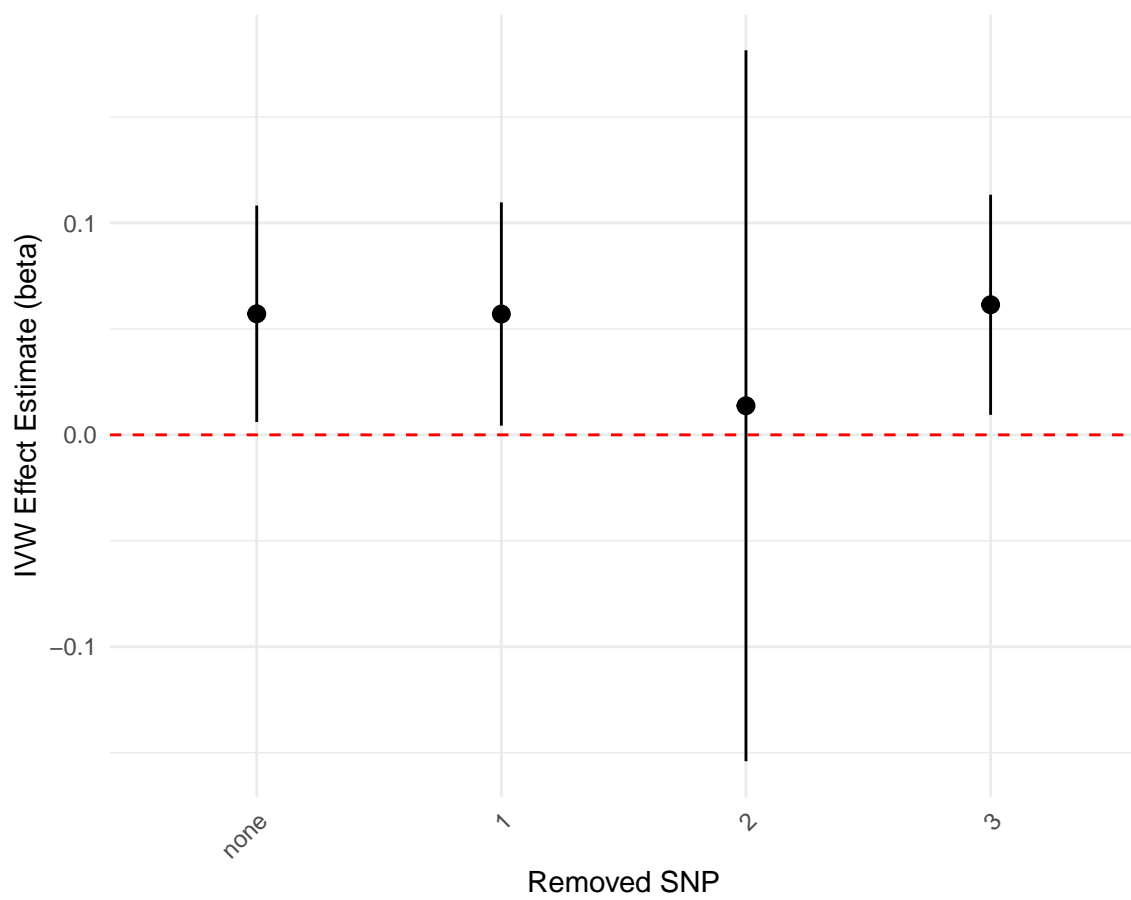

cg10108389 – Forest Plot

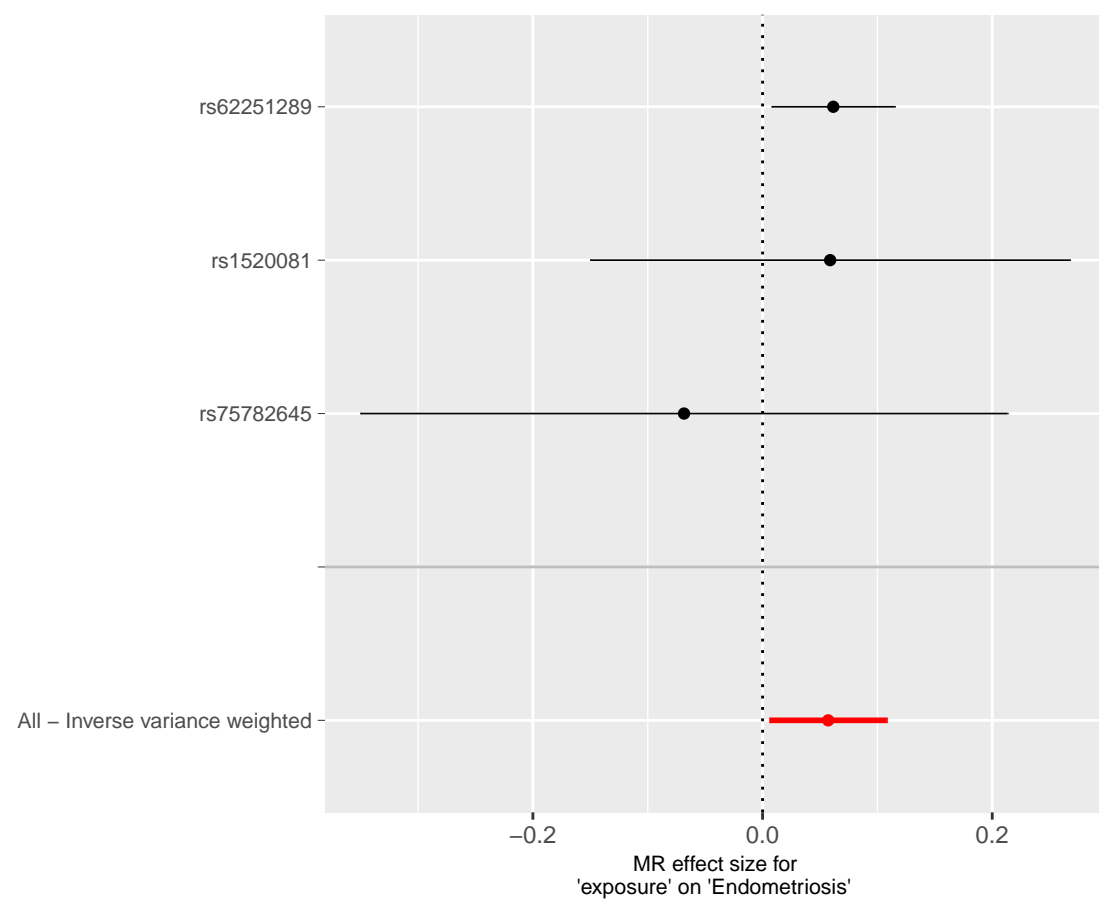

cg10108389 – Funnel Plot

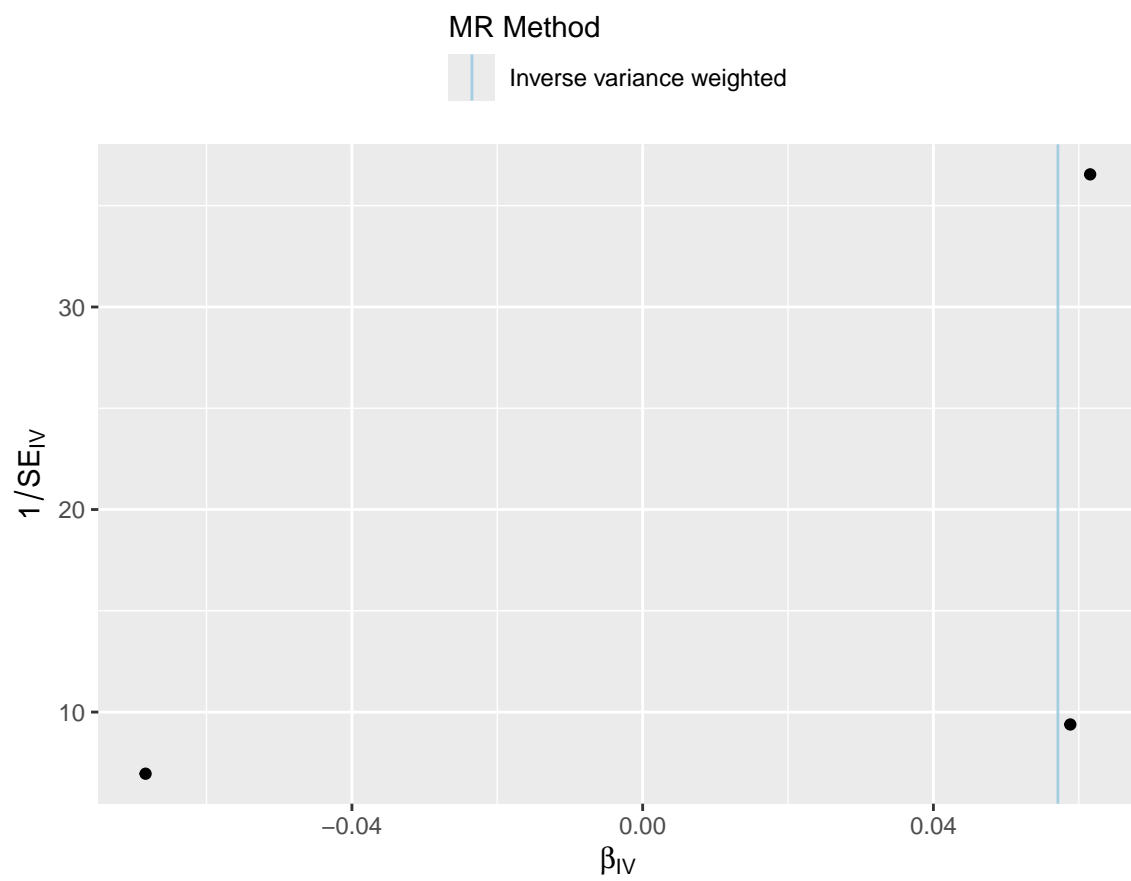

Supplement: Supplementary file 5 [file Datasheet5.zip › Supplementary documents3/mQTL_LOO/cg10108389_LOO_MR.pdf]

cg12240603 – Leave-One-Out MR

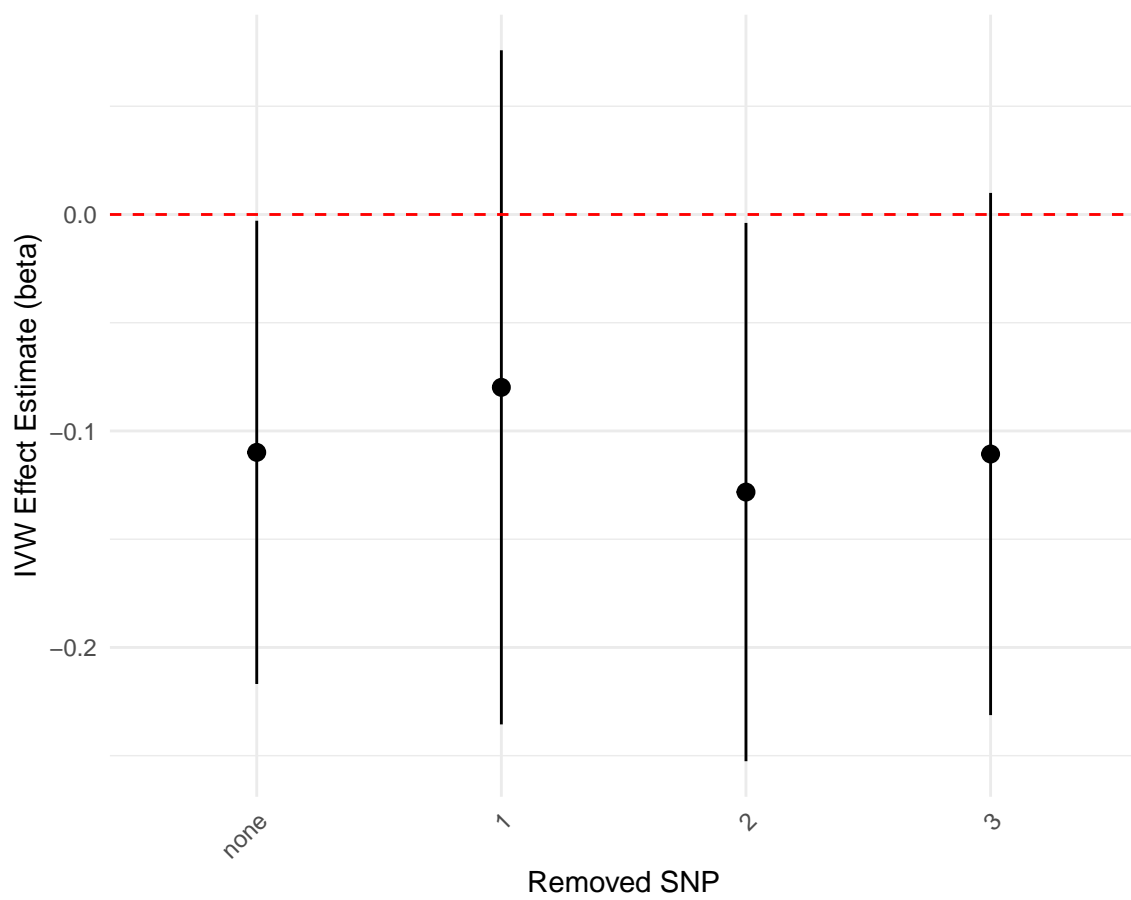

cg12240603 – Forest Plot

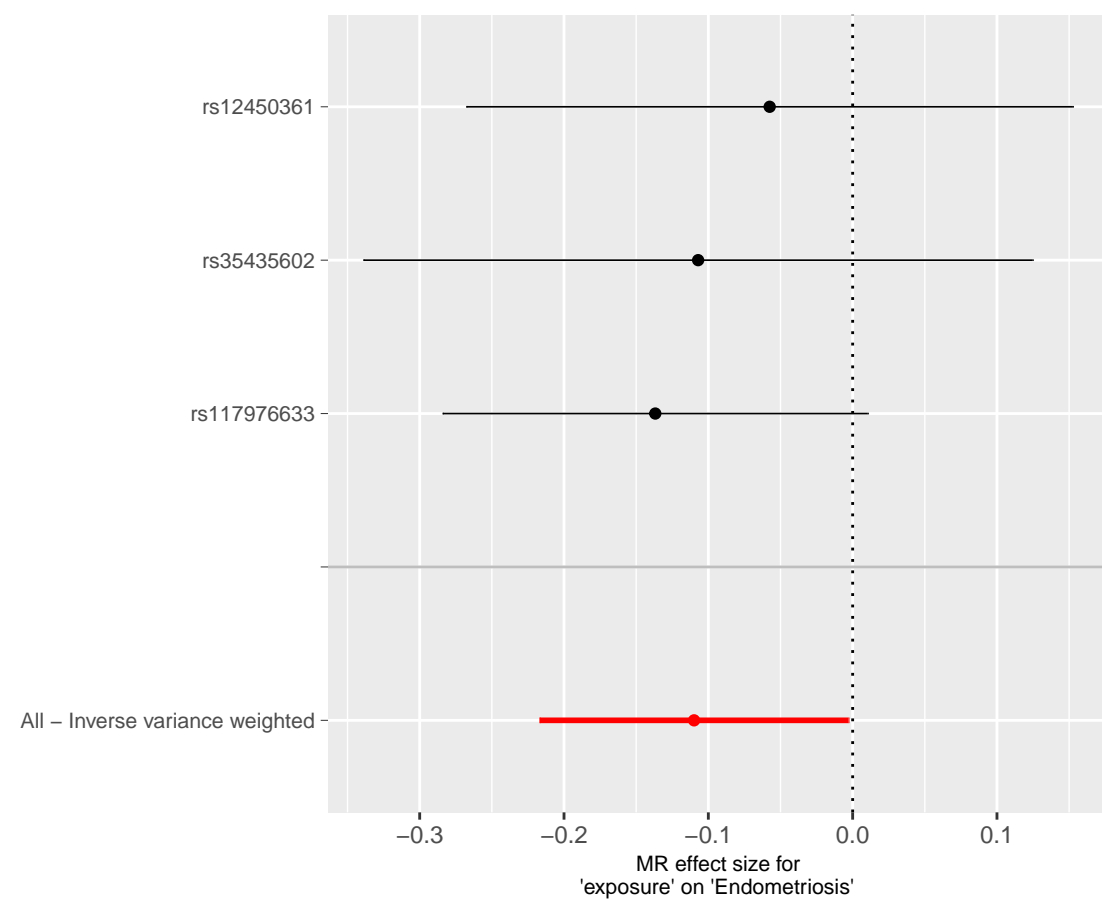

cg12240603 – Funnel Plot

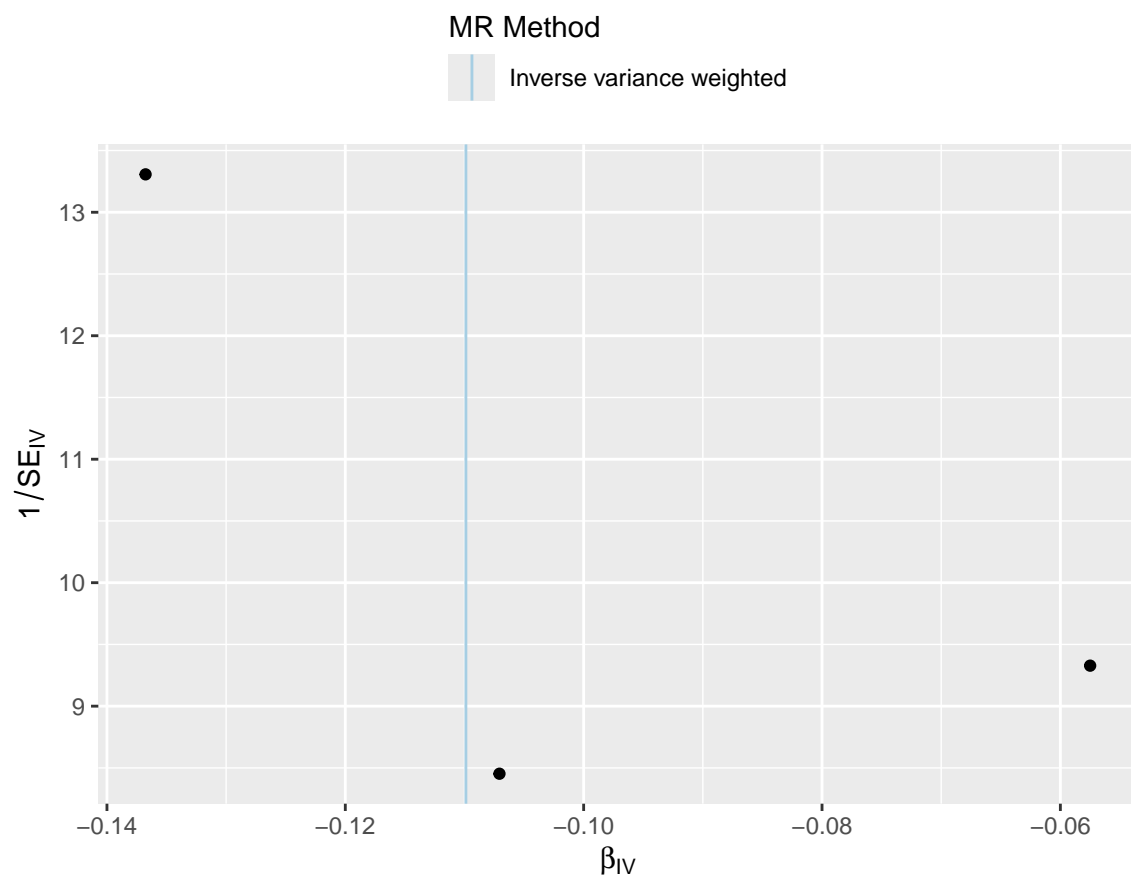

Supplement: Supplementary file 5 [file Datasheet5.zip › Supplementary documents3/mQTL_LOO/cg12240603_LOO_MR.pdf]

cg19912559 – Leave-One-Out MR

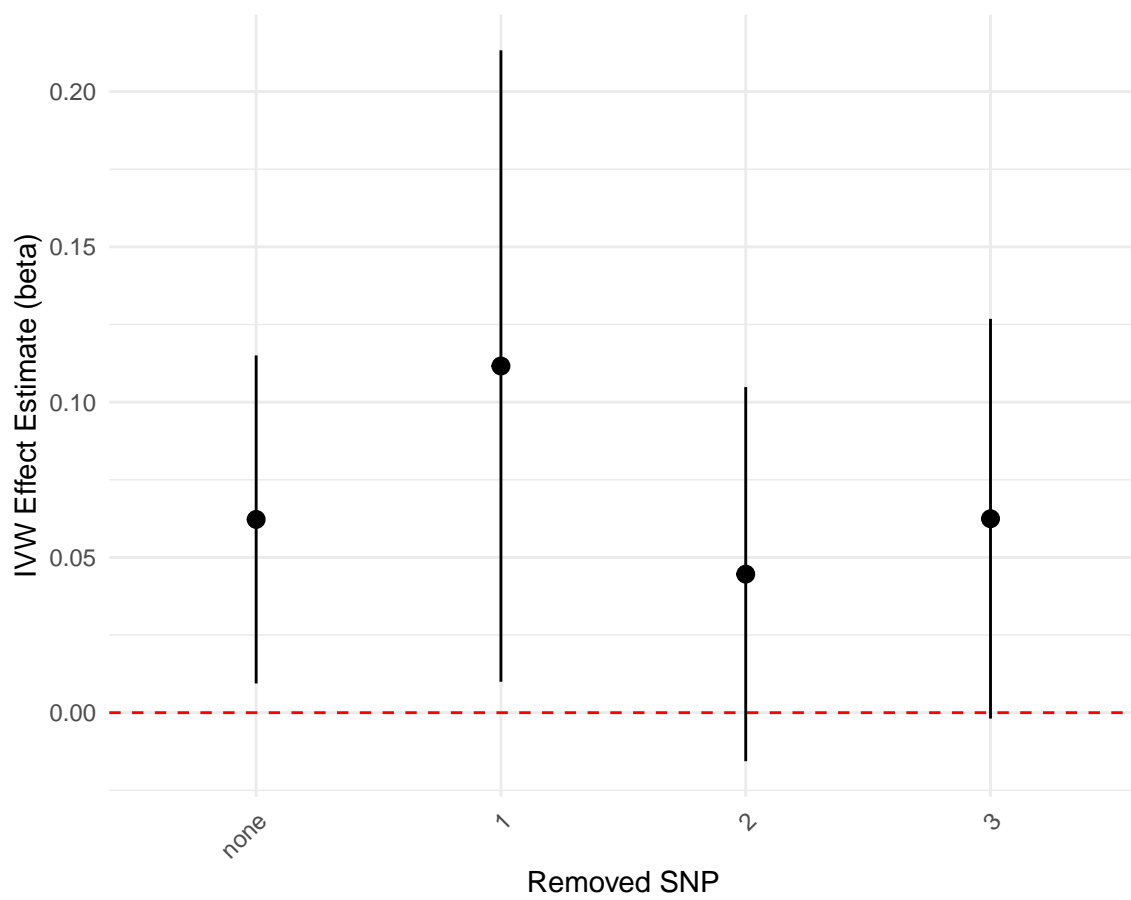

cg19912559 – Forest Plot

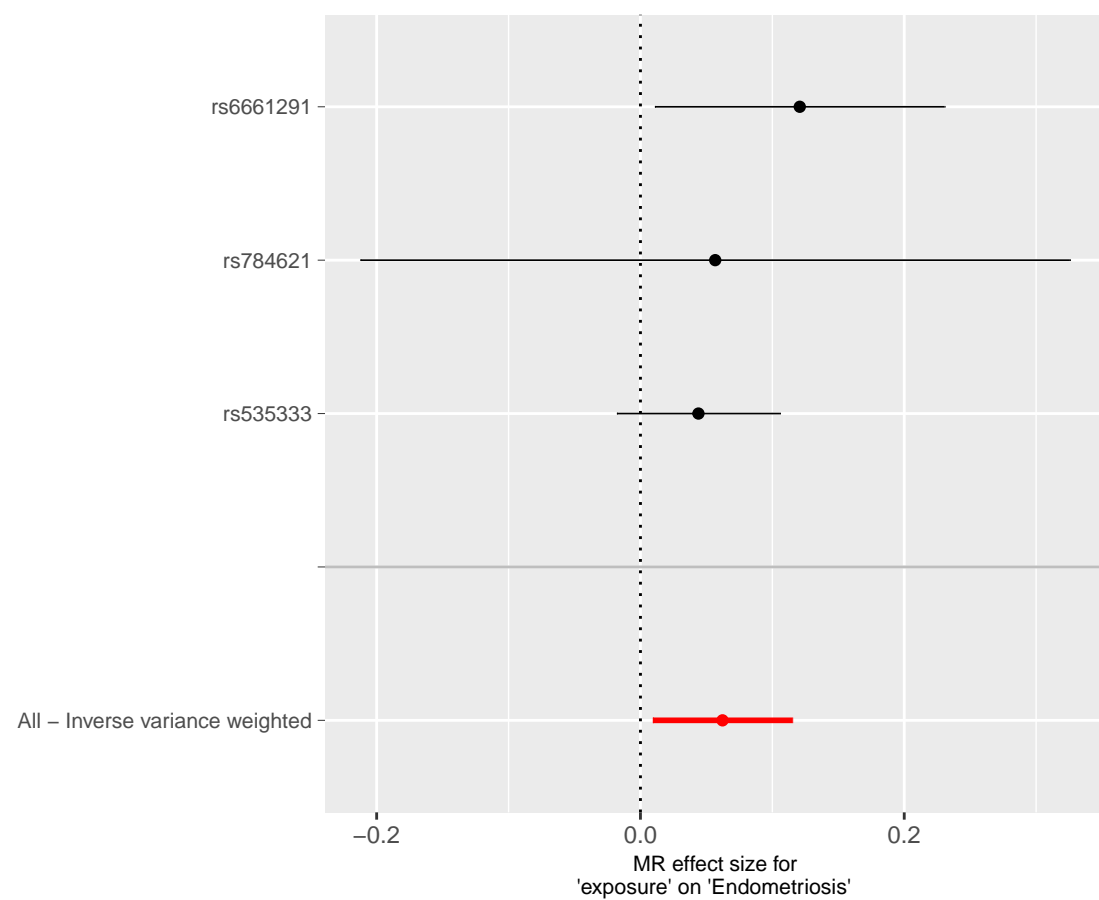

cg19912559 – Funnel Plot

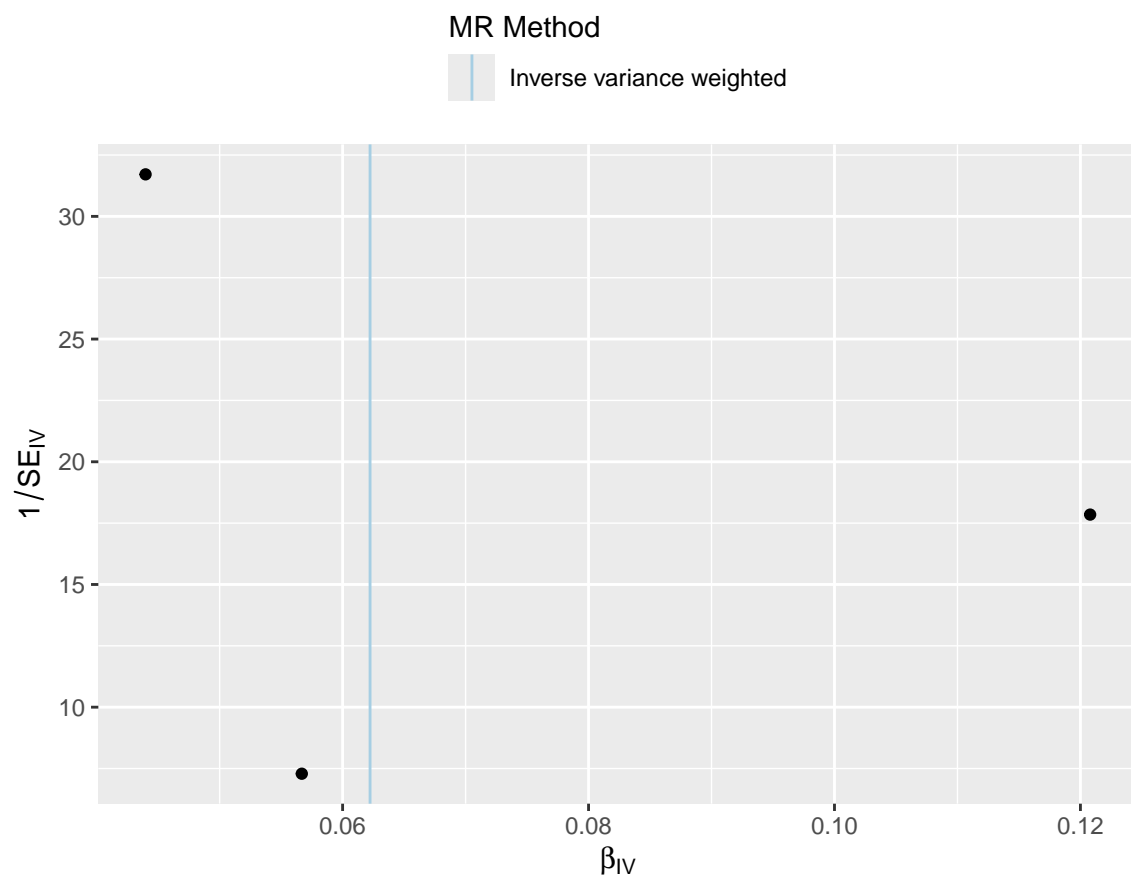

Supplement: Supplementary file 5 [file Datasheet5.zip › Supplementary documents3/mQTL_LOO/cg19912559_LOO_MR.pdf]

cg25195673 – Leave-One-Out MR

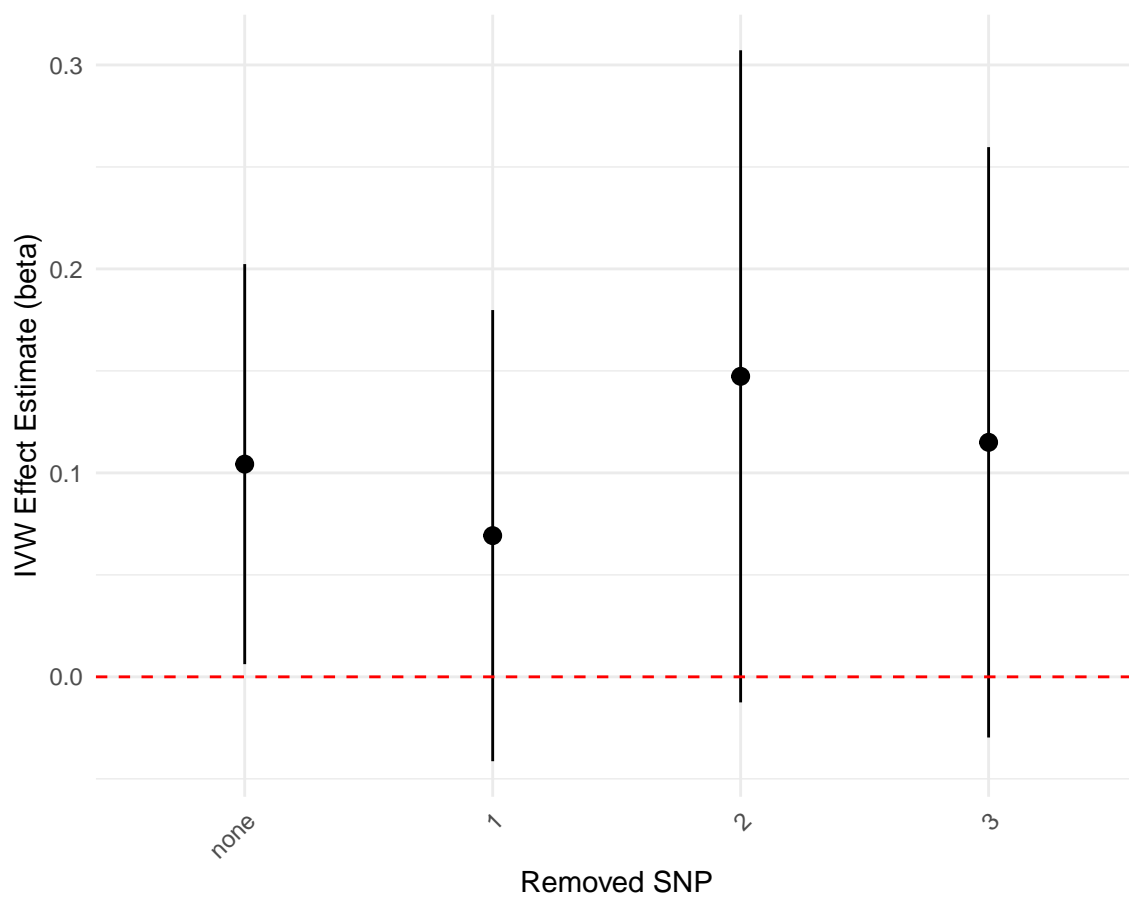

cg25195673 – Funnel Plot

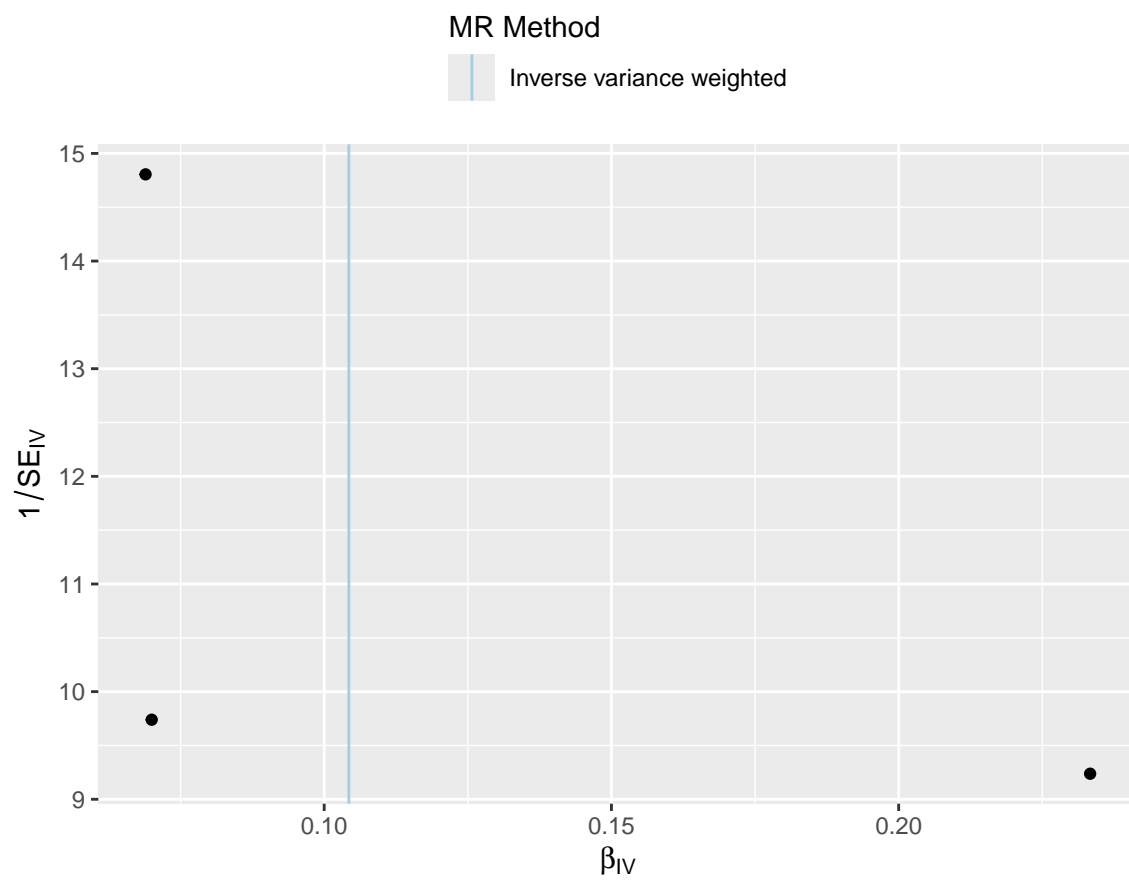

cg25195673 – Forest Plot

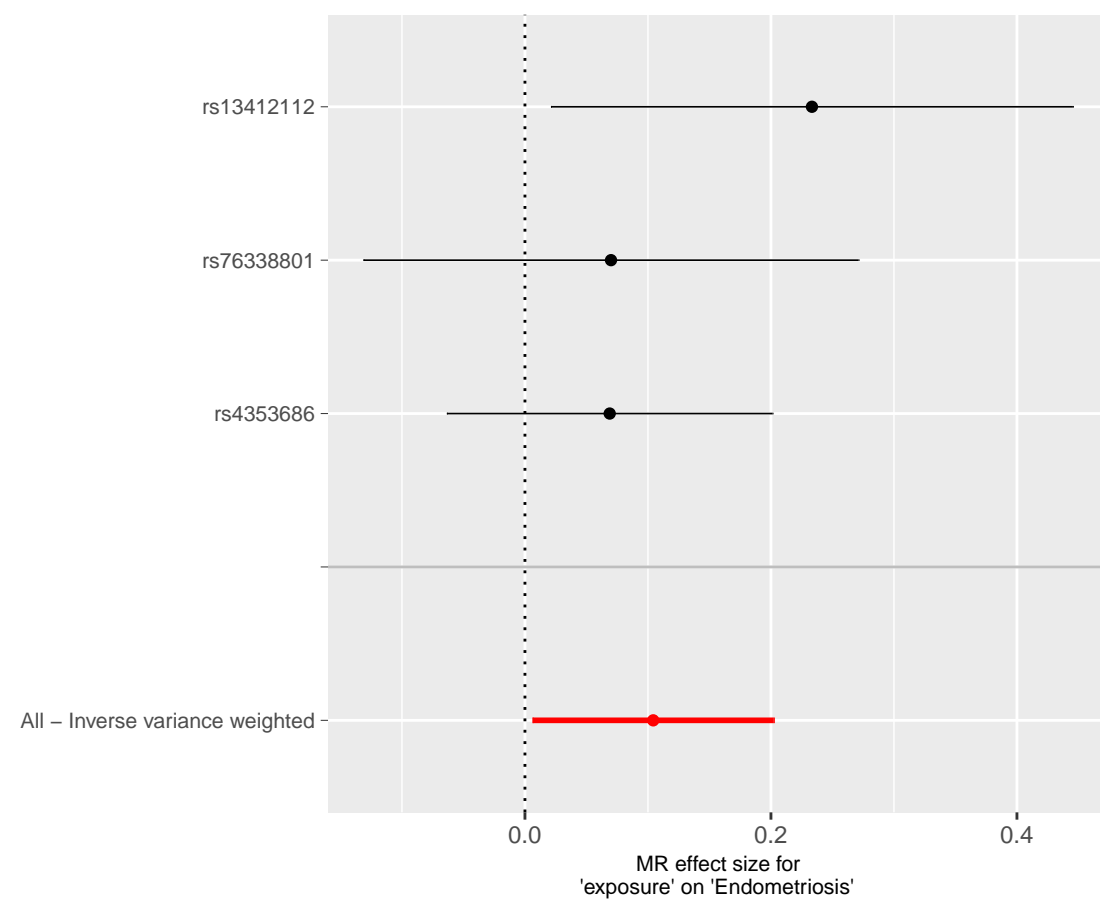

Supplement: Supplementary file 5 [file Datasheet5.zip › Supplementary documents3/mQTL_LOO/cg25195673_LOO_MR.pdf]
